# Supplementary material for: Healing from Within: How Gut Microbiota Predicts IBD Treatment Success—A Systematic Review
Source: Int J Mol Sci. 2024 Aug 2;25(15):8451. doi: 10.3390/ijms25158451 (PMC11313389; doi:10.3390/ijms25158451)
Supplement: Supplementary file 1 [file ijms-25-08451-s001.zip › ijms-3129808-supplementary.pdf]

PUBMED - GUT MICROBIOTA AND IBD (47 RESULTS)	
Systematic Review: The Gut Microbiome and Its Potential Clinical Application in Inflammatory Bowel Disease	10.3390/microorganisms9050977
Rosacea and the Microbiome: A Systematic Review	10.1007/s13555-020-00460-1
A Randomized Trial Comparing the Specific Carbohydrate Diet to a Mediterranean Diet in Adults With Crohn's Disease	10.1053/j.gastro.2021.05.047
Effect of administering kefir on the changes in fecal microbiota and symptoms of inflammatory bowel disease: A randomized controlled trial	10.5152/tjg.2018.18227
Assessing the Relationship between the Gut Microbiota and Inflammatory Bowel Disease Therapeutics: A Systematic Review	10.3390/pathogens12020262
Fecal transplantation for treatment of inflammatory bowel disease	10.1002/14651858.CD012774.pub3
Gut Microbiota and Metabolic Specificity in Ulcerative Colitis and Crohn's Disease	10.3389/fmed.2020.606298
Fecal microbiota transplantation to maintain remission in Crohn's disease: a pilot randomized controlled study	10.1186/s40168-020-0792-5
Microbiota changes induced by microencapsulated sodium butyrate in patients with inflammatory bowel disease	10.1111/nmo.13914
Probiotics for induction of remission in Crohn's disease	10.1002/14651858.CD006634.pub3
Fecal microbiota transplantation for the treatment of irritable bowel syndrome: A systematic review and meta-analysis	10.3748/wjg.v29.i20.3185
Gut microbiome in chronic rheumatic and inflammatory bowel diseases: Similarities and differences	10.1177/2050640619867555
Alterations in Gut Microbiota as Early Biomarkers for Predicting Inflammatory Bowel Disease Onset and Progression: A Systematic Review	10.7759/cureus.58080
Gut Microbiota Profile in Pediatric Patients With Inflammatory Bowel Disease: A Systematic Review	10.3389/fped.2021.626232
Role of Mitochondria in Inflammatory Bowel Diseases: A Systematic Review	10.3390/ijms242317124
Correlation Between the Gut Microbiome and Immunotherapy Response in Inflammatory Bowel Disease: A Systematic Review of the Literature	10.7759/cureus.16808
Inflammatory proteins may mediate the causal relationship between gut microbiota and inflammatory bowel disease: A mediation and multivariable Mendelian randomization study	10.1097/MD.0000000000038551
The Role of Genetically Engineered Probiotics for Treatment of Inflammatory Bowel Disease: A Systematic Review	10.3390/nu15071566

Features of Gut Microbiome Associated With Responses to Fecal Microbiota Transplantation for Inflammatory Bowel Disease: A Systematic Review	10.3389/fmed.2022.773105
Gut microbiome-associated predictors as biomarkers of response to advanced therapies in inflammatory bowel disease: a systematic review	10.1080/19490976.2023.2287073
Systematic review: microbial manipulation as therapy for primary sclerosing cholangitis	10.1111/apt.17251
The role of gastrointestinal pathogens in inflammatory bowel disease: a systematic review	10.1177/17562848211004493
Systematic Review of the Effects of Oat Intake on Gastrointestinal Health	10.1093/jn/nxab245
Mediterranean Diet to Prevent the Development of Colon Diseases: A Meta-Analysis of Gut Microbiota Studies	10.3390/nu13072234
A Systematic Review and Meta-Analysis of Randomized Controlled Trials of Fecal Microbiota Transplantation for the Treatment of Inflammatory Bowel Disease	10.1155/2022/8266793
The Impact of Dietary Interventions on the Microbiota in Inflammatory Bowel Disease: A Systematic Review	10.1093/ecco-jcc/jjad204
Serological profiling of Crohn's disease and ulcerative colitis patients reveals anti-microbial antibody signatures	10.3748/wjg.v28.i30.4089
Beneficial Effects of Organosulfur Compounds from Allium cepa on Gut Health: A Systematic Review	10.3390/foods10081680
Clostridioides difficile Infection in Inflammatory Bowel Disease Patients: A Systematic Review of Risk Factors and Approach in Management	10.7759/cureus.43134
Protocol for a double-blind, randomised, placebo-controlled pilot study for assessing the feasibility and efficacy of faecal microbiota transplant in a paediatric Crohn's disease population: PediCRaFT Trial	10.1136/bmjopen-2019-030120
Impact of Food Additives on Gut Homeostasis	10.3390/nu11102334
Translational Potential of Metabolomics on Animal Models of Inflammatory Bowel Disease-A Systematic Critical Review	10.3390/ijms21113856
The Impact of Microbiome Interventions on the Progression and Severity of Inflammatory Bowel Disease: A Systematic Review	10.7759/cureus.60786
Population structure discovery in meta-analyzed microbial communities and inflammatory bowel disease using MMUPHin	10.1186/s13059-022-02753-4
Geraniol Treatment for Irritable Bowel Syndrome: A Double-Blind Randomized Clinical Trial	10.3390/nu14194208
Fecal transplantation for treatment of inflammatory bowel disease	10.1002/14651858.CD012774.pub2

Meta-analysis defines predominant shared microbial responses in various diseases and a specific inflammatory bowel disease signal	10.1186/s13059-022-02637-7
Development and Validation of Surveys to Estimate Food Additive Intake	10.3390/nu12030812
Association of Dietary Fiber, Fruit, and Vegetable Consumption with Risk of Inflammatory Bowel Disease: A Systematic Review and Meta-Analysis	10.1093/advances/nmaa145
Effects of pentasa-combined probiotics on the microflora structure and prognosis of patients with inflammatory bowel disease	10.5152/tjg.2019.18426
Low FODMAP Diet for Functional Gastrointestinal Symptoms in Quiescent Inflammatory Bowel Disease: A Systematic Review of Randomized Controlled Trials	10.3390/nu12123648
Daily, oral FMT for long-term maintenance therapy in ulcerative colitis: results of a single-center, prospective, randomized pilot study	10.1186/s12876-021-01856-9
The influence of biologics on the microbiome in immune-mediated inflammatory diseases: A systematic review	10.1016/j.biopha.2021.111904
The Effect of Dietary Interventions on Chronic Inflammatory Diseases in Relation to the Microbiome: A Systematic Review	10.3390/nu13093208
A Systematic Review of Artificial Intelligence and Machine Learning Applications to Inflammatory Bowel Disease, with Practical Guidelines for Interpretation	10.1093/ibd/izac115
Mucosa-Associated Lymphoid Tissue Surgeries as a Possible Risk for Inflammatory Bowel Disease: A Systematic Review and Meta-Analysis	10.14740/gr1672
Efficacy of Bifidobacterium breve Fermented Milk in Maintaining Remission of Ulcerative Colitis	10.1007/s10620-018-4946-2
**PUBMED - IBD AND BIOLOGICAL THERAPIES (101 RESULTS)**	
Systematic review with network meta-analysis: Risk of Herpes zoster with biological therapies and small molecules in inflammatory bowel disease	10.1111/apt.17379
Biologics for Inflammatory Bowel Disease and Their Safety in Pregnancy: A Systematic Review and Meta-analysis	10.1016/j.cgh.2020.09.021
The association between endometriosis and autoimmune diseases: a systematic review and meta-analysis	10.1093/humupd/dmz014
Fecal transplantation for treatment of inflammatory bowel disease	10.1002/14651858.CD012774.pub3
Cost-Effectiveness Analysis of Tofacitinib Compared with Biologics in Biologic-Naïve Patients with Moderate-to-Severe Ulcerative Colitis in Japan	10.1007/s40273-023-01254-x

Safety of Biological Therapies in Elderly Inflammatory Bowel Diseases: A Systematic Review and Meta-Analysis	10.3390/jcm11154422
Comparative efficacy and safety of infliximab and vedolizumab therapy in patients with inflammatory bowel disease: a systematic review and meta-analysis	10.1186/s12876-022-02347-1
Fecal microbiota transplantation to maintain remission in Crohn's disease: a pilot randomized controlled study	10.1186/s40168-020-0792-5
Mirikizumab Regulates Genes Involved in Ulcerative Colitis Disease Activity and Anti-TNF Resistance: Results From a Phase 2 Study	10.14309/ctg.0000000000000578
Resolving Histological Inflammation in Ulcerative Colitis With Mirikizumab in the LUCENT Induction and Maintenance Trial Programmes	10.1093/ecco-jcc/jjad050
Efficacy of Biologic Drugs in Short-Duration Versus Long-Duration Inflammatory Bowel Disease: A Systematic Review and an Individual-Patient Data Meta-Analysis of Randomized Controlled Trials	10.1053/j.gastro.2021.10.037
The Specific Carbohydrate Diet and Diet Modification as Induction Therapy for Pediatric Crohn's Disease: A Randomized Diet Controlled Trial	10.3390/nu12123749
Assessing the Relationship between the Gut Microbiota and Inflammatory Bowel Disease Therapeutics: A Systematic Review	10.3390/pathogens12020262
Effectiveness and Durability of COVID-19 Vaccination in 9447 Patients With IBD: A Systematic Review and Meta-Analysis	10.1016/j.cgh.2022.02.030
Gut microbiome-associated predictors as biomarkers of response to advanced therapies in inflammatory bowel disease: a systematic review	10.1080/19490976.2023.2287073
Pharmacogenetics of Biological Agents Used in Inflammatory Bowel Disease: A Systematic Review	10.3390/biomedicines9121748
Impact of SARS-CoV-2 vaccination in inflammatory bowel disease patients with different biological agents: a systematic review and meta-analysis	10.17235/reed.2022.9264/2022
Cancer Risk in Patients Treated with the JAK Inhibitor Tofacitinib: Systematic Review and Meta-Analysis	10.3390/cancers15082197
Genetic Variants Associated with Biological Treatment Response in Inflammatory Bowel Disease: A Systematic Review	10.3390/ijms25073717
Effects of irradiated biological dressings on second-degree burn wounds	10.4314/ahs.v23i2.41
Anti-Drug Antibody Formation Against Biologic Agents in Inflammatory Bowel Disease: A Systematic Review and Meta-Analysis	10.1007/s40259-021-00507-5
Dermatological Manifestations in Pediatric Inflammatory Bowel Disease	10.3390/medicina56090425

Cost-effectiveness Analysis of Subcutaneous Infliximab for Inflammatory Bowel Diseases in Sequential Biologic Treatment	10.1093/ibd/izac160
The Role of Pharmacogenetics in the Therapeutic Response to Thiopurines in the Treatment of Inflammatory Bowel Disease: A Systematic Review	10.3390/jcm12216742
Comparison the effects and side effects of Covid-19 vaccination in patients with inflammatory bowel disease (IBD): a systematic scoping review	10.1186/s12876-022-02460-1
Investigating fecal microbial transplant as a novel therapy in dogs with inflammatory bowel disease: A preliminary study	10.1371/journal.pone.0276295
Fecal microbiota transplantation for the treatment of irritable bowel syndrome: A systematic review and meta-analysis	10.3748/wjg.v29.i20.3185
Switching between reference adalimumab and biosimilars in chronic immune-mediated inflammatory diseases: A systematic literature review	10.1111/bcp.15101
Cannabis and Canabidinoids on the Inflammatory Bowel Diseases: Going Beyond Misuse	10.3390/ijms21082940
Do interventions for mood improve inflammatory biomarkers in inflammatory bowel disease?: a systematic review and meta-analysis	10.1016/j.ebiom.2023.104910
Patient sex does not affect endoscopic outcomes of biologicals in inflammatory bowel disease but is associated with adverse events	10.1007/s00384-020-03663-2
Protocol for Fecal Microbiota Transplantation in Inflammatory Bowel Disease: A Systematic Review and Meta-Analysis	10.1155/2018/8941340
Safety of SARS-CoV-2 vaccination in patients with inflammatory bowel disease: A systematic review and meta-analysis	10.1016/j.dld.2022.03.005
Efficacy and safety of vedolizumab in the treatment of patients with inflammatory bowel disease: A systematic review and meta-analysis of randomized controlled trials	10.3892/etm.2023.11997
Efficacy and safety of biologics in primary sclerosing cholangitis with inflammatory bowel disease: A systematic review and meta-analysis	10.1097/HC9.0000000000000347
Efficacy and Safety of Mesenchymal Stem/Stromal Cell Therapy for Inflammatory Bowel Diseases: An Up-to-Date Systematic Review	10.3390/biom11010082
Use of biologics for the management of Crohn's disease: IG-IBD technical review based on the GRADE methodology	10.1016/j.dld.2023.02.019
Efficacy of Biologics in the Treatment of Primary Sclerosing Cholangitis Associated With Inflammatory Bowel Disease: A Systematic Review and Meta-Analysis	10.7759/cureus.56182

Safety and efficacy of autologous haematopoietic stem-cell transplantation with low-dose cyclophosphamide mobilisation and reduced intensity conditioning versus standard of care in refractory Crohn's disease (ASTIClite): an open-label, multicentre, randomised controlled trial	10.1016/S2468-1253(23)00460-0
The Association between Genetics and Response to Treatment with Biologics in Patients with Psoriasis, Psoriatic Arthritis, Rheumatoid Arthritis, and Inflammatory Bowel Diseases: A Systematic Review and Meta-Analysis	10.3390/ijms25115793
Predictive value of blood concentration of biologics on endoscopic inactivity in inflammatory bowel disease: A systematic review	10.3748/wjg.v27.i9.886
Fecal microbiota transplantation for recurrent C. difficile infection in patients with inflammatory bowel disease: A systematic review and meta-analysis	10.1016/j.jaut.2023.103036
Withdrawal of immunosuppressant or biologic therapy for patients with quiescent Crohn's disease	10.1002/14651858.CD012540.pub2
Web-Based Video Education to Improve Uptake of Influenza Vaccination and Other Preventive Health Recommendations in Adults With Inflammatory Bowel Disease: Randomized Controlled Trial of Project PREVENT	10.2196/42921
SARS-CoV-2 vaccination in inflammatory bowel disease patients with different biological agents: correspondence	10.17235/reed.2023.9347/2022
Interactive Inflammatory Bowel Disease Biologics Decision Aid Does Not Improve Patient Outcomes Over Static Education: Results From a Randomized Trial	10.14309/ajg.0000000000001866
Conventional therapy for moderate to severe inflammatory bowel disease: A systematic literature review	10.3748/wjg.v25.i9.1142
Systematic review: societal cost of illness of inflammatory bowel disease is increasing due to biologics and varies between continents	10.1111/apt.16445
Effectiveness and safety of SARS-CoV-2 vaccine in Inflammatory Bowel Disease patients: a systematic review, meta-analysis and meta-regression	10.1111/apt.16913
Inflammatory bowel disease in primary immunodeficiency disorders is a heterogeneous clinical entity requiring an individualized treatment strategy: A systematic review	10.1016/j.autrev.2021.102872
Guselkumab provides durable improvement across psoriatic arthritis disease domains: post hoc analysis of a phase 3, randomised, double-blind, placebo-controlled study	10.1136/rmdopen-2023-003977
Response to SARS-CoV-2 vaccination in immune mediated inflammatory diseases: Systematic review and meta-analysis	10.1016/j.autrev.2021.102927
Filgotinib Improved Health-Related Quality of Life and Led to Comprehensive Disease Control in Individuals with Ulcerative Colitis: Data from the SELECTION Trial	10.1093/ecco-jcc/jjad018

COVID-19 Immunization Rates in Patients with Inflammatory Bowel Disease Worldwide: A Systematic Review and Meta-Analysis	10.3390/vaccines11101523
Maintenance of clinical remission with biologics and small molecules in inflammatory bowel disease according to trial design: Meta-analysis	10.1016/j.dld.2023.06.009
Fecal transplantation for treatment of inflammatory bowel disease	10.1002/14651858.CD012774.pub2
Treatments for Crohn's Disease-Associated Bowel Damage: A Systematic Review	10.1016/j.cgh.2018.06.043
Immunomodulatory effects of extracellular vesicles from mesenchymal stromal cells: Implication for therapeutic approach in autoimmune diseases	10.1002/kjm2.12841
Composite outcomes in observational studies of ulcerative colitis: A systematic review and meta-analysis	10.1002/ueg2.12183
Melanoma Risk in Patients Treated With Biologic Therapy for Common Inflammatory Diseases: A Systematic Review and Meta-analysis	10.1001/jamadermatol.2020.1300
Biologics During Pregnancy in Women With Inflammatory Bowel Disease and Risk of Infantile Infections: A Systematic Review and Meta-Analysis	10.14309/ajg.0000000000000910
Certolizumab pegol for maintenance of medically induced remission in Crohn's disease	10.1002/14651858.CD013747.pub2
A Web-Based Telemanagement System for Improving Disease Activity and Quality of Life in Patients With Complex Inflammatory Bowel Disease: Pilot Randomized Controlled Trial	10.2196/11602
Primary Non-Response to Tumor Necrosis Factor Antagonists is Associated with Inferior Response to Second-line Biologics in Patients with Inflammatory Bowel Diseases: A Systematic Review and Meta-analysis	10.1093/ecco-jcc/jjy004
No Superiority of Tacrolimus Suppositories vs Beclomethasone Suppositories in a Randomized Trial of Patients With Refractory Ulcerative Proctitis	10.1016/j.cgh.2019.09.049
Safety of biologics in inflammatory bowel disease patients with COVID-19	10.1007/s00384-021-03977-9
Protocol for a double-blind, randomised, placebo-controlled pilot study for assessing the feasibility and efficacy of faecal microbiota transplant in a paediatric Crohn's disease population: PediCRaFT Trial	10.1136/bmjopen-2019-030120
Immune response to influenza and pneumococcal vaccines in adults with inflammatory bowel disease: A systematic review and meta-analysis of 1429 patients	10.1016/j.vaccine.2022.02.027
Safety of Influenza A H1N1pdm09 Vaccines: An Overview of Systematic Reviews	10.3389/fimmu.2021.740048
Interventions Increase Vaccination Rates in Inflammatory Bowel Disease and Rheumatoid Arthritis: A Systematic Review and Meta-Analysis	10.1007/s10620-023-07903-7

A phase IB/IIA study of remestemcel-L, an allogeneic bone marrow-derived mesenchymal stem cell product, for the treatment of medically refractory ulcerative colitis: an interim analysis	10.1111/codi.16239
Efficacy and safety of biologic agents and tofacitinib in moderate-to-severe ulcerative colitis: A systematic overview of meta-analyses	10.1177/2050640619883566
Use of Platelet-Rich Fibrin in the Treatment of Periodontal Intrabony Defects: A Systematic Review and Meta-Analysis	10.1155/2021/6669168
The incidence of psoriasis among smokers and/or former smokers inflammatory bowel diseases patients treated with tumor necrosis factor antagonist: A systematic review and meta-analysis	10.1097/MD.0000000000027510
The influence of biologics on the microbiome in immune-mediated inflammatory diseases: A systematic review	10.1016/j.biopha.2021.111904
Safety of Biologic Therapy in Older Patients With Immune-Mediated Diseases: A Systematic Review and Meta-analysis	10.1016/j.cgh.2018.12.032
Adalimumab for induction of remission in patients with Crohn's disease: a systematic review and meta-analysis	10.1186/s40001-022-00817-6
Daily, oral FMT for long-term maintenance therapy in ulcerative colitis: results of a single-center, prospective, randomized pilot study	10.1186/s12876-021-01856-9
Comparative safety of systemic and low-bioavailability steroids in inflammatory bowel disease: Systematic review and network meta-analysis	10.1111/bcp.13456
Adrenomedullin for biologic-resistant Crohn's disease: A randomized, double-blind, placebo-controlled phase 2a clinical trial	10.1111/jgh.15945
Comparative Risk of Serious Infections With Biologic and/or Immunosuppressive Therapy in Patients With Inflammatory Bowel Diseases: A Systematic Review and Meta-Analysis	10.1016/j.cgh.2019.02.044
Systematic review: hepatosplenic T-cell lymphoma on biologic therapy for inflammatory bowel disease, including data from the Food and Drug Administration Adverse Event Reporting System	10.1111/apt.15637
Methotrexate Is Not Superior to Placebo in Maintaining Steroid-Free Response or Remission in Ulcerative Colitis	10.1053/j.gastro.2018.06.046
Efficacy and safety of fecal microbiota transplant for recurrent Clostridium difficile infection in inflammatory bowel disease: a systematic review and meta-analysis	10.17235/reed.2022.8814/2022
Effects of Apremilast, an Oral Inhibitor of Phosphodiesterase 4, in a Randomized Trial of Patients With Active Ulcerative Colitis	10.1016/j.cgh.2019.12.032
Use of Faecal Transplantation with a Novel Diet for Mild to Moderate Active Ulcerative Colitis: The CRAFT UC Randomised Controlled Trial	10.1093/ecco-jcc/jjab165

Incidence rates of inflammatory bowel disease in patients with psoriasis, psoriatic arthritis and ankylosing spondylitis treated with secukinumab: a retrospective analysis of pooled data from 21 clinical trials	10.1136/annrheumdis-2018-214273
Smad7 Antisense Oligonucleotide-Based Therapy in Crohn's Disease: Is it Time to Re-Evaluate?	10.1007/s40291-022-00606-1
Efficacy and safety of fecal microbiota transplantation by washed preparation in patients with moderate to severely active ulcerative colitis	10.1111/1751-2980.12938
Early combined immunosuppression may be effective and safe in older patients with Crohn's disease: post hoc analysis of REACT	10.1111/apt.15214
Individualized home-monitoring of disease activity in adult patients with inflammatory bowel disease can be recommended in clinical practice: A randomized-clinical trial	10.3748/wjg.v25.i40.6158
Geraniol Treatment for Irritable Bowel Syndrome: A Double-Blind Randomized Clinical Trial	10.3390/nu14194208
A systematic review of nano formulation of natural products for the treatment of inflammatory bowel disease: drug delivery and pharmacological targets	10.1007/s40199-018-0222-4
Effects of pentasa-combined probiotics on the microflora structure and prognosis of patients with inflammatory bowel disease	10.5152/tjg.2019.18426
Low FODMAP Diet for Functional Gastrointestinal Symptoms in Quiescent Inflammatory Bowel Disease: A Systematic Review of Randomized Controlled Trials	10.3390/nu12123648
Daily, oral FMT for long-term maintenance therapy in ulcerative colitis: results of a single-center, prospective, randomized pilot study	10.1186/s12876-021-01856-9
The influence of biologics on the microbiome in immune-mediated inflammatory diseases: A systematic review	10.1016/j.biopha.2021.111904
The Effect of Dietary Interventions on Chronic Inflammatory Diseases in Relation to the Microbiome: A Systematic Review	10.3390/nu13093208
A Systematic Review of Artificial Intelligence and Machine Learning Applications to Inflammatory Bowel Disease, with Practical Guidelines for Interpretation	10.1093/ibd/izac115
Mucosa-Associated Lymphoid Tissue Surgeries as a Possible Risk for Inflammatory Bowel Disease: A Systematic Review and Meta-Analysis	10.14740/gr1672
Efficacy of Bifidobacterium breve Fermented Milk in Maintaining Remission of Ulcerative Colitis	10.1007/s10620-018-4946-2
**PUBMED - GUT MICROBIOME AND BIOLOGICAL THERAPIES (195 RESULTS)**	
Gut microbiome-related effects of berberine and probiotics on type 2 diabetes (the PREMOTE study)	10.1038/s41467-020-18414-8

Post-Antibiotic Gut Mucosal Microbiome Reconstitution Is Impaired by Probiotics and Improved by Autologous FMT	10.1016/j.cell.2018.08.047
Understanding the gut microbiota and sarcopenia: a systematic review	10.1002/jcsm.12784
Comparative of the effectiveness and safety of biological agents, small molecule drugs, and microbiome therapies in ulcerative colitis: Systematic review and network meta-analysis	10.1097/MD.0000000000035689
Whole grain-rich diet reduces body weight and systemic low-grade inflammation without inducing major changes of the gut microbiome: a randomised cross-over trial	10.1136/gutjnl-2017-314786
Gut Microbiome Fermentation Determines the Efficacy of Exercise for Diabetes Prevention	10.1016/j.cmet.2019.11.001
Gut microbiota modulate distal symmetric polyneuropathy in patients with diabetes	10.1016/j.cmet.2023.06.010
Long-term benefit of Microbiota Transfer Therapy on autism symptoms and gut microbiota	10.1038/s41598-019-42183-0
Fecal microbiota transplant overcomes resistance to anti-PD-1 therapy in melanoma patients	10.1126/science.abf3363
CAR-T cell therapy-related cytokine release syndrome and therapeutic response is modulated by the gut microbiome in hematologic malignancies	10.1038/s41467-022-32960-3
Gut OncoMicrobiome Signatures (GOMS) as next-generation biomarkers for cancer immunotherapy	10.1038/s41571-023-00785-8
Gut microbes in cerebrovascular diseases: Gut flora imbalance, potential impact mechanisms and promising treatment strategies	10.3389/fimmu.2022.975921
Effects of Fecal Microbiota Transplantation With Oral Capsules in Obese Patients	10.1016/j.cgh.2019.07.006
Effect of Fecal Microbiota Transplantation on Non-Alcoholic Fatty Liver Disease: A Randomized Clinical Trial	10.3389/fcimb.2022.759306
Effects of Fecal Microbiome Transfer in Adolescents With Obesity: The Gut Bugs Randomized Controlled Trial	10.1001/jamanetworkopen.2020.30415
Intestinal microbiota signatures of clinical response and immune-related adverse events in melanoma patients treated with anti-PD-1	10.1038/s41591-022-01698-2
Fecal microbiota transplantation in puppies with canine parvovirus infection	10.1111/jvim.15072
Immunoregulatory role of the gut microbiota in inflammatory depression	10.1038/s41467-024-47273-w
Impact of Fecal Microbiota Transplantation on Gut Bacterial Bile Acid Metabolism in Humans	10.3390/nu14245200
Compositional and functional differences of the mucosal microbiota along the intestine of healthy individuals	10.1038/s41598-020-71939-2
Dietary supplementation with inulin-propionate ester or inulin improves insulin sensitivity in adults with overweight and obesity with distinct effects on the gut microbiota, plasma metabolome and systemic inflammatory responses: a randomised cross-over trial	10.1136/gutjnl-2019-318424

Effect of Fecal Microbiota Transplantation on 8-Week Remission in Patients With Ulcerative Colitis: A Randomized Clinical Trial	10.1001/jama.2018.20046
Effects of microbiota-directed foods in gnotobiotic animals and undernourished children	10.1126/science.aau4732
Small intestinal microbial dysbiosis underlies symptoms associated with functional gastrointestinal disorders	10.1038/s41467-019-09964-7
Convergent application of traditional Chinese medicine and gut microbiota in ameliorate of cirrhosis: a data mining and Mendelian randomization study	10.3389/fcimb.2023.1273031
Variability of strain engraftment and predictability of microbiome composition after fecal microbiota transplantation across different diseases	10.1038/s41591-022-01964-3
Effects of Diet-Modulated Autologous Fecal Microbiota Transplantation on Weight Regain	10.1053/j.gastro.2020.08.041
Gut microbiome-targeted therapies in nonalcoholic fatty liver disease: a systematic review, meta-analysis, and meta-regression	10.1093/ajcn/nqz042
Fecal transplantation for treatment of inflammatory bowel disease	10.1002/14651858.CD012774.pub3
Fecal microbiota transplantation promotes reduction of antimicrobial resistance by strain replacement	10.1126/scitranslmed.abo2750
Gut microbiome-associated predictors as biomarkers of response to advanced therapies in inflammatory bowel disease: a systematic review	10.1080/19490976.2023.2287073
Faecal microbiota transplantation halts progression of human new-onset type 1 diabetes in a randomised controlled trial	10.1136/gutjnl-2020-322630
Gut fermentation syndrome: A systematic review of case reports	10.1002/ueg2.12062
Efficacy of faecal microbiota transplantation for patients with irritable bowel syndrome in a randomised, double-blind, placebo-controlled study	10.1136/gutjnl-2019-319630
Encyclopedia of fecal microbiota transplantation: a review of effectiveness in the treatment of 85 diseases	10.1097/CM9.0000000000002339
Effect of antibiotic pretreatment on bacterial engraftment after Fecal Microbiota Transplant (FMT) in IBS-D	10.1080/19490976.2021.2020067
Fecal microbiota transplantation for the improvement of metabolism in obesity: The FMT-TRIM double-blind placebo-controlled pilot trial	10.1371/journal.pmed.1003051
Distinct Fecal and Plasma Metabolites in Children with Autism Spectrum Disorders and Their Modulation after Microbiota Transfer Therapy	10.1128/mSphere.00314-20
Oral administration of maternal vaginal microbes at birth to restore gut microbiome development in infants born by caesarean section: A pilot randomised placebo-controlled trial	10.1016/j.ebiom.2021.103443

Gut microbiome stability and dynamics in healthy donors and patients with non-gastrointestinal cancers	10.1084/jem.20200606
Fecal microbiota transplantation to maintain remission in Crohn's disease: a pilot randomized controlled study	10.1186/s40168-020-0792-5
Feasibility, Acceptability, and Safety of Faecal Microbiota Transplantation in the Treatment of Major Depressive Disorder: A Pilot Randomized Controlled Trial	10.1177/07067437221150508
Efficacy and safety of fecal microbiota transplantation for the treatment of diseases other than Clostridium difficile infection: a systematic review and meta-analysis	10.1080/19490976.2020.1854640
Dynamic changes of intestinal flora in patients with irritable bowel syndrome combined with anxiety and depression after oral administration of enterobacteria capsules	10.1080/21655979.2021.1999374
Efficacy of fecal microbiota transplantation in patients with Parkinson's disease: clinical trial results from a randomized, placebo-controlled design	10.1080/19490976.2023.2284247
Probiotics-Supplemented Low-Protein Diet for Microbiota Modulation in Patients with Advanced Chronic Kidney Disease (ProLowCKD): Results from a Placebo-Controlled Randomized Trial	10.3390/nu14081637
Effects of microbiome-based interventions on neurodegenerative diseases: a systematic review and meta-analysis	10.1038/s41598-024-59250-w
The Links between Microbiome and Uremic Toxins in Acute Kidney Injury: Beyond Gut Feeling-A Systematic Review	10.3390/toxins12120788
Assessing the Relationship between the Gut Microbiota and Inflammatory Bowel Disease Therapeutics: A Systematic Review	10.3390/pathogens12020262
Randomized, double-blinded, placebo-controlled pilot study: efficacy of faecal microbiota transplantation on chronic fatigue syndrome	10.1186/s12967-023-04227-y
Probiotics and the Potential of Genetic Modification as a Possible Treatment for Food Allergy	10.3390/nu15194159
Fecal Microbial Transplant in Individuals With Immune-Mediated Dry Eye	10.1016/j.ajo.2021.06.022
Gut Microbiome Changes in Patients with Active Left-Sided Ulcerative Colitis after Fecal Microbiome Transplantation and Topical 5-aminosalicylic Acid Therapy	10.3390/cells9102283
Capsulized Fecal Microbiota Transplantation Induces Remission in Patients with Ulcerative Colitis by Gut Microbial Colonization and Metabolite Regulation	10.1128/spectrum.04152-22
The effect of interventions targeting gut microbiota on depressive symptoms: a systematic review and meta-analysis	10.9778/cmajo.20200283
Gut microbiota differs between treatment outcomes early after fecal microbiota transplantation against recurrent Clostridioides difficile infection	10.1080/19490976.2022.2084306

Predicting cancer immunotherapy response from gut microbiomes using machine learning models	10.18632/oncotarget.28252
Fecal microbiota transplantation for irritable bowel syndrome: a systematic review and meta-analysis of randomized controlled trials	10.3389/fimmu.2023.1136343
PEOPLE (NTC03447678), a phase II trial to test pembrolizumab as first-line treatment in patients with advanced NSCLC with PD-L1 <50%: a multiomics analysis	10.1136/jitc-2023-006833
Reduced alcohol preference and intake after fecal transplant in patients with alcohol use disorder is transmissible to germ-free mice	10.1038/s41467-022-34054-6
Specific fungi associated with response to capsulized fecal microbiota transplantation in patients with active ulcerative colitis	10.3389/fcimb.2022.1086885
Gut microbiota and fecal metabolites in sustained unresponsiveness by oral immunotherapy in school-age children with cow's milk allergy	10.1016/j.alit.2023.10.001
Fecal microbiota transplantation for the treatment of irritable bowel syndrome: A systematic review and meta-analysis	10.3748/wjg.v29.i20.3185
Feasibility of a dietary intervention to modify gut microbial metabolism in patients with hematopoietic stem cell transplantation	10.1038/s41591-023-02587-y
Successful weight regain attenuation by autologous fecal microbiota transplantation is associated with non-core gut microbiota changes during weight loss; randomized controlled trial	10.1080/19490976.2023.2264457
Fecal microbiota transplantation treatment of autoimmune-mediated type 1 diabetes: A systematic review	10.3389/fcimb.2022.1075201
Effects of fecal microbiota transplant on DNA methylation in subjects with metabolic syndrome	10.1080/19490976.2021.1993513
Potential of Fecal Microbiota Transplantation to Prevent Acute GVHD: Analysis from a Phase II Trial	10.1158/1078-0432.CCR-23-2369
Effect of a 12-Week Polyphenol Rutin Intervention on Markers of Pancreatic β-Cell Function and Gut Microbiota in Adults with Overweight without Diabetes	10.3390/nu15153360
Fecal Microbiota Transplantation in Irritable Bowel Syndrome: A Systematic Review and Meta-Analysis of Randomized Controlled Trials	10.3390/ijms241914562
Autologous fecal microbiota transplantation can retain the metabolic achievements of dietary interventions	10.1016/j.ejim.2021.03.038
The Commensal Microbe Veillonella as a Marker for Response to an FGF19 Analog in NASH	10.1002/hep.31523
Establishing or Exaggerating Causality for the Gut Microbiome: Lessons from Human Microbiota-Associated Rodents	10.1016/j.cell.2019.12.025
Fecal microbiota transplantation in the treatment of irritable bowel syndrome: a single-center prospective study in Japan	10.1186/s12876-022-02408-5

Gut bacteriophage dynamics during fecal microbial transplantation in subjects with metabolic syndrome	10.1080/19490976.2021.1897217
FOVOCIP study: a multicenter randomized trial of fosfomycin versus ciprofloxacin for febrile neutropenia in hematologic patients-efficacy and microbiologic safety	10.1186/s13063-023-07702-5
Dynamic Colonization of Microbes and Their Functions after Fecal Microbiota Transplantation for Inflammatory Bowel Disease	10.1128/mBio.00975-21
Safety and tolerability of frozen, capsulized autologous faecal microbiota transplantation. A randomized double blinded phase I clinical trial	10.1371/journal.pone.0292132
Protocol for the Gut Bugs Trial: a randomised double-blind placebo-controlled trial of gut microbiome transfer for the treatment of obesity in adolescents	10.1136/bmjopen-2018-026174
Microbiota dynamics in a randomized trial of gut decontamination during allogeneic hematopoietic cell transplantation	10.1172/jci.insight.154344
Recipient microbiome-related features predicting metabolic improvement following fecal microbiota transplantation in adults with severe obesity and metabolic syndrome: a secondary analysis of a phase 2 clinical trial	10.1080/19490976.2024.2345134
Human gut microbiome changes during a 10 week Randomised Control Trial for micronutrient supplementation in children with attention deficit hyperactivity disorder	10.1038/s41598-019-46146-3
Strain engraftment competition and functional augmentation in a multi-donor fecal microbiota transplantation trial for obesity	10.1186/s40168-021-01060-7
Link between gut microbiota and health outcomes in inulin -treated obese patients: Lessons from the Food4Gut multicenter randomized placebo-controlled trial	10.1016/j.clnu.2020.04.005
A Fructo-Oligosaccharide Prebiotic Is Well Tolerated in Adults Undergoing Allogeneic Hematopoietic Stem Cell Transplantation: A Phase I Dose-Escalation Trial	10.1016/j.jtct.2021.07.009
The effect of gastrointestinal microbiome supplementation on immune checkpoint inhibitor immunotherapy: a systematic review	10.1007/s00432-023-04656-8
Effectiveness of Fecal Microbiota Transplantation for Weight Loss in Patients With Obesity Undergoing Bariatric Surgery: A Randomized Clinical Trial	10.1001/jamanetworkopen.2022.47226
Changes in the Progression of Chronic Kidney Disease in Patients Undergoing Fecal Microbiota Transplantation	10.3390/nu16081109
Gut microbiota changes in animal models of spinal cord injury: a preclinical systematic review and meta-analysis	10.1080/07853890.2023.2269379

Engraftment of strictly anaerobic oxygen-sensitive bacteria in irritable bowel syndrome patients following fecal microbiota transplantation does not improve symptoms	10.1080/19490976.2021.1927635
Benefits and Implications of Resveratrol Supplementation on Microbiota Modulations: A Systematic Review of the Literature	10.3390/ijms23074027
Safety and Efficacy of Consolidation Therapy with Ipilimumab Plus Nivolumab after Autologous Stem Cell Transplantation	10.1016/j.jtct.2020.12.026
Combined metabolic activators therapy ameliorates liver fat in nonalcoholic fatty liver disease patients	10.15252/msb.202110459
Effect of fecal microbiota transplant on symptoms of psychiatric disorders: a systematic review	10.1186/s12888-020-02654-5
Long- and short-term effects of fecal microbiota transplantation on antibiotic resistance genes: results from a randomized placebo-controlled trial	10.1080/19490976.2024.2327442
Clinical and biological predictors of response to standardised paediatric colitis therapy (PROTECT): a multicentre inception cohort study	10.1016/S0140-6736(18)32592-3
Gut microbiota diversity after autologous fecal microbiota transfer in acute myeloid leukemia patients	10.1038/s41467-021-23376-6
Efficacy and Safety of Washed Microbiota Transplantation to Treat Patients with Mild-to-Severe COVID-19 and Suspected of Having Gut Microbiota Dysbiosis: Study Protocol for a Randomized Controlled Trial	10.1007/s11596-021-2475-2
Reconstitution of the gut microbiota of antibiotic-treated patients by autologous fecal microbiota transplant	10.1126/scitranslmed.aap9489
Mortality and microbial diversity after allogeneic hematopoietic stem cell transplantation: secondary analysis of a randomized nutritional intervention trial	10.1038/s41598-021-90976-z
Systematic review of donor and recipient predictive biomarkers of response to faecal microbiota transplantation in patients with ulcerative colitis	10.1016/j.ebiom.2022.104088
Fecal Microbiota Transplantation in Patients With Primary Sclerosing Cholangitis: A Pilot Clinical Trial	10.14309/ajg.0000000000000115
Oral insulin immunotherapy in children at risk for type 1 diabetes in a randomised controlled trial	10.1007/s00125-020-05376-1
The Effects of Human Milk Oligosaccharides on Gut Microbiota, Metabolite Profiles and Host Mucosal Response in Patients with Irritable Bowel Syndrome	10.3390/nu13113836
The influence of biologics on the microbiome in immune-mediated inflammatory diseases: A systematic review	10.1016/j.biopha.2021.111904
Fecal microbiota transplantation in HIV: A pilot placebo-controlled study	10.1038/s41467-021-21472-1

Randomized, Placebo-Controlled Trial of Rifaximin Therapy for Lowering Gut-Derived Cardiovascular Toxins and Inflammation in CKD	10.34067/kid.0003942020
Faecal Microbiota Transplantation [FMT] in the Treatment of Chronic Refractory Pouchitis: A Systematic Review and Meta-analysis	10.1093/ecco-jcc/jjad120
Gastrointestinal Microbiome and Mycobiome Changes during Autologous Transplantation for Multiple Myeloma: Results of a Prospective Pilot Study	10.1016/j.bbmt.2019.04.007
Meta-analysis of the gut microbiota in predicting response to cancer immunotherapy in metastatic melanoma	10.1172/jci.insight.140940
An open randomized multicentre Phase 2 trial to assess the safety of DAV132 and its efficacy to protect gut microbiota diversity in hospitalized patients treated with fluoroquinolones	10.1093/jac/dkab474
The NUTRIENT Trial (NUTRitional Intervention among myEloproliferative Neoplasms): Results from a Randomized Phase I Pilot Study for Feasibility and Adherence	10.1158/2767-9764.CRC-23-0380
Examining the Gastrointestinal and Immunomodulatory Effects of the Novel Probiotic Bacillus subtilis DE111	10.3390/ijms22052453
Human gut microbiota is associated with HIV-reactive immunoglobulin at baseline and following HIV vaccination	10.1371/journal.pone.0225622
Microbial functional change is linked with clinical outcomes after capsular fecal transplant in cirrhosis	10.1172/jci.insight.133410
Impact of Fecal Microbiota Transplantation on Obesity and Metabolic Syndrome-A Systematic Review	10.3390/nu11102291
Changes in Fecal Short-Chain Fatty Acids in IBS Patients and Effects of Different Interventions: A Systematic Review and Meta-Analysis	10.3390/nu16111727
Daily, oral FMT for long-term maintenance therapy in ulcerative colitis: results of a single-center, prospective, randomized pilot study	10.1186/s12876-021-01856-9
Species- and strain-level assessment using rrn long-amplicons suggests donor's influence on gut microbial transference via fecal transplants in metabolic syndrome subjects	10.1080/19490976.2022.2078621
Assessing the efficacy and safety of fecal microbiota transplantation and probiotic VSL#3 for active ulcerative colitis: A systematic review and meta-analysis	10.1371/journal.pone.0228846
Third-party fecal microbiota transplantation for high-risk treatment-naïve acute GVHD of the lower GI tract	10.1182/bloodadvances.2024012556
Deep Transcranial Magnetic Stimulation Affects Gut Microbiota Composition in Obesity: Results of Randomized Clinical Trial	10.3390/ijms22094692

Microbial Pathogenesis and Pathophysiology of Alzheimer's Disease: A Systematic Assessment of Microorganisms' Implications in the Neurodegenerative Disease	10.3389/fnins.2021.648484
Responses to faecal microbiota transplantation in female and male patients with irritable bowel syndrome	10.3748/wjg.v27.i18.2219
Contribution of the Gut Microbiota in P28GST-Mediated Anti-Inflammatory Effects: Experimental and Clinical Insights	10.3390/cells8060577
Gut microbiota composition and functional changes in inflammatory bowel disease and irritable bowel syndrome	10.1126/scitranslmed.aap8914
The intestinal microbiome potentially affects thrombin generation in human subjects	10.1111/jth.14699
Third-party fecal microbiota transplantation following allo-HCT reconstitutes microbiome diversity	10.1182/bloodadvances.2018017731
Fecal microbiota transplant delivered via invasive routes in irritable bowel syndrome: A systematic review and meta-analysis of randomized controlled trials	10.1007/s12664-023-01373-5
Effect of summer acupoint application treatment (SAAT) on gut microbiota in healthy Asian adults: A randomized controlled trial	10.1097/MD.0000000000032951
Bacteriophage transfer during faecal microbiota transplantation in Clostridium difficile infection is associated with treatment outcome	10.1136/gutjnl-2017-313952
Delayed gut microbiota development in high-risk for asthma infants is temporarily modifiable by Lactobacillus supplementation	10.1038/s41467-018-03157-4
Effects of both Pro- and Synbiotics in Liver Surgery and Transplantation with Special Focus on the Gut-Liver Axis-A Systematic Review and Meta-Analysis	10.3390/nu12082461
Fecal transplantation for treatment of inflammatory bowel disease	10.1002/14651858.CD012774.pub2
Fecal Microbiota Transplantation in Chronic Pouchitis: A Randomized, Parallel, Double-Blinded Clinical Trial	10.1093/ibd/izab001
The Impact of Antibiotic-Mediated Modification of the Intestinal Microbiome on Outcomes of Allogeneic Hematopoietic Cell Transplantation: Systematic Review and Meta-Analysis	10.1016/j.bbmt.2020.05.011
Multi-omics Analysis of a Fecal Microbiota Transplantation Trial Identifies Novel Aspects of Acute GVHD Pathogenesis	10.1158/2767-9764.CRC-24-0138
Impact of oral administration of single strain Lactococcus lactis spp. cremoris on immune responses to keyhole limpet hemocyanin immunization and gut microbiota: A randomized placebo-controlled trial in healthy volunteers	10.3389/fimmu.2022.1009304
Fecal transplant from vaginally seeded infants decreases intraabdominal adiposity in mice	10.1080/19490976.2024.2353394

A phase 2 trial of the somatostatin analog pasireotide to prevent GI toxicity and acute GVHD in allogeneic hematopoietic stem cell transplant	10.1371/journal.pone.0252995
Health improvements of type 2 diabetic patients through diet and diet plus fecal microbiota transplantation	10.1038/s41598-022-05127-9
The Efficacy of Short-Term Weight Loss Programs and Consumption of Natural Probiotic Bryndza Cheese on Gut Microbiota Composition in Women	10.3390/nu13061753
Profiling the Fungal Microbiome after Fecal Microbiota Transplantation for Graft-versus-Host Disease: Insights from a Phase 1 Interventional Study	10.1016/j.jtct.2022.10.011
Effect of a Higher-Protein Nut versus Higher-Carbohydrate Cereal Enriched Diet on the Gut Microbiomes of Chinese Participants with Overweight and Normoglycaemia or Prediabetes in the Tū Ora Study	10.3390/nu16121971
Effects of fecal microbiota transplantation in subjects with irritable bowel syndrome are mirrored by changes in gut microbiome	10.1080/19490976.2020.1794263
Comprehensive, multisystem, mechanical decolonization of Vancomycin-Resistant Enterococcus and Carbapenem-Resistant Enterobacteriacease without the use of antibiotics	10.1097/MD.0000000000023686
Safety and tolerability of experimental hookworm infection in humans with metabolic disease: study protocol for a phase 1b randomised controlled clinical trial	10.1186/s12902-019-0461-5
Rhamnan sulphate from green algae Monostroma nitidum improves constipation with gut microbiome alteration in double-blind placebo-controlled trial	10.1038/s41598-021-92459-7
Antibiotic exposure windows and the efficacy of immune checkpoint blockers in patients with cancer: a meta-analysis	10.21037/apm-20-2076
Gut microbiome function predicts response to anti-integrin biologic therapy in inflammatory bowel diseases	10.1016/j.chom.2017.04.010
Effects of whole-grain wheat, rye, and lignan supplementation on cardiometabolic risk factors in men with metabolic syndrome: a randomized crossover trial	10.1093/ajcn/nqaa026
Long-term efficacy and safety of monotherapy with a single fresh fecal microbiota transplant for recurrent active ulcerative colitis: a prospective randomized pilot study	10.1186/s12934-021-01513-6
Impact of a Purified Microbiome Therapeutic on Abundance of Antimicrobial Resistance Genes in Patients With Recurrent Clostridioides difficile Infection	10.1093/cid/ciad636
Metagenomic Insights into the Degradation of Resistant Starch by Human Gut Microbiota	10.1128/AEM.01562-18
Survival signal REG3α prevents crypt apoptosis to control acute gastrointestinal graft-versus-host disease	10.1172/JCI99261

Fecal Microbiota Transplant in Cirrhosis Reduces Gut Microbial Antibiotic Resistance Genes: Analysis of Two Trials	10.1002/hep4.1639
Positive Effects of Exercise Intervention without Weight Loss and Dietary Changes in NAFLD-Related Clinical Parameters: A Systematic Review and Meta-Analysis	10.3390/nu13093135
Nutraceuticals in the Modulation of the Intestinal Microbiota: Current Status and Future Directions	10.3389/fphar.2022.841782
Strain-resolved analysis in a randomized trial of antibiotic pretreatment and maintenance dose delivery mode with fecal microbiota transplant for ulcerative colitis	10.1038/s41598-022-09307-5
Protocol for a double-blind, randomised, placebo-controlled pilot study for assessing the feasibility and efficacy of faecal microbiota transplant in a paediatric Crohn's disease population: PediCRaFT Trial	10.1136/bmjopen-2019-030120
Pilot study investigating the effect of enteral and parenteral nutrition on the gastrointestinal microbiome post-allogeneic transplantation	10.1111/bjh.16218
Intestinal Microbiome Changes in Fecal Microbiota Transplant (FMT) vs. FMT Enriched with Lactobacillus in the Treatment of Recurrent Clostridioides difficile Infection	10.1155/2019/4549298
Effect of the Intake of a Traditional Mexican Beverage Fermented with Lactic Acid Bacteria on Academic Stress in Medical Students	10.3390/nu13051551
Guidelines for reporting on animal fecal transplantation (GRAFT) studies: recommendations from a systematic review of murine transplantation protocols	10.1080/19490976.2021.1979878
Microbial Transplantation With Human Gut Commensals Containing CutC Is Sufficient to Transmit Enhanced Platelet Reactivity and Thrombosis Potential	10.1161/CIRCRESAHA.118.313142
Prebiotic Galactooligosaccharide Supplementation in Adults with Ulcerative Colitis: Exploring the Impact on Peripheral Blood Gene Expression, Gut Microbiota, and Clinical Symptoms	10.3390/nu13103598
Lactobacillus reuteri V3401 Reduces Inflammatory Biomarkers and Modifies the Gastrointestinal Microbiome in Adults with Metabolic Syndrome: The PROSIR Study	10.3390/nu11081761
Immune Stimulation Using a Gut Microbe-Based Immunotherapy Reduces Disease Pathology and Improves Barrier Function in Ulcerative Colitis	10.3389/fimmu.2018.02211
Faecal microbiota transplantation for eradicating carriage of multidrug-resistant organisms: a systematic review	10.1016/j.cmi.2019.04.006
Fecal microbiota transplantation in irritable bowel syndrome: A systematic review and meta-analysis	10.1177/2050640619866990
PRIMMO study protocol: a phase II study combining PD-1 blockade, radiation and immunomodulation to tackle cervical and uterine cancer	10.1186/s12885-019-5676-3

Fecal microbiota transplantation improves metabolic syndrome parameters: systematic review with meta-analysis based on randomized clinical trials	10.1016/j.nutres.2020.06.018
A 4-Week Diet Low or High in Advanced Glycation Endproducts Has Limited Impact on Gut Microbial Composition in Abdominally Obese Individuals: The deAGEing Trial	10.3390/ijms23105328
Faecal microbiota transplantation for the treatment of diarrhoea induced by tyrosine-kinase inhibitors in patients with metastatic renal cell carcinoma	10.1038/s41467-020-18127-y
Efficacy of Fecal Microbiota Transplantation in Irritable Bowel Syndrome: A Systematic Review and Meta-Analysis	10.14309/ajg.0000000000000198
Geraniol Treatment for Irritable Bowel Syndrome: A Double-Blind Randomized Clinical Trial	10.3390/nu14194208
Gut microbiome structure and metabolic activity in inflammatory bowel disease	10.1038/s41564-018-0306-4
The TLR9 agonist MGN1703 triggers a potent type I interferon response in the sigmoid colon	10.1038/mi.2017.59
Effects of Regular Kefir Consumption on Gut Microbiota in Patients with Metabolic Syndrome: A Parallel-Group, Randomized, Controlled Study	10.3390/nu11092089
Efficacy and safety of fecal microbiota transplantation for decolonization of intestinal multidrug-resistant microorganism carriage: beyond Clostridioides difficile infection	10.1080/07853890.2019.1662477
Sequential laxative-probiotic usage for treatment of irritable bowel syndrome: a novel method inspired by mathematical modelling of the microbiome	10.1038/s41598-020-75225-z
Increasing the Dose and/or Repeating Faecal Microbiota Transplantation (FMT) Increases the Response in Patients with Irritable Bowel Syndrome (IBS)	10.3390/nu11061415
Supporting the gastrointestinal microenvironment during high-dose chemotherapy and stem cell transplantation by inhibiting IL-1 signaling with anakinra	10.1038/s41598-022-10700-3
Dietary geraniol ameliorates intestinal dysbiosis and relieves symptoms in irritable bowel syndrome patients: a pilot study	10.1186/s12906-018-2403-6
Long-term Outcomes of Fecal Microbiota Transplantation in Patients With Cirrhosis	10.1053/j.gastro.2019.01.033
Experimental infection with the hookworm, Necator americanus, is associated with stable gut microbial diversity in human volunteers with relapsing multiple sclerosis	10.1186/s12915-021-01003-6
The Effect of Allogenic Versus Autologous Fecal Microbiota Transfer on Symptoms, Visceral Perception and Fecal and Mucosal Microbiota in Irritable Bowel Syndrome: A Randomized Controlled Study	10.14309/ctg.0000000000000034
Effect of Vegan Fecal Microbiota Transplantation on Carnitine- and Choline-Derived Trimethylamine-N-Oxide Production and Vascular Inflammation in Patients With Metabolic Syndrome	10.1161/JAHA.117.008342

Gut microbiota plasticity is correlated with sustained weight loss on a low-carb or low-fat dietary intervention	10.1038/s41598-020-58000-y
Clinical efficacy of fecal microbiota transplantation for patients with small intestinal bacterial overgrowth: a randomized, placebo-controlled clinic study	10.1186/s12876-021-01630-x
Faecal Microbiota Transfer - a new concept for treating cytomegalovirus colitis in children with ulcerative colitis	10.26444/aaem/118189
Evaluation of the effect of Lactobacillus reuteri V3401 on biomarkers of inflammation, cardiovascular risk and liver steatosis in obese adults with metabolic syndrome: a randomized clinical trial (PROSIR)	10.1186/s12906-018-2371-x
Heyndrickxia coagulans strain SANK70258 suppresses symptoms of upper respiratory tract infection via immune modulation: a randomized, double-blind, placebo-controlled, parallel-group, comparative study	10.3389/fimmu.2024.1389920
A Double-Blind, Placebo-Controlled Trial to Assess Safety and Tolerability of (Thetanix) Bacteroides thetaiotaomicron in Adolescent Crohn's Disease	10.14309/ctg.0000000000000287
Fecal microbiota transplantation against intestinal colonization by extended spectrum beta-lactamase producing Enterobacteriaceae: a proof of principle study	10.1186/s13104-018-3293-x
**SCIENCEDIRECT - GUT MICROBIOTA AND IBD (381 RESULTS)**	
Achieving healthy aging through gut microbiota-directed dietary intervention: Focusing on microbial biomarkers and host mechanisms,	https://doi.org/10.1016/j.jare.2024.03.005,
Microbiota, metabolites and mucosal immunity as potential targets of traditional Chinese medicine for respiratory diseases based on the lung-gut crosstalk,	https://doi.org/10.1016/j.prmcm.2024.100374,
Are there consistent effects of gut microbiota composition on performance, productivity and condition in poultry?,	https://doi.org/10.1016/j.psj.2024.103752,
Current and future targets for faecal microbiota transplantation,	https://doi.org/10.1016/j.humic.2018.08.004,
Fecal microbiota transplantation in clinical practice: Present controversies and future prospects,	https://doi.org/10.1016/j.hlife.2024.01.006,
Weizmannia coagulans: an ideal probiotic for gut health,	https://doi.org/10.26599/FSHW.2022.9250002,
People are an organic unity: Gut-lung axis and pneumonia,	https://doi.org/10.1016/j.heliyon.2024.e27822,
Microbial Players in Primary Sclerosing Cholangitis: Current Evidence and Concepts,	https://doi.org/10.1016/j.jcmgh.2023.12.005,
Role reversals: non-canonical roles for immune and non-immune cells in the gut,	https://doi.org/10.1016/j.mucimm.2023.11.004,
Vaginal microbiota: Potential targets for vulvovaginal candidiasis infection,	https://doi.org/10.1016/j.heliyon.2024.e27239,

A review: Roles of carbohydrates in human diseases through regulation of imbalanced intestinal microbiota,	https://doi.org/10.1016/j.jff.2020.104197,
Gut bacteria, endocannabinoid system, and marijuana addiction: Novel therapeutic implications,	https://doi.org/10.1016/j.hsr.2023.100144,
Therapeutic application and potential mechanism of plant-derived extracellular vesicles in inflammatory bowel disease,	https://doi.org/10.1016/j.jare.2024.01.035,
Diet, Food, and Nutritional Exposures and Inflammatory Bowel Disease or Progression of Disease: an Umbrella Review,	https://doi.org/10.1016/j.advnut.2024.100219,
Diet and physical activity influence the composition of gut microbiota, benefit on Alzheimer's disease,	https://doi.org/10.26599/FSHW.2022.9250049,
Fecal virome transplantation: A promising strategy for the treatment of metabolic diseases,	https://doi.org/10.1016/j.biopha.2024.117065,
Epithelial regulation of microbiota-immune cell dynamics,	https://doi.org/10.1016/j.mucimm.2024.02.008,
Gut microbiota as an "invisible organ" that modulates the function of drugs,	https://doi.org/10.1016/j.biopha.2019.109653,
Stromal Cell Regulation of Intestinal Inflammatory Fibrosis,	https://doi.org/10.1016/j.jcmgh.2024.01.007,
Human–gut bacterial protein–protein interactions: understudied but impactful to human health,	https://doi.org/10.1016/j.tim.2023.09.009,
The Impact of Dietary Fiber on Gut Microbiota in Host Health and Disease,	https://doi.org/10.1016/j.chom.2018.05.012,
Gut/rumen-mammary gland axis in mastitis: Gut/rumen microbiota–mediated "gastroenterogenic mastitis",	https://doi.org/10.1016/j.jare.2023.02.009,
The role of gut microbiota associated metabolites in digestive disorders,	https://doi.org/10.1016/j.engreg.2024.04.003,
Inflammatory bowel disease and primary sclerosing cholangitis: One disease or two?,	https://doi.org/10.1016/j.jhep.2023.09.031,
Antibiotics and the gut microbiome: Understanding the impact on human health,	https://doi.org/10.1016/j.medmic.2024.100106,
The role of gut-lung axis in COPD: Pathogenesis, immune response, and prospective treatment,	https://doi.org/10.1016/j.heliyon.2024.e30612,
Unraveling host regulation of gut microbiota through the epigenome–microbiome axis,	https://doi.org/10.1016/j.tim.2024.05.006,
Exploring Chinese herbal medicine for the treatment of inflammatory bowel disease: A comprehensive overview,	https://doi.org/10.1016/j.prmcm.2024.100380,
Small intestinal microbiota: from taxonomic composition to metabolism,	https://doi.org/10.1016/j.tim.2024.02.013,
Interaction of microbiota, mucosal malignancies, and immunotherapy—Mechanistic insights,	https://doi.org/10.1016/j.mucimm.2024.03.007,
Inflammatory and immunometabolic consequences of gut dysfunction in HIV: Parallels with IBD and implications for reservoir persistence and non-AIDS comorbidities,	https://doi.org/10.1016/j.ebiom.2019.07.027,
The Role of Early Life Gut Mycobiome on Child Health,	https://doi.org/10.1016/j.advnut.2024.100185,
The influence of the gut-brain axis on anxiety and depression: A review of the literature on the use of probiotics,	https://doi.org/10.1016/j.jtcme.2024.03.011,

Advances in Brain–Gut–Microbiome Interactions: A Comprehensive Update on Signaling Mechanisms, Disorders, and Therapeutic Implications,	https://doi.org/10.1016/j.jcmgh.2024.01.024,
Pain regulation by gut microbiota: molecular mechanisms and therapeutic potential,	https://doi.org/10.1016/j.bja.2019.07.026,
Microbiota and cancer: host cellular mechanisms activated by gut microbial metabolites,	https://doi.org/10.1016/j.ijmm.2020.151425,
The roles of gut microbiota metabolites in the occurrence and development of colorectal cancer: Multiple insights for potential clinical applications,	https://doi.org/10.1016/j.gastha.2024.05.012,
Chromatin dynamics and histone modifications in intestinal microbiota-host crosstalk,	https://doi.org/10.1016/j.molmet.2019.12.005,
Gastrointestinal Biofilms: Endoscopic Detection, Disease Relevance, and Therapeutic Strategies,	https://doi.org/10.1053/j.gastro.2024.04.032,
Influence of gut microbiota on autoimmunity: A narrative review,	https://doi.org/10.1016/j.bbii.2024.100046,
Bacterial consortia-The latest arsenal to inflammatory bowel disease bacteriotherapy,	https://doi.org/10.1016/j.medmic.2024.100107,
Exposure to prescribed medication in early life and impacts on gut microbiota and disease development,	https://doi.org/10.1016/j.eclinm.2024.102428,
The gut microbiota and mental health in adults,	https://doi.org/10.1016/j.conb.2020.01.016,
Gut microbiota nexus: Exploring the interactions with the brain, heart, lungs, and skin axes and their effects on health,	https://doi.org/10.1016/j.medmic.2024.100104,
Natural polysaccharides regulate intestinal microbiota for inhibiting colorectal cancer,	https://doi.org/10.1016/j.heliyon.2024.e31514,
Exploring the effect of gut microbiome on Alzheimer's disease,	https://doi.org/10.1016/j.bbrep.2024.101776,
The antibody/microbiota interface in health and disease,	https://doi.org/10.1038/s41385-019-0192-y,
Unveiling the microbial symphony: Next-Gen sequencing and bioinformatics insights into the human gut microbiome,	https://doi.org/10.1016/j.hsr.2024.100173,
Fecal microbiota transplantation to maintain remission in Crohn's disease: a pilot randomized controlled study	10.1186/s40168-020-0792-5
The regulation of intestinal microbiota and the intervention of Chinese herbal medicine in the treatment of ulcerative colitis,	https://doi.org/10.1016/j.prmcm.2024.100356,
Gut microbiome-metabolites axis: A friend or foe to colorectal cancer progression,	https://doi.org/10.1016/j.biopha.2024.116410,
Methodology, efficacy and safety of fecal microbiota transplantation in treating inflammatory bowel disease,	https://doi.org/10.1016/j.medmic.2020.100028,
Role of skin and gut microbiota in the pathogenesis of psoriasis, an inflammatory skin disease,	https://doi.org/10.1016/j.medmic.2020.100016,
A review of the interaction between diet composition and gut microbiota and its impact on associated disease,	https://doi.org/10.1016/j.jfutfo.2023.07.004,
Diverse mechanisms by which chemical pollutant exposure alters gut microbiota metabolism and inflammation,	https://doi.org/10.1016/j.envint.2024.108805,

Revisit gut microbiota and its impact on human health and disease,	https://doi.org/10.1016/j.jfda.2018.12.012,
The utility of microbiome (microbiota) and exosomes in dentistry,	https://doi.org/10.1016/j.jds.2024.05.019,
Establishing or Exaggerating Causality for the Gut Microbiome: Lessons from Human Microbiota-Associated Rodents,	https://doi.org/10.1016/j.cell.2019.12.025,
The microbiota-immune axis as a central mediator of gut-brain communication,	https://doi.org/10.1016/j.nbd.2019.104714,
Unraveling the gut-Lung axis: Exploring complex mechanisms in disease interplay,	https://doi.org/10.1016/j.heliyon.2024.e24032,
Short-chain fatty acids: Important components of the gut-brain axis against AD,	https://doi.org/10.1016/j.biopha.2024.116601,
Exploring the relationship between Faecalibacterium duncaniae and Escherichia coli in inflammatory bowel disease (IBD): Insights and implications,	https://doi.org/10.1016/j.csbj.2023.11.027,
Dietary proanthocyanidins for improving gut immune health,	https://doi.org/10.1016/j.cofs.2024.101133,
Microbiota and Thyroid Interaction in Health and Disease,	https://doi.org/10.1016/j.tem.2019.05.008,
Effects of heavy metals on gut barrier integrity and gut microbiota,	https://doi.org/10.1530/MAH-23-0015,
Development and establishment of oral microbiota in early life,	https://doi.org/10.1016/j.job.2024.05.001,
Enhancing Clinical Efficacy through the Gut Microbiota: A New Field of Traditional Chinese Medicine,	https://doi.org/10.1016/j.eng.2018.11.013,
Insights on the impact of diet-mediated microbiota alterations on immunity and diseases,	https://doi.org/10.1111/ajt.14477,
Human Gut Microbiota and Gastrointestinal Cancer,	https://doi.org/10.1016/j.gpb.2017.06.002,
Ulcerative colitis: Gut microbiota, immunopathogenesis and application of natural products in animal models,	https://doi.org/10.1016/j.lfs.2020.118129,
Another piece of puzzle for the human microbiome: the gut virome under dietary modulation,	https://doi.org/10.1016/j.jgg.2024.04.013,
Gut-brain axis: A cutting-edge approach to target neurological disorders and potential synbiotic application,	https://doi.org/10.1016/j.heliyon.2024.e34092,
The emerging role of the gut virome in necrotizing enterocolitis,	https://doi.org/10.1016/j.heliyon.2024.e30496,
Ginseng as a therapeutic target to alleviate gut and brain diseases via microbiome regulation,	https://doi.org/10.1016/j.jgr.2024.04.005,
Gut microecology: Why our microbes could be key to our health,	https://doi.org/10.1016/j.biopha.2020.110784,
Digestive tract mycobiota and microbiota and the effects on the immune system,	https://doi.org/10.1016/j.humic.2019.100056,
The microbiome-driven impact of western diet in the development of noncommunicable chronic disorders,	https://doi.org/10.1016/j.bpg.2024.101923,
The interaction between the gut Microbiota and herbal medicines,	https://doi.org/10.1016/j.biopha.2019.109252,
Intestinal Microbiota in Early Life and Its Implications on Childhood Health,	https://doi.org/10.1016/j.gpb.2018.10.002,
Galacto-Oligosaccharides and the Elderly Gut: Implications for Immune Restoration and Health,	https://doi.org/10.1016/j.advnut.2024.100263,
Exploring the oral-gut linkage: Interrelationship between oral and systemic diseases,	https://doi.org/10.1016/j.mucimm.2023.11.006,

Deciphering the gut microbiome: The revolution of artificial intelligence in microbiota analysis and intervention,	https://doi.org/10.1016/j.crbiot.2024.100211,
Long term management of ulcerative colitis with Faecal Microbiota Transplantation,	https://doi.org/10.1016/j.medmic.2020.100026,
From Network Analysis to Functional Metabolic Modeling of the Human Gut Microbiota,	https://doi.org/10.1128/msystems.00209-17,
Host-Gut Microbiota Crosstalk in Intestinal Adaptation,	https://doi.org/10.1016/j.jcmgh.2018.01.024,
Diabetic gut microbiota dysbiosis as an inflammaging and immunosenescence condition that fosters progression of retinopathy and nephropathy,	https://doi.org/10.1016/j.bbadis.2018.09.032,
Pathobionts in Inflammatory Bowel Disease: Origins, Underlying Mechanisms, and Implications for Clinical Care,	https://doi.org/10.1053/j.gastro.2023.09.019,
Implications of the gut microbiome in cardiovascular diseases: Association of gut microbiome with cardiovascular diseases, therapeutic interventions and multi-omics approach for precision medicine,	https://doi.org/10.1016/j.medmic.2023.100096,
Fermented foods: Harnessing their potential to modulate the microbiota-gut-brain axis for mental health,	https://doi.org/10.1016/j.neubiorev.2024.105562,
Potential effects of the most prescribed drugs on the microbiota-gut-brain-axis: A review,	https://doi.org/10.1016/j.brainresbull.2024.110883,
Metaproteomics of the human gut microbiota: Challenges and contributions to other OMICS,	https://doi.org/10.1016/j.clinms.2019.06.001,
Gut microbiota disparities between active Crohn's disease and healthy controls: A global systematic review,	https://doi.org/10.1016/j.cegh.2023.101497,
The Gut Microbiota at the Service of Immunometabolism,	https://doi.org/10.1016/j.cmet.2020.09.004,
Carcinogenesis as a Result of Multiple Inflammatory and Oxidative Hits: a Comprehensive Review from Tumor Microenvironment to Gut Microbiota,	https://doi.org/10.1016/j.neo.2018.05.002,
Gut microbiota and their derivatives in the progression of colorectal cancer: Mechanisms of action, genome and epigenome contributions,	https://doi.org/10.1016/j.heliyon.2024.e29495,
Microbiota and Fatty Liver Disease—the Known, the Unknown, and the Future,	https://doi.org/10.1016/j.chom.2020.07.007,
Interplay between gut microbiota and antimicrobial peptides,	https://doi.org/10.1016/j.aninu.2020.09.002,
Pursuing Human-Relevant Gut Microbiota-Immune Interactions,	https://doi.org/10.1016/j.immuni.2019.08.002,
Gut Microbiota Regulation of Tryptophan Metabolism in Health and Disease,	https://doi.org/10.1016/j.chom.2018.05.003,
Gut microbiota in children and altered profiles in juvenile idiopathic arthritis,	https://doi.org/10.1016/j.jaut.2019.01.001,
Harnessing the microbiota for therapeutic purposes,	https://doi.org/10.1111/ajt.15753,
Diet, Microbiota, and Colorectal Cancer,	https://doi.org/10.1016/j.isci.2019.10.011,
From ASCA breakthrough in Crohn's disease and Candida albicans research to thirty years of investigations about their meaning in human health,	https://doi.org/10.1016/j.autrev.2023.103486,

Fecal microbiota transplantation beyond Clostridioides difficile infections,	https://doi.org/10.1016/j.ebiom.2019.05.066,
The â€œGut Feelingâ€ : Breaking Down the Role of Gut Microbiome in Multiple Sclerosis,	https://doi.org/10.1007/s13311-017-0588-x,
Candida gut commensalism and inflammatory disease,	https://doi.org/10.1016/j.medmic.2020.100008,
Cervicovaginal Microbiota and Reproductive Health: The Virtue of Simplicity,	https://doi.org/10.1016/j.chom.2018.01.013,
Intestinal Permeability in Relapsing-Remitting Multiple Sclerosis,	https://doi.org/10.1007/s13311-017-0582-3,
Oxidative Stress and Redox-Modulating Therapeutics in Inflammatory Bowel Disease,	https://doi.org/10.1016/j.molmed.2020.06.006,
Bacteriophages of the Human Gut: The â€œKnown Unknownâ€ of the Microbiome,	https://doi.org/10.1016/j.chom.2019.01.017,
Probiotics for the treatment of ulcerative colitis: a review of experimental research from 2018 to 2022	10.3389/fmicb.2023.1211271
Genome engineering of the human gut microbiome,	https://doi.org/10.1016/j.jgg.2024.01.002,
Long non-coding RNAs as pathophysiological regulators, therapeutic targets and novel extracellular vesicle biomarkers for the diagnosis of inflammatory bowel disease,	https://doi.org/10.1016/j.biopha.2024.116868,
Design strategies, advances and future perspectives of colon-targeted delivery systems for the treatment of inflammatory bowel disease,	https://doi.org/10.1016/j.ajps.2024.100943,
A review on the use of prebiotics in ulcerative colitis,	https://doi.org/10.1016/j.tim.2023.11.007,
Adjunctive fecal microbiota transplantation in supportive oncology: Emerging indications and considerations in immunocompromised patients,	https://doi.org/10.1016/j.ebiom.2019.03.070,
Sphingosine-1-phosphate signaling and the gut-liver axis in liver diseases,	https://doi.org/10.1016/j.livres.2019.02.003,
IgA Responses to Microbiota,	https://doi.org/10.1016/j.immuni.2018.08.011,
Gut-liver axis: Pathophysiological concepts and medical perspective in chronic liver diseases,	https://doi.org/10.1016/j.smim.2023.101859,
Metabolomic profiling in children with inflammatory bowel disease,	https://doi.org/10.1016/j.advms.2019.12.009,
Autophagy: A potential target for natural products in the treatment of ulcerative colitis,	https://doi.org/10.1016/j.biopha.2024.116891,
Host responses to mucosal biofilms in the lung and gut,	https://doi.org/10.1038/s41385-020-0270-1,
Microbiome in cancer: A comparative analysis between humans and dogs,	https://doi.org/10.1016/j.tvjl.2024.106145,
Dietary legumes, intestinal microbiota, inflammation and colorectal cancer,	https://doi.org/10.1016/j.jff.2019.103707,
Microbiome and colorectal cancer: Roles in carcinogenesis and clinical potential,	https://doi.org/10.1016/j.mam.2019.05.001,
Lipid- and polymer-based formulations containing TNF-Î± inhibitors for the treatment of inflammatory bowel diseases,	https://doi.org/10.1016/j.drudis.2024.104090,
Anxiety, Depression, and the Microbiome: A Role for Gut Peptides,	https://doi.org/10.1007/s13311-017-0585-0,
The gut microbiome and epilepsy,	https://doi.org/10.1016/j.ebiom.2019.05.024,
Inferring composition and function of the human gut microbiome in time and space: A review of genome-scale metabolic modelling tools,	https://doi.org/10.1016/j.csbj.2020.11.035,

Implications of butyrate and its derivatives for gut health and animal production,	https://doi.org/10.1016/j.aninu.2017.08.010,
Association between the microbiota and womenâ€™s cancers â€“ Cause or consequences?,	https://doi.org/10.1016/j.biopha.2020.110203,
Cross talk between neutrophils and the microbiota,	https://doi.org/10.1182/blood-2018-11-844555,
Food for thought: Making the case for food produced via regenerative agriculture in the battle against non-communicable chronic diseases (NCDs),	https://doi.org/10.1016/j.onehlt.2024.100734,
Nutritional and dietary strategy in the clinical care of inflammatory bowel disease,	https://doi.org/10.1016/j.jfma.2019.09.005,
The role of botanical triterpenoids and steroids in bile acid metabolism, transport, and signaling: Pharmacological and toxicological implications,	https://doi.org/10.1016/j.apsb.2024.04.027,
The Structure and Function of the Human Small Intestinal Microbiota: Current Understanding and Future Directions,	https://doi.org/10.1016/j.jcmgh.2019.07.006,
Beneficial effects of Bifidobacterium longum subsp. longum BB536 on human health: Modulation of gut microbiome as the principal action,	https://doi.org/10.1016/j.jff.2019.02.002,
The Microbiome as a Modifier of Neurodegenerative Disease Risk,	https://doi.org/10.1016/j.chom.2020.06.008,
Emerging roles of bile acids in mucosal immunity and inflammation,	https://doi.org/10.1038/s41385-019-0162-4,
Faecal microbial transplant,	https://doi.org/10.1016/j.abst.2024.02.001,
Early life microbiome influences on development of the mucosal innate immune system,	https://doi.org/10.1016/j.smim.2024.101885,
Bacterial supplementation in mitigation of radiation-induced gastrointestinal damage,	https://doi.org/10.1016/j.lfs.2024.122921,
Dietary and Microbial Determinants in Food Allergy,	https://doi.org/10.1016/j.immuni.2020.07.025,
The role of the gut microbiome in chronic liver disease: the clinical evidence revised,	https://doi.org/10.1016/j.jhepr.2019.04.004,
Non-SCFA microbial metabolites associated with fiber fermentation and host health,	https://doi.org/10.1016/j.tem.2024.06.009,
Cytokine Networks in the Pathophysiology of Inflammatory Bowel Disease,	https://doi.org/10.1016/j.immuni.2019.03.017,
Gut Mycobiota in Immunity and Inflammatory Disease,	https://doi.org/10.1016/j.immuni.2019.05.023,
Cannabidiol - Help and hype in targeting mucosal diseases,	https://doi.org/10.1016/j.jconrel.2023.11.010,
Oral microbiota: A new view of body health,	https://doi.org/10.1016/j.fshw.2018.12.001,
Role of enteric dysbiosis in the development of central obesity: A review,	https://doi.org/10.1016/j.sciaf.2024.e02204,
Overview on biotics development,	https://doi.org/10.1016/j.copbio.2024.103073,
Prebiotics, probiotics, synbiotics and postbiotics to adolescents in metabolic syndrome,	https://doi.org/10.1016/j.clnu.2024.04.032,
Microbes, metabolites, and the gutâ€“lung axis,	https://doi.org/10.1038/s41385-019-0160-6,
Biomimetic Gut Model Systems for Development of Targeted Microbial Solutions for Enhancing Warfighter Health and Performance,	https://doi.org/10.1128/msystems.00487-20,
Atopic dermatitis and food allergy: More than sensitization,	https://doi.org/10.1016/j.mucimm.2024.06.005,
Neuropeptides in gut-brain axis and their influence on host immunity and stress,	https://doi.org/10.1016/j.csbj.2020.02.018,

Organoids as regenerative medicine for inflammatory bowel disease,	https://doi.org/10.1016/j.isci.2024.110118,
The influence of prenatal and intrapartum antibiotics on intestinal microbiota colonisation in infants: A systematic review,	https://doi.org/10.1016/j.jinf.2020.05.002,
Unleashing the Potential of Oral Deliverable Nanomedicine in the Treatment of Inflammatory Bowel Disease,	https://doi.org/10.1016/j.jcmgh.2024.03.005,
Immune boosting functional foods and their mechanisms: A critical evaluation of probiotics and prebiotics,	https://doi.org/10.1016/j.biopha.2020.110625,
The role of obesity in inflammatory bowel disease,	https://doi.org/10.1016/j.bbadis.2018.10.020,
Tryptophan metabolism and piglet diarrhea: Where we stand and the challenges ahead,	https://doi.org/10.1016/j.aninu.2024.03.005,
New insights into intestinal phages,	https://doi.org/10.1038/s41385-019-0250-5,
Bile salt hydrolase: The complexity behind its mechanism in relation to lowering-cholesterol lactobacilli probiotics,	https://doi.org/10.1016/j.jff.2024.106357,
Inflammatory Bowel Disease and Atherosclerotic Cardiovascular Disease: JACC Review Topic of the Week,	https://doi.org/10.1016/j.jacc.2020.10.027,
Inflammation-induced cellular changes: Genetic mutations, oncogene impact, and novel glycoprotein biomarkers,	https://doi.org/10.1016/j.abst.2024.06.002,
Efficacy of Lactobacillus plantarum in prevention of inflammatory bowel disease,	https://doi.org/10.1016/j.toxrep.2018.02.007,
The role of host molecules in communication with the resident and pathogenic microbiota: A review,	https://doi.org/10.1016/j.medmic.2020.100005,
Functional roles of gut bacteria imbalance in cholangiopathies,	https://doi.org/10.1016/j.livres.2018.11.001,
Chronic Inflammatory Diseases: Are We Ready for Microbiota-based Dietary Intervention?,	https://doi.org/10.1016/j.jcmgh.2019.02.008,
Evolution of fecal microbiota transplantation in methodology and ethical issues,	https://doi.org/10.1016/j.coph.2019.04.004,
Effect of fecal microbiota transplantation on 8-week remission in patients with ulcerative colitis: A randomized clinical trial.	10.1001/jama.2018.20046
Ferroptosis in ulcerative colitis: Potential mechanisms and promising therapeutic targets,	https://doi.org/10.1016/j.biopha.2024.116722,
Intestinal microbiota and colorectal carcinoma: Implications for pathogenesis, diagnosis, and therapy,	https://doi.org/10.1016/j.ebiom.2019.09.050,
Epithelial metabolism as a rheostat for intestinal inflammation and malignancy,	https://doi.org/10.1016/j.tcb.2024.01.004,
Regional Diversity of the Gastrointestinal Microbiome,	https://doi.org/10.1016/j.chom.2019.08.011,
How bacterial pathogens of the gastrointestinal tract use the mucosal glyco-code to harness mucus and microbiota: New ways to study an ancient bag of tricks,	https://doi.org/10.1016/j.ijmm.2020.151392,
Fecal microbiota transplantation: Review and update,	https://doi.org/10.1016/j.jfma.2018.08.011,

A critical review on the relationship of herbal medicine, Akkermansia muciniphila, and human health,	https://doi.org/10.1016/j.biopha.2020.110352,
Association of gut dysbiosis with intestinal metabolites in response to antibiotic treatment,	https://doi.org/10.1016/j.humic.2018.11.004,
Current Knowledge on the Preparation and Benefits of Cruciferous Vegetables as Relates to In Vitro, In Vivo, and Clinical Models of Inflammatory Bowel Disease,	https://doi.org/10.1016/j.cdnut.2024.102160,
Immune cell-derived signals governing epithelial phenotypes in homeostasis and inflammation,	https://doi.org/10.1016/j.molmed.2024.01.001,
Oxygen battle in the gut: Hypoxia and hypoxia-inducible factors in metabolic and inflammatory responses in the intestine,	https://doi.org/10.1074/jbc.REV120.011188,
Fecal microbiota transplantation for Clostridium difficile infection in Taiwan: Establishment and implementation,	https://doi.org/10.1016/j.jmii.2019.08.009,
Autism-associated synaptic mutations impact the gut-brain axis in mice,	https://doi.org/10.1016/j.bbi.2020.05.072,
Electrochemical Gut-on-Chip systems as the next generation platforms for studying mucosal redox biology,	https://doi.org/10.1016/j.coelec.2024.101442,
Imprinting of the immune system by the microbiota early in life,	https://doi.org/10.1038/s41385-020-0257-y,
The Intestinal Microbiota in Colorectal Cancer,	https://doi.org/10.1016/j.ccell.2018.03.004,
Metabolic regulation of the Th17/Treg balance in inflammatory bowel disease,	https://doi.org/10.1016/j.phrs.2024.107184,
Considering the Immune System during Fecal Microbiota Transplantation for Clostridioides difficile Infection,	https://doi.org/10.1016/j.molmed.2020.01.009,
Norovirus encounters in the gut: multifaceted interactions and disease outcomes,	https://doi.org/10.1038/s41385-019-0199-4,
Oxygen and Metabolism: Digesting Determinants of Antibiotic Susceptibility in the Gut,	https://doi.org/10.1016/j.isci.2020.101875,
Trends in 3D models of inflammatory bowel disease,	https://doi.org/10.1016/j.bbadis.2024.167042,
Decoding the mosaic of inflammatory bowel disease: Illuminating insights with single-cell RNA technology,	https://doi.org/10.1016/j.csbj.2024.07.011,
The lower airway microbiome in paediatric health and chronic disease,	https://doi.org/10.1016/j.prrv.2024.02.001,
Volatomics in inflammatory bowel disease and irritable bowel syndrome,	https://doi.org/10.1016/j.ebiom.2020.102725,
Sexual dimorphism of cardiometabolic dysfunction: Gut microbiome in the play?,	https://doi.org/10.1016/j.molmet.2018.05.016,
Induction mechanisms of autophagy and endoplasmic reticulum stress in intestinal ischemia-reperfusion injury, inflammatory bowel disease, and colorectal cancer,	https://doi.org/10.1016/j.biopha.2023.115984,
Unveiling ferroptosis as a promising therapeutic avenue for colorectal cancer and colitis treatment,	https://doi.org/10.1016/j.apsb.2024.05.025,
The Human Gut Microbiome: From Association to Modulation,	https://doi.org/10.1016/j.cell.2018.02.044,
Farnesoid X Receptor Agonists: A Promising Therapeutic Strategy for Gastrointestinal Diseases,	https://doi.org/10.1016/j.gastha.2023.09.013,

Phytogenic feed additives as natural antibiotic alternatives in animal health and production: A review of the literature of the last decade,	https://doi.org/10.1016/j.aninu.2024.01.012,
Olea europaea L-derived secoiridoids: Beneficial health effects and potential therapeutic approaches,	https://doi.org/10.1016/j.pharmthera.2024.108595,
Multiple therapeutic targets in rare cholestatic liver diseases: Time to redefine treatment strategies,	https://doi.org/10.1016/j.aohep.2019.09.009,
Reproductive toxicity and related mechanisms of micro(nano)plastics in terrestrial mammals: Review of current evidence,	https://doi.org/10.1016/j.ecoenv.2024.116505,
Accessing Bioactive Natural Products from the Human Microbiome,	https://doi.org/10.1016/j.chom.2018.05.013,
Progress in the metabolic kinetics and health benefits of functional polysaccharides from plants, animals and microbes: A review,	https://doi.org/10.1016/j.carpta.2024.100526,
Aryl hydrocarbon receptor and intestinal immunity,	https://doi.org/10.1038/s41385-018-0019-2,
Molecular Antioxidant and Immunological Mechanisms of Phytogenics in the Mitigation of Aflatoxicosis in Poultry,	https://doi.org/10.1016/j.japr.2024.100457,
Exploring the potential of human intestinal organoids: Applications, challenges, and future directions,	https://doi.org/10.1016/j.lfs.2024.122875,
The potential therapeutic benefits of Huaier in digestive system cancer: Its chemical components, pharmacological applications and future direction,	https://doi.org/10.1016/j.jff.2024.106267,
Plant cell-made protein antigens for induction of Oral tolerance,	https://doi.org/10.1016/j.biotechadv.2019.06.012,
Dietary non-coding RNAs from plants: Fairy tale or treasure?,	https://doi.org/10.1016/j.ncrna.2019.02.002,
A review on current advancement in zebrafish models to study chronic inflammatory diseases and their therapeutic targets,	https://doi.org/10.1016/j.heliyon.2024.e31862,
Negative Effects of a High-Fat Diet on Intestinal Permeability: A Review,	https://doi.org/10.1093/advances/nmz061,
Microbial modulation of intestinal T helper cell responses and implications for disease and therapy,	https://doi.org/10.1038/s41385-020-00335-w,
Recent advances in cancer chemoprevention with phytochemicals,	https://doi.org/10.1016/j.jfda.2019.11.001,
Myeloid C-type lectin receptors in innate immune recognition,	https://doi.org/10.1016/j.immuni.2024.03.005,
Deconstructing Mechanisms of Diet-Microbiome-Immune Interactions,	https://doi.org/10.1016/j.immuni.2020.07.015,
Protein Glycosylation as a Diagnostic and Prognostic Marker of Chronic Inflammatory Gastrointestinal and Liver Diseases,	https://doi.org/10.1053/j.gastro.2019.08.060,
A comprehensive review on ginger-derived exosome-like nanoparticles as feasible therapeutic nano-agents against diseases,	https://doi.org/10.1039/d3ma00856h,
Interactions between taste receptors and the gastrointestinal microbiome in inflammatory bowel disease,	https://doi.org/10.1016/j.jnim.2019.100106,

Bile acid receptors and gastrointestinal functions,	https://doi.org/10.1016/j.livres.2019.01.001,
Paradoxical role of Breg-inducing cytokines in autoimmune diseases,	https://doi.org/10.1016/j.jtauto.2019.100011,
The current state of the art for biological therapies and new small molecules in inflammatory bowel disease,	https://doi.org/10.1038/s41385-018-0050-3,
The â€œCultureâ€ of Pain Control: A Review of Opioid-Induced Dysbiosis (OID) in Antinociceptive Tolerance,	https://doi.org/10.1016/j.jpain.2019.11.015,
Cholangiocytes in the pathogenesis of primary sclerosing cholangitis and development of cholangiocarcinoma,	https://doi.org/10.1016/j.bbadis.2017.08.020,
Finding intestinal fortitude: Integrating the microbiome into a holistic view of depression mechanisms, treatment, and resilience,	https://doi.org/10.1016/j.nbd.2019.104578,
Carnosic acid: an effective phenolic diterpenoid for prevention and management of cancers via targeting multiple signaling pathways,	https://doi.org/10.1016/j.phrs.2024.107288,
Clinical implications of nicotine as an antimicrobial agent and immune modulator,	https://doi.org/10.1016/j.biopha.2020.110404,
Beyond butyrate: microbial fiber metabolism supporting colonic epithelial homeostasis,	https://doi.org/10.1016/j.tim.2023.07.014,
Inflammation, Autoimmunity and Neurodegenerative Diseases, Therapeutics and Beyond,	https://doi.org/10.2174/1570159X22666231017141636,
Interactions between human microbiome, diet, enteric viruses and immune system: Novel insights from gnotobiotic pig research,	https://doi.org/10.1016/j.ddmod.2019.08.006,
Preventive and therapeutic effects of ginger on bowel disease: A review of clinical trials,	https://doi.org/10.1016/j.prmcm.2024.100457,
Benefaction of probiotics for human health: AÂ review,	https://doi.org/10.1016/j.jfda.2018.01.002,
Chronic Visceral Pain: New Peripheral Mechanistic Insights and Resulting Treatments,	https://doi.org/10.1053/j.gastro.2024.01.045,
Tight Junctions as Targets and Effectors of Mucosal Immune Homeostasis,	https://doi.org/10.1016/j.jcmgh.2020.04.001,
Microfluidic Organ-on-a-Chip Models of Human Intestine,	https://doi.org/10.1016/j.jcmgh.2017.12.010,
An overview on the cellular mechanisms of anthocyanins in maintaining intestinal integrity and function,	https://doi.org/10.1016/j.fitote.2024.105953,
Aldo-keto reductase 1B: Much learned, much more to do,	https://doi.org/10.1016/j.hlife.2023.12.002,
New insights in intestinal oxidative stress damage and the health intervention effects of nutrients: A review,	https://doi.org/10.1016/j.jff.2020.104248,
Bronchiectasis,	https://doi.org/10.1016/j.opresp.2024.100339,
Vitamins and fatty acids against chemotherapy-induced intestinal mucositis,	https://doi.org/10.1016/j.pharmthera.2024.108689,
Microbiome and intestinal pathophysiology in post-acute sequelae of COVID-19,	https://doi.org/10.1016/j.gendis.2023.03.034,

Proteomics and Metaproteomics Add Functional, Taxonomic and Biomass Dimensions to Modeling the Ecosystem at the Mucosal-luminal Interface,	https://doi.org/10.1074/mcp.R120.002051,
Microbiome and type 1 diabetes,	https://doi.org/10.1016/j.ebiom.2019.06.031,
Inflammatory bowel disease and its treatment in 2018: Global and Taiwanese status updates,	https://doi.org/10.1016/j.jfma.2018.07.005,
Fusobacterium nucleatum Contributes to the Carcinogenesis of Colorectal Cancer by Inducing Inflammation and Suppressing Host Immunity,	https://doi.org/10.1016/j.tranon.2019.03.003,
Modulation of the Microbiome in Parkinson's Disease: Diet, Drug, Stool Transplant, and Beyond,	https://doi.org/10.1007/s13311-020-00942-2,
Microbiome-based stratification to guide dietary interventions to improve human health,	https://doi.org/10.1016/j.nutres.2020.07.004,
Probiotic, prebiotic, synbiotic and fermented food supplementation in psychiatric disorders: A systematic review of clinical trials,	https://doi.org/10.1016/j.neubiorev.2024.105561,
The social microbiome: The missing mechanism mediating the sociality-fitness nexus?,	https://doi.org/10.1016/j.isci.2024.109806,
Exploring the HLA complex in autoimmunity: From the risk haplotypes to the modulation of expression,	https://doi.org/10.1016/j.clim.2024.110266,
Nature versus nurture in the spectrum of rheumatic diseases: Classification of spondyloarthritis as autoimmune or autoinflammatory,	https://doi.org/10.1016/j.autrev.2018.04.002,
Proanthocyanidins in grape seeds: An updated review of their health benefits and potential uses in the food industry,	https://doi.org/10.1016/j.jff.2020.103861,
Î±-Ketoglutarate for Preventing and Managing Intestinal Epithelial Dysfunction,	https://doi.org/10.1016/j.advnut.2024.100200,
Site-specific targeted drug delivery systems for the treatment of inflammatory bowel disease,	https://doi.org/10.1016/j.biopha.2020.110486,
The role of Akkermansia muciniphila in colorectal cancer: A double-edged sword of treatment or disease progression?,	https://doi.org/10.1016/j.biopha.2024.116416,
Probiotic characteristics of Bacillus coagulans and associated implications for human health and diseases,	https://doi.org/10.1016/j.jff.2019.103643,
An unmet need for pharmacology: Treatments for radiation-induced gastrointestinal mucositis,	https://doi.org/10.1016/j.biopha.2024.116767,
Targeting aryl hydrocarbon receptor to prevent cancer in barrier organs,	https://doi.org/10.1016/j.bcp.2024.116156,
Who will carry out the tests that would be necessary for proper safety evaluation of food emulsifiers?,	https://doi.org/10.1016/j.fshw.2019.04.001,
Therapeutic potential of traditional Chinese medicine in the prevention and treatment of digestive inflammatory cancer transformation: Portulaca oleracea L. as a promising drug,	https://doi.org/10.1016/j.jep.2024.117999,
The internationalization of human microbiome research,	https://doi.org/10.1016/j.mib.2019.09.012,
Next generation probiotics in disease amelioration,	https://doi.org/10.1016/j.jfda.2018.12.011,
Gut reaction: impact of systemic diseases on gastrointestinal physiology and drug absorption,	https://doi.org/10.1016/j.drudis.2018.11.009,

Mechanisms of gastrointestinal microflora on drug metabolism in clinical practice,	https://doi.org/10.1016/j.jsps.2019.09.011,
The potential impact of nano- and microplastics on human health: Understanding human health risks.,	https://doi.org/10.1016/j.envres.2024.118535,
Unveiling the potential of estrogen: Exploring its role in neuropsychiatric disorders and exercise intervention,	https://doi.org/10.1016/j.phrs.2024.107201,
The path toward using microbial metabolites as therapies,	https://doi.org/10.1016/j.ebiom.2019.05.063,
Hematopoiesis and the bacterial microbiome,	https://doi.org/10.1182/blood-2018-02-832519,
The interplay between innate lymphoid cells and T cells,	https://doi.org/10.1038/s41385-020-0320-8,
The Role of Adaptor Protein CARD9 in Colitis-Associated Cancer,	https://doi.org/10.1016/j.omto.2019.08.007,
Retinoid-Related Orphan Receptor RORÎ³t in CD4+ T-Cellâ€"Mediated Intestinal Homeostasis and Inflammation,	https://doi.org/10.1016/j.ajpath.2020.07.010,
ASBT(SLC10A2): A promising target for treatment of diseases and drug discovery,	https://doi.org/10.1016/j.biopha.2020.110835,
Oral hygiene might prevent cancer,	https://doi.org/10.1016/j.heliyon.2018.e00879,
Review of Dermatologic Drugs Relevant to Inflammatory Bowel Disease,	https://doi.org/10.1016/j.jdrv.2024.07.001,
Exploring the synergy of artificial intelligence in microbiology: Advancements, challenges, and future prospects,	https://doi.org/10.1016/j.csbr.2024.100005,
Animal models of cholestasis: An update on inflammatory cholangiopathies,	https://doi.org/10.1016/j.bbadis.2018.07.025,
A cross comparison between Ayurvedic etiology of Major Depressive Disorder and bidirectional effect of gut dysregulation,	https://doi.org/10.1016/j.jaim.2017.08.002,
Utilizing microbiome approaches to assist source tracking, treatment and prevention of COVID-19: Review and assessment,	https://doi.org/10.1016/j.csbj.2020.11.027,
The power of small changes: Comprehensive analyses of microbial dysbiosis in breast cancer,	https://doi.org/10.1016/j.bbcan.2019.04.001,
T cell immunity to commensal fungi,	https://doi.org/10.1016/j.mib.2020.09.008,
Enteropathic spondyloarthritis: Results from a large nationwide database analysis,	https://doi.org/10.1016/j.autrev.2019.102457,
Systematic review and meta-analysis of habitual intake of fermentable oligo-, di-, mono-saccharides and polyols in the general population and revisiting the low FODMAP diet concept,	https://doi.org/10.1016/j.jff.2023.105914,
A new era in healthcare: The integration of artificial intelligence and microbial,	https://doi.org/10.1016/j.medntd.2024.100319,
Enteric Glia: A New Player in Abdominal Pain,	https://doi.org/10.1016/j.jcmgh.2018.11.005,
The Cancer Microbiome: Distinguishing Direct and Indirect Effects Requires a Systemic View,	https://doi.org/10.1016/j.trecan.2020.01.004,
Establishment and evaluation of on-chip intestinal barrier biosystems based on microfluidic techniques,	https://doi.org/10.1016/j.mtbio.2024.101079,
Modulation of inflammatory response and pain by mind-body therapies as meditation,	https://doi.org/10.1016/j.bbii.2023.100036,

Updates in the pathogenesis and management of immune-related enterocolitis, hepatitis and cardiovascular toxicities,	https://doi.org/10.1016/j.iotech.2024.100704,
The interplay between cytokines, inflammation, and antioxidants: mechanistic insights and therapeutic potentials of various antioxidants and anti-cytokine compounds,	https://doi.org/10.1016/j.biopha.2024.117177,
Painful interactions: Microbial compounds and visceral pain,	https://doi.org/10.1016/j.bbadis.2019.165534,
Efficacy of diets with specific compositions to reduce the symptoms of immune-mediated diseases. Narrative review,	https://doi.org/10.1016/j.phanu.2024.100394,
Local complement activation and modulation in mucosal immunity,	https://doi.org/10.1016/j.mucimm.2024.05.006,
Can molecular stratification improve the treatment of inflammatory bowel disease?,	https://doi.org/10.1016/j.phrs.2019.104442,
The impact of a helminth-modified microbiome on host immunity,	https://doi.org/10.1038/s41385-018-0008-5,
Post-Acute Sequelae of Covid-19: A System-wise Approach on the Effects of Long-Covid-19,	https://doi.org/10.1016/j.ajmo.2024.100071,
Network modeling approaches for metabolic diseases and diabetes,	https://doi.org/10.1016/j.coisb.2024.100530,
â€˜RA and the microbiome: do host genetic factors provide the link?,	https://doi.org/10.1016/j.jaut.2019.02.004,
The effects of NOD-like receptors on adaptive immune responses,	https://doi.org/10.1016/j.bj.2023.100637,
The role of m6A RNA methylation in autoimmune diseases: Novel therapeutic opportunities,	https://doi.org/10.1016/j.gendis.2023.02.013,
T Cell Antifungal Immunity and the Role of C-Type Lectin Receptors,	https://doi.org/10.1016/j.it.2019.11.007,
Neuro-immune Interactions in the Tissues,	https://doi.org/10.1016/j.immuni.2020.02.017,
Molecular mechanisms underpinning T helper 17 cell heterogeneity and functions in rheumatoid arthritis,	https://doi.org/10.1016/j.jaut.2017.12.006,
Emerging trends and future challenges of advanced 2D nanomaterials for combating bacterial resistance,	https://doi.org/10.1016/j.bioactmat.2024.04.033,
New and alternative strategies for the prevention, control, and treatment of antibiotic-resistant Campylobacter,	https://doi.org/10.1016/j.trsl.2020.04.009,
Efficacy of tofacitinib treatment in ulcerative colitis,	https://doi.org/10.1016/j.gastre.2019.03.012,
Pathophysiologic implications of innate immunity and autoinflammation in the biliary epithelium,	https://doi.org/10.1016/j.bbadis.2017.07.023,
Plant-derived nanovesicles as an emerging platform for cancer therapy,	https://doi.org/10.1016/j.apsb.2023.08.033,
Amino acid metabolism as drug target in autoimmune diseases,	https://doi.org/10.1016/j.autrev.2019.02.004,
Epithelialâ€"mesenchymal transition in Crohn's disease,	https://doi.org/10.1038/mi.2017.107,
Adalimumab therapy improves intestinal dysbiosis in Crohn's disease	https://doi.org/10.3390/jcm8101646
Navigating the Interplay between BCL-2 Family Proteins, Apoptosis, and Autophagy in Colorectal Cancer,	https://doi.org/10.1016/j.adcanc.2024.100126,
Dichotomous effects of microbial membrane vesicles on the regulation of immunity,	https://doi.org/10.1016/j.medmic.2020.100009,

Cell-by-Cell Deconstruction of Stem Cell Niches,	https://doi.org/10.1016/j.stem.2020.06.013,
Autophagy in regulatory T cells: A double-edged sword in disease settings,	https://doi.org/10.1016/j.molimm.2019.02.004,
New insights into nucleic acid sensor AIM2: The potential benefit in targeted therapy for cancer,	https://doi.org/10.1016/j.phrs.2024.107079,
Regulation of the Immune Response by the Aryl Hydrocarbon Receptor,	https://doi.org/10.1016/j.immuni.2017.12.012,
Autonomic dysfunction in Parkinsonâ€™s disease: Implications for pathophysiology, diagnosis, and treatment,	https://doi.org/10.1016/j.nbd.2019.104700,
Tipping the balance: inhibitory checkpoints in intestinal homeostasis,	https://doi.org/10.1038/s41385-018-0113-5,
Innate immune signal transduction pathways to fungal infection: Components and regulation,	https://doi.org/10.1016/j.cellin.2024.100154,
Cecropins in cancer therapies-where we have been?,	https://doi.org/10.1016/j.ejphar.2020.173317,
Bacillus coagulans as a potent intervention for treating irritable bowel syndrome: A systematic review and meta-analysis of randomized control trials,	https://doi.org/10.1016/j.gande.2023.11.001,
A comprehensive review on potential role of selenium, selenoproteins and selenium nanoparticles in male fertility,	https://doi.org/10.1016/j.heliyon.2024.e34975,
Urban-associated diseases: Candidate diseases, environmental risk factors, and a path forward,	https://doi.org/10.1016/j.envint.2019.105187,
Research status and challenges of plant-derived exosome-like nanoparticles,	https://doi.org/10.1016/j.biopha.2024.116543,
Nano pharmaceutical delivery in combating colorectal cancer,	https://doi.org/10.1016/j.medidd.2023.100173,
Metabolomics in the era of artificial intelligence,	https://doi.org/10.1530/MAH-23-0017,
Biomaterial-enhanced treg cell immunotherapy: A promising approach for transplant medicine and autoimmune disease treatment,	https://doi.org/10.1016/j.bioactmat.2024.03.030,
Organoids â€“ New Models for Hostâ€“Helminth Interactions,	https://doi.org/10.1016/j.pt.2019.10.013,
Machine learning application in autoimmune diseases: State of art and future prospectives,	https://doi.org/10.1016/j.autrev.2023.103496,
Lupus and other autoimmune diseases: epidemiology in the population of African ancestry, and diagnostic and management challenges in Africa,	https://doi.org/10.1016/j.jacig.2024.100288,
Keratin intermediate filaments in the colon: guardians of epithelial homeostasis,	https://doi.org/10.1016/j.biocel.2020.105878,
Western Diet and the Immune System: An Inflammatory Connection,	https://doi.org/10.1016/j.immuni.2019.09.020,
TH17â€¯cell plasticity: The role of dendritic cells and molecular mechanisms,	https://doi.org/10.1016/j.jaut.2017.12.003,
The clinical features and potential mechanisms of cognitive disorders in peripheral autoimmune and inflammatory diseases,	https://doi.org/10.1016/j.fmre.2022.12.005,
IL-10 Family Cytokines IL-10 and IL-22: from Basic Science to Clinical Translation,	https://doi.org/10.1016/j.immuni.2019.03.020,
Interleukin-1 and Related Cytokines in the Regulation of Inflammation and Immunity,	https://doi.org/10.1016/j.immuni.2019.03.012,
Curcumin as a regulator of Th17 cells: Unveiling the mechanisms,	https://doi.org/10.1016/j.fochms.2024.100198,

Interaction between high-density lipoproteins and inflammation: Function matters more than concentration!,	https://doi.org/10.1016/j.addr.2020.10.006,
Fusobacterium nucleatum, the communication with colorectal cancer,	https://doi.org/10.1016/j.biopha.2019.108988,
Cosmic chronometers: Is spaceflight a catalyst for biological ageing?,	https://doi.org/10.1016/j.arr.2024.102227,
Highlighting Clinical Metagenomics for Enhanced Diagnostic Decision-making: A Step Towards Wider Implementation,	https://doi.org/10.1016/j.csbj.2018.02.006,
Mechanisms of activation of innate-like intraepithelial T lymphocytes,	https://doi.org/10.1038/s41385-020-0294-6,
Pediatric Inflammatory Bowel Disease in Asia: Epidemiology and natural history,	https://doi.org/10.1016/j.pedneo.2019.12.008,
Immunomodulatory bioactivities of glycomacropeptide,	https://doi.org/10.1016/j.jff.2024.106084,
The Lymphatic Vasculature in the 21st Century: Novel Functional Roles in Homeostasis and Disease,	https://doi.org/10.1016/j.cell.2020.06.039,
Health-promoting compounds in Amomum villosum Lour and Amomum tsao-ko: Fruit essential oil exhibiting great potential for human health,	https://doi.org/10.1016/j.heliyon.2024.e27492,
Food protein-induced allergic proctocolitis in infants: Literature review and proposal of a management protocol,	https://doi.org/10.1016/j.waojou.2020.100471,
Two to Tango: Dialog between Immunity and Stem Cells in Health and Disease,	https://doi.org/10.1016/j.cell.2018.08.071,
The IL-17 Family of Cytokines in Health and Disease,	https://doi.org/10.1016/j.immuni.2019.03.021,
Pathological consequences, metabolism and toxic effects of trichothecene T-2 toxin in poultry,	https://doi.org/10.1016/j.psj.2024.103471,
The Pediatric Cell Atlas: Defining the Growth Phase of Human Development at Single-Cell Resolution,	https://doi.org/10.1016/j.devcel.2019.03.001,
Hydrogen sulfide: An endogenous regulator of the immune system,	https://doi.org/10.1016/j.phrs.2020.105119,
Oral administration microrobots for drug delivery,	https://doi.org/10.1016/j.bioactmat.2024.05.005,
Inflammatory consequences of inherited disorders affecting neutrophil function,	https://doi.org/10.1182/blood-2018-11-844563,
The Biology of T Regulatory Type 1 Cells and Their Therapeutic Application in Immune-Mediated Diseases,	https://doi.org/10.1016/j.immuni.2018.12.001,
Innovative cellular therapies for autoimmune diseases: expert-based position statement and clinical practice recommendations from the EBMT practice harmonization and guidelines committee,	https://doi.org/10.1016/j.eclinm.2024.102476,
Towards the development of human immune-system-on-a-chip platforms,	https://doi.org/10.1016/j.drudis.2018.10.003,
Recent advances in carbon monoxide-releasing nanomaterials,	https://doi.org/10.1016/j.bioactmat.2024.03.001,
Backyard poultry: exploring non-intensive production systems,	https://doi.org/10.1016/j.psj.2023.103284,
Challenges, Progress, and Prospects of Developing Therapies to Treat Autoimmune Diseases,	https://doi.org/10.1016/j.cell.2020.03.007,

Molecular insights into experimental models and therapeutics for cholestasis,	https://doi.org/10.1016/j.biopha.2024.116594,
Therapeutic potential of mesenchymal stem cells and its exosomes in colorectal cancer: Paving way from preclinical towards clinical road,	https://doi.org/10.1016/j.adcanc.2024.100123,
Immune dysregulation in patients with RAG deficiency and other forms of combined immune deficiency,	https://doi.org/10.1182/blood.2019000923,
Painful interactions: Microbial compounds and visceral pain,	https://doi.org/10.1016/j.bbadis.2019.165534,
Systemic autoinflammatory diseases,	https://doi.org/10.1016/j.jaut.2020.102421,
COPD phenotypes and machine learning cluster analysis: A systematic review and future research agenda,	https://doi.org/10.1016/j.rmed.2020.106093,
Preoperative enteral nutrition in adults with complicated Crohn's disease: Effect on disease outcomes and gut microbiota,	https://doi.org/10.1016/j.nutx.2020.100009,
The Appendix Orchestrates T-Cell Mediated Immunosurveillance in Colitis-Associated Cancer,	https://doi.org/10.1016/j.jcmgh.2022.10.016,
Grape peel powder attenuates the inflammatory and oxidative response of experimental colitis in rats by modulating the NF-ÃŽÂºB pathway and activity of antioxidant enzymes,	https://doi.org/10.1016/j.nutres.2020.01.006,
Excess Dietary Sugar Alters Colonocyte Metabolism and Impairs the Proliferative Response to Damage,	https://doi.org/10.1016/j.jcmgh.2023.05.001,
Microbiota-derived short chain fatty acids: Their role and mechanisms in viral infections,	https://doi.org/10.1016/j.biopha.2023.114414,
Role of MR enterography in acute and chronic stages of Crohn's disease,	https://doi.org/10.1016/j.ejrnm.2018.05.012,
Gene polymorphism in IL17A and gene-gene interaction in the IL23R/IL17A axis are associated with susceptibility to coronary artery disease,	https://doi.org/10.1016/j.cyto.2023.156142,
Global incidence and prevalence of autoimmune hepatitis, 1970Ã¢â‚¬â€œ2022: a systematic review and meta-analysis,	https://doi.org/10.1016/j.eclinm.2023.102280,
Sexual dimorphism of cardiometabolic dysfunction: Gut microbiome in the play?,	https://doi.org/10.1016/j.molmet.2018.05.016,
Anthocleista vogelii (Planch) stem bark ethanol extract prevents inflammatory and pain phenotypes in experimental rodent models by modulating oxido-inflammatory mediators,	https://doi.org/10.1016/j.phyplu.2023.100475,
16S full-length gene sequencing analysis of intestinal flora in breast cancer patients in Hainan Province,	https://doi.org/10.1016/j.mcp.2023.101927,
The complex role of inflammation and gliotransmitters in Parkinson's disease,	https://doi.org/10.1016/j.nbd.2022.105940,
**SCIENCEDIRECT - IBD AND BIOLOGICAL THERAPIES (435 RESULTS)**	
Biologics and the timing of operative management of pediatric inflammatory bowel disease,	https://doi.org/10.1016/j.yjpso.2023.100084,

React, reset and restore: Adaptation of a large inflammatory bowel disease service during COVID-19 pandemic,	https://doi.org/10.7861/clinmed.2020-0369,
Therapeutic targets for inflammatory bowel disease: proteome-wide Mendelian randomization and colocalization analyses,	https://doi.org/10.1016/j.ebiom.2023.104494,
A Cross-Sectional Survey on the Transitional Care of Adolescents with Inflammatory Bowel Disease in Hungary,	https://doi.org/10.1016/j.pedn.2020.06.002,
The single-cell transcriptional landscape of innate and adaptive lymphocytes in pediatric-onset colitis,	https://doi.org/10.1016/j.xcrm.2023.101038,
Oxidative Stress and Redox-Modulating Therapeutics in Inflammatory Bowel Disease,	https://doi.org/10.1016/j.molmed.2020.06.006,
Inflammatory Bowel Diseases Before and After 1990,	https://doi.org/10.1016/j.gastha.2022.08.001,
Neutralising antibody responses against SARS-CoV-2 Omicron BA.4/5 and wild-type virus in patients with inflammatory bowel disease following three doses of COVID-19 vaccine (VIP): a prospective, multicentre, cohort study,	https://doi.org/10.1016/j.eclinm.2023.102249,
m6A modification in inflammatory bowel disease provides new insights into clinical applications,	https://doi.org/10.1016/j.biopha.2023.114298,
Assessment of anti-inflammatory efficacy of acupuncture in patients with inflammatory bowel disease: A systematic review and meta-analysis,	https://doi.org/10.1016/j.ctim.2023.102946,
Gum-gut axis: The potential role of salivary biomarkers in the diagnosis and monitoring progress of inflammatory bowel diseases,	https://doi.org/10.1016/j.sdentj.2022.12.006,
Relationship between clinical features and intestinal microbiota in Chinese patients with ulcerative colitis.	https://doi.org/10.3748/wjg.v27.i28.4722
Platelet-Rich stroma from CrohnÃ¢â‚¬â„¢s disease patients for treatment of perianal fistula shows a higher myeloid cell profile compared to non-IBD controls,	https://doi.org/10.1016/j.scr.2023.103039,
A retrospective analysis of treatment patterns, drug discontinuation and healthcare costs in Crohn's disease patients treated with biologics,	https://doi.org/10.1016/j.dld.2023.04.010,
Management of inflammatory bowel disease in the elderly: A review,	https://doi.org/10.1016/j.dld.2022.12.024,
Expression Levels of 4 Genes in Colon Tissue Might Be Used to Predict Which Patients Will Enter Endoscopic Remission After Vedolizumab Therapy for Inflammatory Bowel Diseases,	https://doi.org/10.1016/j.cgh.2019.08.030,
COVID-19 and pediatric inflammatory bowel disease: How to manage it?,	https://doi.org/10.6061/clinics/2020/e1962,
Correlation of anti-TNF-a biological therapy with periodontal conditions and osteonecrosis in autoimmune patients: A systematic review,	https://doi.org/10.1016/j.sdentj.2023.07.006,
Adipokine C1q/Tumor Necrosis Factor- Related Protein 3 (CTRP3) Attenuates Intestinal Inflammation Via Sirtuin 1/NF-ÃŽÂºB Signaling,	https://doi.org/10.1016/j.jcmgh.2022.12.013,

Exploring the efficacy of herbal medicinal products as oral therapy for inflammatory bowel disease,	https://doi.org/10.1016/j.biopha.2023.115266,
ESCMID Study Group for Infections in Compromised Hosts (ESGICH) Consensus Document on the safety of targeted and biological therapies: an infectious diseases perspective (Soluble immune effector molecules [I]: anti-tumor necrosis factor-ÃŽÂ± agents),	https://doi.org/10.1016/j.cmi.2017.12.025,
Varicella-zoster-virus vaccination of immunosuppressed children with inflammatory bowel disease or autoimmune hepatitis: A prospective observational study,	https://doi.org/10.1016/j.vaccine.2020.10.028,
Biologics in peripheral ulcerative keratitis,	https://doi.org/10.1016/j.semarthrit.2023.152269,
Breaking through the therapeutic ceiling of inflammatory bowel disease: Dual-targeted therapies,	https://doi.org/10.1016/j.biopha.2022.114174,
DNA methylation of the TPMT gene and azathioprine pharmacokinetics in children with very early onset inflammatory bowel disease,	https://doi.org/10.1016/j.biopha.2022.113901,
Possible role of nutrition in the prevention of inflammatory bowel diseaseÃ¢â‚¬â€œrelated colorectal cancer: A focus on human studies,	https://doi.org/10.1016/j.nut.2023.111980,
Final Analysis of COVID-19 Patients With Inflammatory Bowel Disease in Japan (J-COSMOS): A Multicenter Registry Cohort Study,	https://doi.org/10.1016/j.gastha.2023.07.017,
A real-world, long-term experience on effectiveness and safety of vedolizumab in adult patients with inflammatory bowel disease: The Cross Pennine study,	https://doi.org/10.1016/j.dld.2018.07.007,
Efficacy and safety of methotrexate in the management of inflammatory bowel disease: A systematic review and meta-analysis of randomized, controlled trials,	https://doi.org/10.1016/j.eclinm.2020.100271,
Performance of fecal S100A12 as a novel non-invasive diagnostic biomarker for pediatric inflammatory bowel disease: a systematic review and meta-analysis,	https://doi.org/10.1016/j.jped.2023.03.002,
The Effect of In Utero Exposure to Maternal Inflammatory Bowel Disease and Immunomodulators on Infant Immune System Development and Function,	https://doi.org/10.1016/j.jcmgh.2023.03.005,
Systematic review: Sarcopenia in paediatric inflammatory bowel disease,	https://doi.org/10.1016/j.clnesp.2023.08.009,
Prognostic modelling in IBD,	https://doi.org/10.1016/j.bpg.2023.101877,
Gut vascular barrier in the pathogenesis and resolution of Crohn's disease: A novel link from origination to therapy,	https://doi.org/10.1016/j.clim.2023.109683,
A descriptive study of inflammatory bowel disease at an Egyptian tertiary care center,	https://doi.org/10.1016/j.rgmxen.2021.11.015,
Statin use and risk of colorectal cancer in patients with inflammatory bowel disease,	https://doi.org/10.1016/j.eclinm.2023.102182,
Fecal microbiota transplantation for recurrent C. difficile infection in patients with inflammatory bowel disease: A systematic review and meta-analysis,	https://doi.org/10.1016/j.jaut.2023.103036,
Complete Endoscopic Healing Is Associated With Lower Relapse Risk After Anti-TNF Withdrawal in Inflammatory Bowel Disease,	https://doi.org/10.1016/j.cgh.2022.08.024,

Myocarditis and inflammatory bowel diseases: A single-center experience and a systematic literature review,	https://doi.org/10.1016/j.ijcard.2023.01.071,
Pulmonary Manifestations of Inflammatory Bowel Disease and Treatment Strategies,	https://doi.org/10.1016/j.chpulm.2023.100018,
Characterizing the pre-clinical phase of inflammatory bowel disease,	https://doi.org/10.1016/j.xcrm.2023.101263,
Patients with inflammatory bowel disease are at increased risk of atherothrombotic disease: A systematic review with meta-analysis,	https://doi.org/10.1016/j.ijcard.2023.02.042,
Longitudinal Fecal Calprotectin Profiles Characterize Disease Course Heterogeneity in CrohnÃ¢â‚¬â„¢s Disease,	https://doi.org/10.1016/j.cgh.2023.03.026,
Negative regulatory NLRs mitigate inflammation via NF-ÃŽÂºB pathway signaling in inflammatory bowel disease,	https://doi.org/10.1016/j.bj.2023.100616,
Long-term Temporal Stability of Peripheral Blood DNA Methylation Profiles in Patients With Inflammatory Bowel Disease,	https://doi.org/10.1016/j.jcmgh.2022.12.011,
Immune-mediated inflammatory diseases: Common and different pathogenic and clinical features,	https://doi.org/10.1016/j.autrev.2023.103410,
Defining Interactions Between the Genome, Epigenome, and the Environment in Inflammatory Bowel Disease: Progress and Prospects,	https://doi.org/10.1053/j.gastro.2023.03.238,
Global Hospitalization Trends for CrohnÃ¢â‚¬â„¢s Disease and Ulcerative Colitis in the 21st Century: A Systematic Review With Temporal Analyses,	https://doi.org/10.1016/j.cgh.2022.06.030,
Daily, oral FMT for long-term maintenance therapy in ulcerative colitis: results of a single-center, prospective, randomized pilot study	https://doi.org/10.1186/s12876-021-01856-9
Infectious Mimics of Inflammatory Bowel Disease,	https://doi.org/10.1016/j.modpat.2023.100210,
Microbiome-phage interactions in inflammatory bowel disease,	https://doi.org/10.1016/j.cmi.2022.08.027,
Neutralising antibody potency against SARS-CoV-2 wild-type and omicron BA.1 and BA.4/5 variants in patients with inflammatory bowel disease treated with infliximab and vedolizumab after three doses of COVID-19 vaccine (CLARITY IBD): an analysis of a prospective multicentre cohort study,	https://doi.org/10.1016/S2468-1253(22)00389-2,
Organoid-based regenerative medicine for inflammatory bowel disease,	https://doi.org/10.1016/j.reth.2019.11.004,
A novel rat model of inflammatory bowel disease developed using a device created with a 3D printer,	https://doi.org/10.1016/j.reth.2019.12.005,
Lower magnitude and faster waning of antibody responses to SARS-CoV-2 vaccination in anti-TNF-ÃŽÂ±-treated IBD patients are linked to lack of activation and expansion of cTfh1 cells and impaired B memory cell formation,	https://doi.org/10.1016/j.ebiom.2023.104788,
Rate of Adverse Events and Associated Health Care Costs for the Management of Inflammatory Bowel Disease in Germany,	https://doi.org/10.1016/j.clinthera.2019.11.012,

Methodology, efficacy and safety of fecal microbiota transplantation in treating inflammatory bowel disease,	https://doi.org/10.1016/j.medmic.2020.100028,
Prevalence and Clinical Features of Inflammatory Bowel Diseases Associated With Monogenic Variants, Identified by Whole-Exome Sequencing in 1000 Children at a Single Center,	https://doi.org/10.1053/j.gastro.2020.02.023,
Outcomes of Infliximab-Treated inflammatory bowel disease patients undergoing therapeutic drug monitoring with two different assays,	https://doi.org/10.1016/j.clinbiochem.2023.110618,
Longitudinal trajectories of anxiety, depression, and bipolar disorder in inflammatory bowel disease: a population-based cohort study,	https://doi.org/10.1016/j.eclinm.2023.101986,
Unfermented ÃŽÂ²-fructan Fibers Fuel Inflammation in Select Inflammatory Bowel Disease Patients,	https://doi.org/10.1053/j.gastro.2022.09.034,
Commensal bacteria promote azathioprine therapy failure in inflammatory bowel disease via decreasing 6-mercaptopurine bioavailability,	https://doi.org/10.1016/j.xcrm.2023.101153,
Therapeutic effects of combination of platelet lysate and sulfasalazine administration in TNBS-induced colitis in rat,	https://doi.org/10.1016/j.biopha.2020.109949,
Site-specific targeted drug delivery systems for the treatment of inflammatory bowel disease,	https://doi.org/10.1016/j.biopha.2020.110486,
Inflammatory Bowel Disease and Atherosclerotic Cardiovascular Disease: JACC Review Topic of the Week,	https://doi.org/10.1016/j.jacc.2020.10.027,
Risk of SARS-CoV-2 infection in healthcare workers with inflammatory bowel disease: a case-control study,	https://doi.org/10.1016/j.infpip.2022.100267,
Intestinal microbiome and metabolome signatures in patients with chronic granulomatous disease,	https://doi.org/10.1016/j.jaci.2023.07.022,
The Impact of Incarceration on Readmissions Among Patients With Inflammatory Bowel Disease Hospitalized at a Community Hospital,	https://doi.org/10.1016/j.gastha.2023.03.016,
Increasing Risk of Lymphoma Over Time in CrohnÃ¢â‚¬â„¢s DiseaseÃ‚Â but Not in Ulcerative Colitis: A Scandinavian CohortÃ‚Â Study,	https://doi.org/10.1016/j.cgh.2023.04.001,
Gene expression profiling in white blood cells reveals new insights into the molecular mechanisms of thalidomide in children with inflammatory bowel disease,	https://doi.org/10.1016/j.biopha.2023.114927,
Phase angle values and ultra-processed food consumption are associated with changes in oxidative stress in inflammatory bowel disease patients,	https://doi.org/10.1016/j.clnesp.2023.06.006,
Impact of the COVID-19 pandemic on inflammatory bowel disease care in Taiwan: A multicenter study,	https://doi.org/10.1016/j.jfma.2023.03.017,
Fecal microbiota transplantation for recurrent Clostridioides difficile infection in patients with concurrent ulcerative colitis,	https://doi.org/10.1016/j.jaut.2023.103033,

Cell free bacterial DNAs in human plasma provide fingerprints for immune-related diseases,	https://doi.org/10.1016/j.medmic.2020.100022,
The gut microbes in inflammatory bowel disease: Future novel target option for pharmacotherapy,	https://doi.org/10.1016/j.biopha.2023.114893,
Anti-tumor necrosis factor therapy is associated with attenuated humoral response to SARS-COV-2 vaccines in patients with inflammatory bowel disease,	https://doi.org/10.1016/j.vaccine.2023.05.012,
Natural products targeting Nrf2/ARE signaling pathway in the treatment of inflammatory bowel disease,	https://doi.org/10.1016/j.biopha.2023.114950,
Local fistula injection of allogeneic human amnion epithelial cells is safe and well tolerated in patients with refractory complex perianal Crohn's disease: a phase I open label study with long-term follow up,	https://doi.org/10.1016/j.ebiom.2023.104879,
Budesonide, an anti-inflammatory drug, exacerbate clostridioides difficile colitis in mice,	https://doi.org/10.1016/j.biopha.2023.115489,
Progression of Inflammatory Bowel Diseases Throughout Latin America and the Caribbean: A Systematic Review,	https://doi.org/10.1016/j.cgh.2019.06.030,
Vitamin D and Microbiome: Molecular Interaction in Inflammatory Bowel Disease Pathogenesis,	https://doi.org/10.1016/j.ajpath.2023.02.004,
Aspects of inflammatory bowel disease during the COVID-19 pandemic and general considerations,	https://doi.org/10.1016/j.rgmxen.2020.05.001,
Corticosteroids in inflammatory bowel disease: Are they still a therapeutic option?,	https://doi.org/10.1016/j.gastre.2022.10.019,
Phage therapy: Targeting intestinal bacterial microbiota for the treatment of liver diseases,	https://doi.org/10.1016/j.jhepr.2023.100909,
Wumei Wan attenuates angiogenesis and inflammation by modulating RAGE signaling pathway in IBD: Network pharmacology analysis and experimental evidence,	https://doi.org/10.1016/j.phymed.2023.154658,
Incidence of suboptimal response to tumor necrosis factor antagonist therapy in inflammatory bowel disease in newly industrialised countries: The EXPLORE study,	https://doi.org/10.1016/j.dld.2020.05.031,
Conception and reality: Outcome of SARS-CoV-2 infection and vaccination among Hungarian IBD patients on biologic treatments,	https://doi.org/10.1016/j.jvacx.2022.100253,
Ã¢â‚¬Å“It's just like putting your socks onÃ¢â‚¬Â : patients' perspectives on inflammatory bowel disease medication adherence,	https://doi.org/10.1016/j.rcsop.2023.100385,
Tezacaftor/Ivacaftor therapy has negligible effects on the cystic fibrosis gut microbiome,	https://doi.org/10.1128/spectrum.01175-23,
The effectiveness of mindfulness-based interventions in inflammatory bowel disease: A Systematic Review & Meta-Analysis,	https://doi.org/10.1016/j.jpsychores.2023.111232,
Using insurance claims to predict and improve hospitalizations and biologics use in members with inflammatory bowel diseases,	https://doi.org/10.1016/j.jbi.2018.03.015,
Disruption of IL-18 signaling via engineered IL-18BP biologics alleviates experimental cholestatic liver disease,	https://doi.org/10.1016/j.biopha.2023.115587,

Gut dysbiosis-related thrombosis in inflammatory bowel disease: Potential disease mechanisms and emerging therapeutic strategies,	https://doi.org/10.1016/j.thromres.2023.11.005,
Multicenter Study of Nonadherence to Self-Injectable Biologic Therapy in Patients With Inflammatory Bowel Disease: Risk Factors and Outcomes,	https://doi.org/10.1016/j.gastha.2023.01.016,
Anemia in inflammatory bowel disease course is associated with patients' worse outcome,	https://doi.org/10.1016/j.jfma.2022.11.004,
The bidirectional risk of inflammatory bowel disease and anxiety or depression: A systematic review and meta-analysis,	https://doi.org/10.1016/j.genhosppsych.2023.05.002,
Insilco prediction of the role of the FriZZled5 gene in colorectal cancer,	https://doi.org/10.1016/j.ctarc.2023.100751,
Increased risk of MAFLD and Liver Fibrosis in Inflammatory Bowel Disease Independent of Classic Metabolic Risk Factors,	https://doi.org/10.1016/j.cgh.2022.01.039,
Exosome-based bone-targeting drug delivery alleviates impaired osteoblastic bone formation and bone loss in inflammatory bowel diseases,	https://doi.org/10.1016/j.xcrm.2022.100881,
Inflammatory Bowel Disease Prevalence: Surveillance data from the U.S. National Health and Nutrition Examination Survey,	https://doi.org/10.1016/j.pmedr.2023.102173,
Oral Ritlecitinib and Brepocitinib for Moderate-to-Severe Ulcerative Colitis: Results From a Randomized, Phase 2b Study,	https://doi.org/10.1016/j.cgh.2022.12.029,
Safety and efficacy of recombinant and live herpes zoster vaccines for prevention in at-risk adults with chronic diseases and immunocompromising conditions,	https://doi.org/10.1016/j.vaccine.2022.10.063,
Enhancers of mesenchymal stem cell stemness and therapeutic potency,	https://doi.org/10.1016/j.biopha.2023.114356,
RIPK1 Mediates TNF-Induced Intestinal Crypt Apoptosis During Chronic NF-ÃŽÂºB Activation,	https://doi.org/10.1016/j.jcmgh.2019.10.002,
Total colectomy in severe Crohn's colitis in children: Is permanent ileostomy necessary?,	https://doi.org/10.1016/j.yjpso.2023.100043,
Lactobacillus casei ATCC 393 combined with vasoactive intestinal peptide alleviates dextran sodium sulfate-induced ulcerative colitis in C57BL/6 mice via NF-ÃŽÂºB and Nrf2 signaling pathways,	https://doi.org/10.1016/j.biopha.2023.115033,
Suppression of mir-150Ã¢â‚¬â€œ5p attenuates the anti-inflammatory effect of glucocorticoids in mice with ulcerative colitis,	https://doi.org/10.1016/j.molimm.2023.09.002,
A Potential Role for Stress-Induced Microbial Alterations in IgA-Associated Irritable Bowel Syndrome with Diarrhea,	https://doi.org/10.1016/j.xcrm.2020.100124,
Fecal microbiota transplantation: Emerging applications in autoimmune diseases,	https://doi.org/10.1016/j.jaut.2023.103038,
Differences and similarities between the EULAR/ASAS-EULAR and national recommendations for treatment of patients with psoriatic arthritis and axial spondyloarthritis across Europe,	https://doi.org/10.1016/j.lanepe.2023.100706,
Efficacy of Upadacitinib in a Randomized Trial of Patients With Active Ulcerative Colitis,	https://doi.org/10.1053/j.gastro.2020.02.030,

Discovery of multipotent progenitor cells from human induced membrane: Equivalent to periosteum-derived stem cells in bone regeneration,	https://doi.org/10.1016/j.jot.2023.07.004,
Nitrate ameliorates dextran sodium sulfate-induced colitis by regulating the homeostasis of the intestinal microbiota,	https://doi.org/10.1016/j.freeradbiomed.2019.12.002,
Succinate Produced by Intestinal Microbes Promotes Specification of Tuft Cells to Suppress Ileal Inflammation,	https://doi.org/10.1053/j.gastro.2020.08.029,
Microbial Signatures and Innate Immune Gene Expression in Lamina Propria Phagocytes of Inflammatory Bowel Disease Patients,	https://doi.org/10.1016/j.jcmgh.2019.10.013,
Etrasimod as induction and maintenance therapy for ulcerative colitis (ELEVATE): two randomised, double-blind, placebo-controlled, phase 3 studies,	https://doi.org/10.1016/S0140-6736(23)00061-2,
Should we use vedolizumab as mono or combo therapy in ulcerative colitis?,	https://doi.org/10.1016/j.bpg.2018.05.002,
Gut microbiota in pre-clinical rheumatoid arthritis: From pathogenesis to preventing progression,	https://doi.org/10.1016/j.jaut.2023.103001,
Fibrinogen-like protein 2 in inflammatory diseases: A future therapeutic target,	https://doi.org/10.1016/j.intimp.2023.109799,
Renal Manifestations of Inflammatory Bowel Disease,	https://doi.org/10.1016/j.rdc.2018.06.007,
Circulating integrin alpha4/beta7+ lymphocytes targeted by vedolizumab have a pro-inflammatory phenotype,	https://doi.org/10.1016/j.clim.2018.05.006,
Potential role of irisin in digestive system diseases,	https://doi.org/10.1016/j.biopha.2023.115347,
Early Ultrasound Response and Progressive Transmural Remission After Treatment With Ustekinumab in CrohnÃ¢â‚¬â„¢s Disease,	https://doi.org/10.1016/j.cgh.2022.05.055,
Perianal fistulizing Crohn's disease is associated with a higher prevalence of HPV in the anorectal fistula tract. A comparative study,	https://doi.org/10.1016/j.clinsp.2023.100219,
Single nucleotide polymorphisms in ADAM17, IL23R and SLCO1C1 genes protect against infliximab failure in adults with CrohnÃ¢â‚¬â„¢s disease,	https://doi.org/10.1016/j.biopha.2023.114225,
Role of Bifidobacterium in Modulating the Intestinal Epithelial Tight Junction Barrier: Current Knowledge and Perspectives,	https://doi.org/10.1016/j.cdnut.2023.102026,
Paneth Cell-Derived Lysozyme Defines the Composition of Mucolytic Microbiota and the Inflammatory Tone of the Intestine,	https://doi.org/10.1016/j.immuni.2020.07.010,
Nutritional and dietary strategy in the clinical care of inflammatory bowel disease,	https://doi.org/10.1016/j.jfma.2019.09.005,
Disease course and treatment outcomes of Crohn's disease patients with early or late surgery Ã¢â‚¬â€œ A Danish nationwide cohort study from 1997 to 2015,	https://doi.org/10.1016/j.dld.2022.09.016,
Potential impact of COVID-19 on colorectal disease management,	https://doi.org/10.1016/j.jcol.2020.06.002,
Prognostic models and autoimmune liver diseases,	https://doi.org/10.1016/j.bpg.2023.101878,

mTORC1 Inactivation Promotes Colitis-Induced Colorectal Cancer but Protects from APC Loss-Dependent Tumorigenesis,	https://doi.org/10.1016/j.cmet.2017.11.006,
Management of inflammatory bowel disease patients in the COVID-19 pandemic era: a Brazilian tertiary referral center guidance,	https://doi.org/10.6061/clinics/2020/e1909,
A double-edged sword: DLG5 in diseases,	https://doi.org/10.1016/j.biopha.2023.114611,
The immunomodulatory effects of probiotics and azithromycin in dextran sodium sulfate-induced ulcerative colitis in rats via TLR4-NF-ÃŽÂºB and p38-MAPK pathway,	https://doi.org/10.1016/j.biopha.2023.115005,
Transcriptome analysis in acute gastrointestinal graft-versus host disease reveals a unique signature in children and shared biology with pediatric inflammatory bowel disease,	https://doi.org/10.3324/haematol.2022.282035,
Echopattern parameter as an aid to profile Crohn's disease patients,	https://doi.org/10.1016/j.dld.2023.05.018,
Polyphenol and glucosinolate-derived AhR modulators regulate GPR15 expression on human CD4+ T cells,	https://doi.org/10.1016/j.jnutbio.2023.109456,
Treatment Patterns and Sequencing in Patients With Inflammatory Bowel Disease,	https://doi.org/10.1016/j.clinthera.2018.07.013,
Faecalibacterium prausnitzii, Bacteroides faecis and Roseburia intestinalis attenuate clinical symptoms of experimental colitis by regulating Treg/Th17 cell balance and intestinal barrier integrity,	https://doi.org/10.1016/j.biopha.2023.115568,
Earlier surgery is associated to reduced postoperative morbidity in ileocaecal Crohn's disease: Results from SURGICROHN Ã¢â‚¬â€œ LATAM study,	https://doi.org/10.1016/j.dld.2022.09.011,
Bacterial Mucosa-associated Microbiome in Inflamed and Proximal Noninflamed Ileum of Patients With Crohn’s Disease, In-flammatory Bowel Diseases	https://doi.org/10.1093/ibd/izaa107
Recent advances in shikonin for the treatment of immune-related diseases: Anti-inflammatory and immunomodulatory mechanisms,	https://doi.org/10.1016/j.biopha.2023.115138,
Unraveling the function of epithelial-mesenchymal transition (EMT) in colorectal cancer: Metastasis, therapy response, and revisiting molecular pathways,	https://doi.org/10.1016/j.biopha.2023.114395,
Platelets can reflect the severity of Crohn's disease without the effect of anemia,	https://doi.org/10.6061/clinics/2020/e1596,
Development and Validation of a Test to Monitor Endoscopic Activity in Patients With CrohnÃ¢â‚¬â„¢s Disease Based on Serum Levels of Proteins,	https://doi.org/10.1053/j.gastro.2019.10.034,
What Is the Value of Counting Mast Cells in Gastrointestinal Mucosal Biopsies?,	https://doi.org/10.1016/j.modpat.2022.100005,
6-Thioguanine inhibits rotavirus replication through suppression of Rac1 GDP/GTP cycling,	https://doi.org/10.1016/j.antiviral.2018.06.011,
Enhancing treatment success in inflammatory bowel disease: Optimising the use of anti-TNF agents and utilising their biosimilars in clinical practice,	https://doi.org/10.1016/j.dld.2020.06.008,

Major Indole Alkaloids in Evodia Rutaecarpa: The Latest Insights and Review of Their Impact on Gastrointestinal Diseases,	https://doi.org/10.1016/j.biopha.2023.115495,
Evidence of shared genetic factors in the etiology of gastrointestinal disorders and endometriosis and clinical implications for disease management,	https://doi.org/10.1016/j.xcrm.2023.101250,
Endoscopic management of strictures in patients with Crohn's disease - A multi-center experience in Taiwan,	https://doi.org/10.1016/j.jfma.2019.12.005,
Expression and function of ÃŽÂ±4ÃŽÂ²7 integrin predict the success of vedolizumab treatment in inflammatory bowel disease,	https://doi.org/10.1016/j.trsl.2022.10.003,
Osteonecrosis of the jaw in patients with inflammatory bowel disease treated with tumour necrosis factor alpha inhibitors,	https://doi.org/10.1016/j.ijom.2019.08.007,
Endothelial dysfunction and its relation in different disorders: Recent update,	https://doi.org/10.1016/j.hsr.2023.100084,
Increased risks of dental caries and periodontal disease in Chinese patients with inflammatory bowel disease,	https://doi.org/10.1111/idj.12542,
The Potential for Repurposing Anti-TNF as a Therapy for the Treatment of COVID-19,	https://doi.org/10.1016/j.medj.2020.11.005,
The landscape of immune dysregulation in CrohnÃ¢â‚¬â„¢s disease revealed through single-cell transcriptomic profiling in the ileum and colon,	https://doi.org/10.1016/j.immuni.2023.01.002,
The PERFUSE study: The experience of patients receiving Adalimumab biosimilar SB5,	https://doi.org/10.1016/j.dld.2023.05.025,
CD74 Signaling Links Inflammation to Intestinal Epithelial Cell Regeneration and Promotes Mucosal Healing,	https://doi.org/10.1016/j.jcmgh.2020.01.009,
New insights into MAIT cells in autoimmune diseases,	https://doi.org/10.1016/j.biopha.2023.114250,
SIRT2 Contributes to the Regulation of Intestinal Cell Proliferation and Differentiation,	https://doi.org/10.1016/j.jcmgh.2020.01.004,
Change in Urgency Status Among Ulcerative Colitis Patients: Understanding a Potential Unmet Patient Need From the CorEvitas Inflammatory Bowel Disease Registry,	https://doi.org/10.1016/j.gastha.2023.03.024,
Optimizing conventional DMARD therapy for SjÃƒÂ¶gren's syndrome,	https://doi.org/10.1016/j.autrev.2018.03.003,
Allosteric TYK2 inhibition: redefining autoimmune disease therapy beyond JAK1-3 inhibitors,	https://doi.org/10.1016/j.ebiom.2023.104840,
Transcriptome profiling Revealed the potential mechanisms of Shen Lin Bai Zhu San n-butanol extract on DSS induced Colitis in Mice and LC-MS analysis,	https://doi.org/10.1016/j.phymed.2023.154645,
Spermidine endows macrophages anti-inflammatory properties by inducing mitochondrial superoxide-dependent AMPK activation, Hif-1ÃŽÂ± upregulation and autophagy,	https://doi.org/10.1016/j.freeradbiomed.2020.10.029,
The crucial role of non-coding RNAs in the pathophysiology of inflammatory bowel disease,	https://doi.org/10.1016/j.biopha.2020.110507,
An integrated investigation of 16S rRNA gene sequencing and proteomics to elucidate the mechanism of Corydalis bungeana Turcz. on dextran sulfate sodium-induced colitis,	https://doi.org/10.1016/j.biopha.2023.115550,

Inflammatory Bowel Disease in Latin America: A Systematic Review,	https://doi.org/10.1016/j.vhri.2018.03.010,
Sampling for malaria molecular surveillance,	https://doi.org/10.1016/j.pt.2023.08.007,
Few and far between: clinical management of rare extranodal subtypes of mature T-cell and NK-cell lymphomas,	https://doi.org/10.3324/haematol.2023.282717,
Retinoid-Related Orphan Receptor RORÃŽÂ³t in CD4+ T-CellÃ¢â‚¬â€œMediated Intestinal Homeostasis and Inflammation,	https://doi.org/10.1016/j.ajpath.2020.07.010,
Global, regional, and national incidence of six major immune-mediated inflammatory diseases: findings from the global burden of disease study 2019,	https://doi.org/10.1016/j.eclinm.2023.102193,
Talking about Dr. Google: Communication strategies used by nurse practitioners and patients with inflammatory bowel disease in the Netherlands to discuss online health information,	https://doi.org/10.1016/j.pec.2020.01.011,
Long term management of ulcerative colitis with Faecal Microbiota Transplantation,	https://doi.org/10.1016/j.medmic.2020.100026,
Role of mesenchymal stem cells and short chain fatty acids in allergy: A prophylactic therapy for future,	https://doi.org/10.1016/j.imlet.2023.06.002,
Effects of Vedolizumab in Patients With Primary Sclerosing Cholangitis and Inflammatory Bowel Diseases,	https://doi.org/10.1016/j.cgh.2019.05.013,
Nutritional intervention with TGF-beta enriched food for special medical purposes (TGF-FSMP) is associated with a reduction of malnutrition, acute GVHD, pneumonia and may improve overall survival in patients undergoing allogeneic hematopoietic stem transplantation,	https://doi.org/10.1016/j.trim.2023.101954,
Genome-Wide Methylation Profiling in 229 Patients With CrohnÃ¢â‚¬â„¢s Disease Requiring Intestinal Resection: Epigenetic Analysis of the Trial of Prevention of Post-operative CrohnÃ¢â‚¬â„¢s Disease (TOPPIC),	https://doi.org/10.1016/j.jcmgh.2023.06.001,
Short-term anti-TNF therapy with surgical closure versus anti-TNF therapy alone for Crohn's perianal fistulas (PISA-II): long-term outcomes of an international, multicentre patient preference, randomised controlled trial,	https://doi.org/10.1016/j.eclinm.2023.102045,
Critical role of interleukin (IL)-17 in inflammatory and immune disorders: An updated review of the evidence focusing in controversies,	https://doi.org/10.1016/j.autrev.2019.102429,
Guselkumab in Patients With Moderately to Severely Active Ulcerative Colitis: QUASAR Phase 2b Induction Study,	https://doi.org/10.1053/j.gastro.2023.08.038,
Secoisolariciresinol diglucoside suppresses Dextran sulfate sodium salt-induced colitis through inhibiting NLRP1 inflammasome,	https://doi.org/10.1016/j.intimp.2019.105931,
Upadacitinib Therapy Reduces Ulcerative Colitis Symptoms as Early as Day 1 of Induction Treatment,	https://doi.org/10.1016/j.cgh.2022.11.029,

The hidden potential of glycomarkers: Glycosylation studies in the service of cancer diagnosis and treatment,	https://doi.org/10.1016/j.bbcan.2023.188889,
HLA-DQA1*05 Carriage Associated With Development of Anti-Drug Antibodies to Infliximab and Adalimumab in Patients With CrohnÃ¢â‚¬â„¢s Disease,	https://doi.org/10.1053/j.gastro.2019.09.041,
Temporal Trends in Perceptions of Anti-tumor Necrosis Factor Risks and Benefits in an Online Community of Patients With CrohnÃ¢â‚¬â„¢s Disease,	https://doi.org/10.1016/j.gastha.2022.12.007,
Taiwanese Dermatological Association (TDA) consensus recommendations for the definition, classification, diagnosis, and management of hidradenitis suppurativa,	https://doi.org/10.1016/j.jfma.2023.12.012,
A New Model of Spontaneous Colitis in Mice Induced by Deletion of an RNA m6A Methyltransferase Component METTL14 in T Cells,	https://doi.org/10.1016/j.jcmgh.2020.07.001,
Improved colonic inflammation by nervonic acid via inhibition of NF-ÃŽÂºB signaling pathway of DSS-induced colitis mice,	https://doi.org/10.1016/j.phymed.2023.154702,
Psychiatric comorbidity increases mortality in immune-mediated inflammatory diseases,	https://doi.org/10.1016/j.genhosppsych.2018.06.001,
Endotypes in bronchiectasis: moving towards precision medicine. A narrative review,	https://doi.org/10.1016/j.pulmoe.2023.03.004,
Identifying, Understanding, and Managing Fecal Urgency in Inflammatory Bowel Diseases,	https://doi.org/10.1016/j.cgh.2023.02.029,
Laboratory determination of thiopurine levels in paediatric patients with inflammatory bowel disease,	https://doi.org/10.1016/j.anpede.2019.10.008,
Rates, predictive factors and effectiveness of ustekinumab intensification to 4- or 6-weekly intervals in Crohn's disease,	https://doi.org/10.1016/j.dld.2022.10.002,
Withdrawal of infliximab or concomitant immunosuppressant therapy in patients with Crohn's disease on combination therapy (SPARE): a multicentre, open-label, randomised controlled trial,	https://doi.org/10.1016/S2468-1253(22)00385-5,
Autoimmune diseases and immune-checkpoint inhibitors for cancer therapy: review of the literature and personalized risk-based preventionÃ‚Â strategy,	https://doi.org/10.1016/j.annonc.2020.03.285,
Vaccination rate and immunity of children and adolescents with inflammatory bowel disease or autoimmune hepatitis in Germany,	https://doi.org/10.1016/j.vaccine.2019.12.024,
Metabolite interactions between host and microbiota during health and disease: Which feeds the other?,	https://doi.org/10.1016/j.biopha.2023.114295,
Understanding human health through metatranscriptomics,	https://doi.org/10.1016/j.molmed.2023.02.002,
The prognostic potential of CDX2 in colorectal cancer: Harmonizing biology and clinical practice,	https://doi.org/10.1016/j.ctrv.2023.102643,
Harnessing the microbiota for therapeutic purposes,	https://doi.org/10.1111/ajt.15753,
Non-coding RNAs in immunoregulation and autoimmunity: Technological advances and critical limitations,	https://doi.org/10.1016/j.jaut.2022.102982,

Multiple therapeutic targets in rare cholestatic liver diseases: Time to redefine treatment strategies,	https://doi.org/10.1016/j.aohep.2019.09.009,
Tumor necrosis factor inhibitor-induced pleuropericarditis: A retrospective evaluation using data from VigiBase,	https://doi.org/10.1016/j.intimp.2020.107049,
Integrative multi-omics deciphers the spatial characteristics of host-gut microbiota interactions in CrohnÃ¢â‚¬â„¢s disease,	https://doi.org/10.1016/j.xcrm.2023.101050,
Altered gut microbiome composition in nontreated plaque psoriasis patients,	https://doi.org/10.1016/j.micpath.2023.105970,
Patients With Microscopic Colitis Are at Higher Risk of Major Adverse Cardiovascular Events: A Matched Cohort Study,	https://doi.org/10.1016/j.cgh.2023.05.014,
Circadian rhythms and inflammatory diseases of the liver and gut,	https://doi.org/10.1016/j.livres.2023.08.004,
Pharmacokinetics and Exposure Response Relationships of Ustekinumab in Patients With Crohn's Disease,	https://doi.org/10.1053/j.gastro.2018.01.043,
An intestinal organoidÃ¢â‚¬â€œbased platform that recreates susceptibility to T-cellÃ¢â‚¬â€œmediated tissue injury,	https://doi.org/10.1182/blood.2019004116,
High Perceived Stress is Associated With Increased Risk of Ulcerative Colitis Clinical Flares,	https://doi.org/10.1016/j.cgh.2022.07.025,
Risk-stratified monitoring for thiopurine toxicity in immune-mediated inflammatory diseases: prognostic model development, validation, and, health economic evaluation,	https://doi.org/10.1016/j.eclinm.2023.102213,
Machine learning for metabolomics research in drug discovery,	https://doi.org/10.1016/j.ibmed.2023.100101,
Nutritional status and consumption of inflammatory and anti-inflammatory foods by patients with inflammatory bowel diseases,	https://doi.org/10.1016/j.jcol.2019.10.006,
A novel lytic bacteriophage against colistin-resistant Escherichia coli isolated from different animals,	https://doi.org/10.1016/j.virusres.2023.199090,
A target-based discovery from a parasitic helminth as a novel therapeutic approach for autoimmune diseases,	https://doi.org/10.1016/j.ebiom.2023.104751,
Comparing efficacies of autologous platelet concentrate preparations as mono-therapeutic agents in intra-bony defects through systematic review and meta-analysis,	https://doi.org/10.1016/j.jobcr.2023.08.007,
Proactive monitoring of anti-TNF agents improves follow-up of paediatric patients with Crohn disease,	https://doi.org/10.1016/j.anpede.2023.01.007,
Autologous Fat Grafting: an Emerging Treatment Option for Complex Anal Fistulas,	https://doi.org/10.1007/s11605-023-05719-4,
Faecal cytokine profiling as a marker of intestinal inflammation in acutely decompensated cirrhosis,	https://doi.org/10.1016/j.jhepr.2020.100151,
Big data-driven precision medicine: Starting the custom-made era of iatrology,	https://doi.org/10.1016/j.biopha.2020.110445,
Diagnostic delay in axial spondylarthritis: A lost battle?,	https://doi.org/10.1016/j.berh.2023.101870,

Epidemiological profile and hospitalization data of patients with inflammatory bowel diseaseÃ¢â‚¬ ,	https://doi.org/10.1016/j.jcol.2020.05.004,
Relevance of gut microbiota to Alzheimer's Disease (AD): Potential effects of probiotic in management of AD,	https://doi.org/10.1016/j.ahr.2023.100128,
Multi-omics of the gut microbial ecosystem in inflammatory bowel diseases	https://doi.org/10.1038/s41586-019-1237-9
Extracellular Matrix Bioscaffolds for Building Gastrointestinal Tissue,	https://doi.org/10.1016/j.jcmgh.2017.09.004,
The Gut Microbial Bile Acid Modulation and Its Relevance to Digestive Health and Diseases,	https://doi.org/10.1053/j.gastro.2023.02.022,
Pediatric Inflammatory Bowel Disease in Asia: Epidemiology and natural history,	https://doi.org/10.1016/j.pedneo.2019.12.008,
Clinical implications of nicotine as an antimicrobial agent and immune modulator,	https://doi.org/10.1016/j.biopha.2020.110404,
Cytokines and Immune Cell Phenotype in Acute Kidney Injury Associated With Immune Checkpoint Inhibitors,	https://doi.org/10.1016/j.ekir.2022.11.020,
Artificial Intelligence Enables Quantitative Assessment of Ulcerative Colitis Histology,	https://doi.org/10.1016/j.modpat.2023.100124,
Crosstalk within peripheral blood mononuclear cells mediates anti-inflammatory effects of n-3 PUFA-rich lipid emulsions in parenteral nutrition,	https://doi.org/10.1016/j.clnu.2023.10.016,
Bacterial dysbiosis incites Th17 cell revolt in irradiated gut,	https://doi.org/10.1016/j.biopha.2020.110674,
Dual neutralization of both interleukin 17A and interleukin 17F with bimekizumab in patients with psoriasis: Results from BE ABLE 1, a 12-week randomized, double-blinded, placebo-controlled phase 2b trial,	https://doi.org/10.1016/j.jaad.2018.03.037,
Targeting IL-23 for the interception of obesity-associated colorectal cancer,	https://doi.org/10.1016/j.neo.2023.100939,
Defining Endpoints and Biomarkers in Inflammatory Bowel Disease: Moving the Needle Through Clinical Trial Design,	https://doi.org/10.1053/j.gastro.2020.07.064,
The impact of microbiota-derived short-chain fatty acids on macrophage activities in disease: Mechanisms and therapeutic potentials,	https://doi.org/10.1016/j.biopha.2023.115276,
Updates in the diagnosis and management of small-bowel Crohn's disease,	https://doi.org/10.1016/j.bpg.2023.101855,
Oligosaccharides of Polygonatum Cyrtonema Hua ameliorates dextran sulfate sodium-induced colitis and regulates the gut microbiota,	https://doi.org/10.1016/j.biopha.2023.114562,
Beyond faecal microbiota transplantation, the non-negligible role of faecal virome or bacteriophage transplantation,	https://doi.org/10.1016/j.jmii.2023.02.005,
Faecal microbiota transplantation in the treatment of Clostridioides difficile infection,	https://doi.org/10.1016/j.humic.2020.100070,
Inflammatory bowel disease in Mexico: Epidemiology, burden of disease, and treatment trends,	https://doi.org/10.1016/j.rgmxen.2019.07.005,
Colonoscopic-Guided Pinch Biopsies in Mice as a Useful Model for Evaluating the Roles of Host and Luminal Factors in Colonic Inflammation,	https://doi.org/10.1016/j.ajpath.2018.08.016,
An Intestine-on-a-Chip Model of Plug-and-Play Modularity to Study Inflammatory Processes,	https://doi.org/10.1177/2472630320924999,

Effects of Apremilast, an Oral Inhibitor of Phosphodiesterase 4, in a Randomized Trial of Patients With Active Ulcerative Colitis,	https://doi.org/10.1016/j.cgh.2019.12.032,
Anti-inflammatory effect of wedelolactone on DSS induced colitis in rats: IL-6/STAT3 signaling pathway,	https://doi.org/10.1016/j.jaim.2022.100544,
Nature versus nurture in the spectrum of rheumatic diseases: Classification of spondyloarthritis as autoimmune or autoinflammatory,	https://doi.org/10.1016/j.autrev.2018.04.002,
Bimekizumab efficacy and safety in patients with moderate to severe plaque psoriasis: Two-year interim results from the open-label extension of the randomized BE RADIANT phase 3b trial,	https://doi.org/10.1016/j.jaad.2023.04.063,
Comparative effectiveness of ustekinumab vs. vedolizumab for anti-TNF-naÃƒÂ¯ve or anti-TNF-exposed Crohn's disease: a multicenter cohort study,	https://doi.org/10.1016/j.eclinm.2023.102337,
Relationship Between Combined Histologic and Endoscopic Endpoints and Efficacy of Ustekinumab Treatment in Patients With Ulcerative Colitis,	https://doi.org/10.1053/j.gastro.2020.08.037,
The role and mechanism of flavonoid herbal natural products in ulcerative colitis,	https://doi.org/10.1016/j.biopha.2022.114086,
Modes of therapeutic delivery in synthetic microbiology,	https://doi.org/10.1016/j.tim.2022.09.003,
The regulatory feedback of inflammatory signaling and telomere/telomerase complex dysfunction in chronic inflammatory diseases,	https://doi.org/10.1016/j.exger.2023.112132,
Methylation signatures in peripheral blood are associated with marked age acceleration and disease progression in patients with primary sclerosing cholangitis,	https://doi.org/10.1016/j.jhepr.2019.11.004,
IgG4-related cholangitis Ã¢â‚¬â€œ a mimicker of fibrosing and malignant cholangiopathies,	https://doi.org/10.1016/j.jhep.2023.08.005,
Impaired Bone Mineral Density in Pediatric Patients with Chronic Graft-versus-Host Disease,	https://doi.org/10.1016/j.bbmt.2018.02.019,
Small intestine vs. colon ecology and physiology: Why it matters in probiotic administration,	https://doi.org/10.1016/j.xcrm.2023.101190,
The relationship of major depressive disorder with Crohn's disease activity,	https://doi.org/10.1016/j.clinsp.2023.100188,
The role of host molecules in communication with the resident and pathogenic microbiota: A review,	https://doi.org/10.1016/j.medmic.2020.100005,
COX-2Ã¢â‚¬â€œPGE2 Signaling Impairs Intestinal Epithelial Regeneration and Associates with TNF Inhibitor Responsiveness in Ulcerative Colitis,	https://doi.org/10.1016/j.ebiom.2018.08.040,
Antimicrobial peptide cathelicidin LL-37 preserves intestinal barrier and organ function in rats with heat stroke,	https://doi.org/10.1016/j.biopha.2023.114565,
The role and mechanism of action of microbiota-derived short-chain fatty acids in neutrophils: From the activation to becoming potential biomarkers,	https://doi.org/10.1016/j.biopha.2023.115821,
Endoplasmic reticulum stress in the intestinal epithelium initiates purine metabolite synthesis and promotes Th17 cell differentiation in the gut,	https://doi.org/10.1016/j.immuni.2023.02.018,

Enteric Delivery of Regenerating Family Member 3 alpha AltersÃ, the Intestinal Microbiota and Controls Inflammation inÃ, Mice WithÃ, Colitis,	https://doi.org/10.1053/j.gastro.2017.11.003,
The gut ecosystem and immune tolerance,	https://doi.org/10.1016/j.jaut.2023.103114,
Effects of tumour necrosis factor on cardiovascular disease and cancer: A two-sample Mendelian randomization study,	https://doi.org/10.1016/j.ebiom.2020.102956,
Estrogen plays an important role by influencing the NLRP3 inflammasome,	https://doi.org/10.1016/j.biopha.2023.115554,
Ulcerative colitis: Gut microbiota, immunopathogenesis and application of natural products in animal models,	https://doi.org/10.1016/j.lfs.2020.118129,
Recognizing Axial Spondyloarthritis: A Guide for Primary Care,	https://doi.org/10.1016/j.mayocp.2020.02.007,
Parthenolide targets NLRP3 to treat inflammasome-related diseases,	https://doi.org/10.1016/j.intimp.2023.110229,
Ustekinumab Pharmacokinetics and Exposure Response in a Phase 3 Randomized Trial of Patients With Ulcerative Colitis,	https://doi.org/10.1016/j.cgh.2019.11.059,
NOD2 Influences Trajectories of Intestinal Microbiota Recovery After Antibiotic Perturbation,	https://doi.org/10.1016/j.jcmgh.2020.03.008,
Messages from the seventh international conference on clinical metagenomics (ICCMg7),	https://doi.org/10.1016/j.micinf.2023.105105,
Excess iron intake induced liver injury: The role of gut-liver axis and therapeutic potential,	https://doi.org/10.1016/j.biopha.2023.115728,
Naringenin: A flavanone with anti-inflammatory and anti-infective properties,	https://doi.org/10.1016/j.biopha.2023.114990,
Splanchnic vein thrombosis associated with SARS-CoV-2 infection: A VALDIG caseÃ¢â,¬â€œcontrol study,	https://doi.org/10.1016/j.jhepr.2023.100894,
Understanding the role of NLRP3-mediated pyroptosis in allergic rhinitis: A review,	https://doi.org/10.1016/j.biopha.2023.115203,
AGA-PancreasFest Joint Symposium on Exocrine Pancreatic Insufficiency,	https://doi.org/10.1016/j.gastha.2022.11.008,
Evaluation of the association of chronic inflammation and cancer: Insights and implications,	https://doi.org/10.1016/j.biopha.2023.115015,
Quorum sensing molecules as a novel microbial factor impacting muscle cells,	https://doi.org/10.1016/j.bbadis.2019.165646,
Mendelian Randomization Studies in Psoriasis and Psoriatic Arthritis: A Systematic Review,	https://doi.org/10.1016/j.jid.2022.11.014,
No Superiority of Tacrolimus Suppositories vs Beclomethasone Suppositories in a Randomized Trial of Patients With Refractory Ulcerative Proctitis,	https://doi.org/10.1016/j.cgh.2019.09.049,
Risk of Serious Infection, Opportunistic Infection, and Herpes Zoster among Patients with Psoriasis in the UnitedÃ, Kingdom,	https://doi.org/10.1016/j.jid.2018.01.039,
Immunological mechanisms of inflammatory diseases caused by gut microbiota dysbiosis: A review,	https://doi.org/10.1016/j.biopha.2023.114985,
Nephrolithiasis: Insights into Biomimics, Pathogenesis, and Pharmacology,	https://doi.org/10.1016/j.ccmp.2022.100077,
An update on the role of TRIM/NLRP3 signaling pathway in atherosclerosis,	https://doi.org/10.1016/j.biopha.2023.114321,
The Cancer Microbiome: Distinguishing Direct and Indirect Effects Requires a Systemic View,	https://doi.org/10.1016/j.trecan.2020.01.004,

Analysis of Flagellin-Specific Adaptive Immunity Reveals Links to Dysbiosis in Patients With Inflammatory Bowel Disease,	https://doi.org/10.1016/j.jcmgh.2019.11.012,
Drug Screen Identifies Leflunomide for Treatment of Inflammatory Bowel Disease Caused by TTC7A Deficiency,	https://doi.org/10.1053/j.gastro.2019.11.019,
Carcinogenesis as a Result of Multiple Inflammatory and Oxidative Hits: a Comprehensive Review from Tumor Microenvironment to Gut Microbiota,	https://doi.org/10.1016/j.neo.2018.05.002,
Dysfunctional Extracellular Matrix Remodeling Supports Perianal Fistulizing CrohnÃ¢â‚¬Â²s Disease by a Mechanoregulated Activation of the Epithelial-to-Mesenchymal Transition,	https://doi.org/10.1016/j.jcmgh.2022.12.006,
Medical Treatment Can Unintentionally Alter the Regulatory T-Cell Compartment in Patients with Widespread Pathophysiologic Conditions,	https://doi.org/10.1016/j.ajpath.2020.07.012,
Imbalance of gut microbiota is involved in the development of chronic obstructive pulmonary disease: A review,	https://doi.org/10.1016/j.biopha.2023.115150,
Phage display sequencing reveals that genetic, environmental, and intrinsic factors influence variation of human antibody epitope repertoire,	https://doi.org/10.1016/j.immuni.2023.04.003,
Role of microbiota short-chain fatty acids in the pathogenesis of autoimmune diseases,	https://doi.org/10.1016/j.biopha.2023.114620,
Ectopic expression of OX1R in ulcerative colitis mediates anti-inflammatory effect of orexin-A,	https://doi.org/10.1016/j.bbadis.2018.08.023,
Standard and Hypofractionated Dose Escalation to Intraprostatic Tumor Nodules in Localized Prostate Cancer: Efficacy and Toxicity in the DELINEATE Trial,	https://doi.org/10.1016/j.ijrobp.2019.11.402,
Comorbidities of atopic dermatitisÃ¢â‚¬â€ what does the evidence say?,	https://doi.org/10.1016/j.jaci.2022.12.002,
Meat and fiber intake and interaction with pattern recognition receptors (TLR1, TLR2, TLR4, and TLR10) in relation to colorectal cancer in a Danish prospective, case-cohort study,	https://doi.org/10.1093/ajcn/nqx011,
Common Risk Variants in AHI1 Are Associated With Childhood Steroid Sensitive Nephrotic Syndrome,	https://doi.org/10.1016/j.ekir.2023.05.018,
Development and Validation of a Scoring System to Predict Outcomes of Vedolizumab Treatment in Patients With CrohnÃ¢â‚¬â„¢sÃ‚Â Disease,	https://doi.org/10.1053/j.gastro.2018.05.039,
Erythropoietin-mediated IL-17Ã‚Â F attenuates sepsis-induced gut microbiota dysbiosis and barrier dysfunction,	https://doi.org/10.1016/j.biopha.2023.115072,
Simultaneous Loss of Both Atypical Protein Kinase C Genes in the Intestinal Epithelium Drives Serrated Intestinal Cancer by Impairing Immunosurveillance,	https://doi.org/10.1016/j.immuni.2018.09.013,
Azathioprine: its uses in dermatology,	https://doi.org/10.1016/j.abd.2020.05.003,
Topical Aminosalicylic Acid Improves Keratinocyte Differentiation in an Inducible Mouse Model of Harlequin Ichthyosis,	https://doi.org/10.1016/j.xcrm.2020.100129,

Certolizumab pegol for the treatment of chronic plaque psoriasis: Results through 48Ã‚Â weeks from 2 phase 3, multicenter, randomized, double-blinded, placebo-controlled studies (CIMPASI-1 and CIMPASI-2),	https://doi.org/10.1016/j.jaad.2018.04.012,
Colitis-Induced Microbial Perturbation Promotes Postinflammatory Visceral Hypersensitivity,	https://doi.org/10.1016/j.jcmgh.2020.04.003,
Recent update in the pathogenesis and treatment of chemotherapy and cancer induced anemia,	https://doi.org/10.1016/j.critrevonc.2019.102837,
Preparing for the incoming wave of biosimilars in oncology,	https://doi.org/10.1136/esmoopen-2018-000420,
Fibroblast Growth Factor 19 modulates intestinal microbiota and inflammation in presence of Farnesoid X Receptor,	https://doi.org/10.1016/j.ebiom.2020.102719,
The Large Hellenic Study of Uveitis: Diagnostic and Therapeutic Algorithms, Complications, and Final Outcome,	https://doi.org/10.1097/APO.0000000000000594,
Nutritional considerations for a new era: A CF foundation position paper,	https://doi.org/10.1016/j.jcf.2023.05.010,
CD147 and MMPs as key factors in physiological and pathological processes,	https://doi.org/10.1016/j.biopha.2022.113983,
Treating neutropenia and neutrophil dysfunction in glycogen storage disease type Ib with an SGLT2 inhibitor,	https://doi.org/10.1182/blood.2019004465,
Messages from the Fourth International Conference on Clinical Metagenomics,	https://doi.org/10.1016/j.micinf.2020.07.007,
Impact of surgery and its complications in ulcerative colitis patients in clinical practice: A systematic literature review of real-world evidence in Europe,	https://doi.org/10.1016/j.ijso.2019.11.010,
Gut microbiota as an Ã¢â‚¬Å"invisible organÃ¢â‚¬Â that modulates the function of drugs,	https://doi.org/10.1016/j.biopha.2019.109653,
Perturbed Mitochondrial Dynamics Is a Novel Feature of Colitis That Can Be Targeted to Lessen Disease,	https://doi.org/10.1016/j.jcmgh.2020.04.004,
A randomized, placebo-controlled, phase II study of obeticholic acid for primary sclerosing cholangitis,	https://doi.org/10.1016/j.jhep.2020.02.033,
Carotenoids in Health as Studied by Omics-Related Endpoints,	https://doi.org/10.1016/j.advnut.2023.09.002,
Role of Interleukin-22 in ulcerative colitis,	https://doi.org/10.1016/j.biopha.2023.114273,
Inflammatory Bowel Disease Types Differ in Markers of Inflammation, Gut Barrier and in Specific Anti-Bacterial Response	https://doi.org/10.3390/cells8070719,
Biofilm modifiers: The disparity in paradigm of oral biofilm ecosystem,	https://doi.org/10.1016/j.biopha.2023.114966,
SLC26A3 (DRA) prevents TNF-alpha-induced barrier dysfunction and dextran sulfate sodium-induced acute colitis,	https://doi.org/10.1038/s41374-017-0005-4,
The Ã¢â‚¬Å"CultureÃ¢â‚¬Â of Pain Control: A Review of Opioid-Induced Dysbiosis (OID) in Antinociceptive Tolerance,	https://doi.org/10.1016/j.jpain.2019.11.015,
Spondyloarthritis-Associated IgA Nephropathy,	https://doi.org/10.1016/j.ekir.2020.03.012,

Deletion of miR-150 Prevents Spontaneous T Cell Proliferation and the Development of Colitis,	https://doi.org/10.1016/j.gastha.2023.01.021,
Gut dysbiosis-derived ÃŽÂ²-glucuronidase promotes the development of endometriosis,	https://doi.org/10.1016/j.fertnstert.2023.03.032,
The Adaptor Protein CARD9 Protects against Colon Cancer by Restricting Mycobiota-Mediated Expansion of Myeloid-Derived Suppressor Cells,	https://doi.org/10.1016/j.immuni.2018.08.018,
Diacerein: Recent insight into pharmacological activities and molecular pathways,	https://doi.org/10.1016/j.biopha.2020.110594,
An integrated fecal microbiome and metabolome in the aged mice reveal anti-aging effects from the intestines and biochemical mechanism of FuFang zhenshu TiaoZhi(FTZ),	https://doi.org/10.1016/j.biopha.2019.109421,
Use of Single-Cell -Omic Technologies to Study the Gastrointestinal Tract and Diseases, From Single Cell Identities to Patient Features,	https://doi.org/10.1053/j.gastro.2020.04.073,
Chinese herbal medicines for treating ulcerative colitis via regulating gut microbiota-intestinal immunity axis,	https://doi.org/10.1016/j.chmed.2023.03.003,
Efficacy and Safety of Upadacitinib in a Randomized Trial of Patients With CrohnÃ¢â‚¬â„¢s Disease,	https://doi.org/10.1053/j.gastro.2020.01.047,
MicroRNAs in autoimmune liver diseases: from diagnosis to potential therapeutic targets,	https://doi.org/10.1016/j.biopha.2020.110558,
Gut microbiota and Autism Spectrum Disorder: From pathogenesis to potential therapeutic perspectives,	https://doi.org/10.1016/j.jtcme.2022.03.001,
Pathogenesis of Fistulating CrohnÃ¢â‚¬â„¢s Disease: A Review,	https://doi.org/10.1016/j.jcmgh.2022.09.011,
Dietary and Microbial Determinants in Food Allergy,	https://doi.org/10.1016/j.immuni.2020.07.025,
Impact of key manifestations of psoriatic arthritis on patient quality of life, functional status, and work productivity: Findings from a real-world study in the United States and Europe,	https://doi.org/10.1016/j.jbspin.2023.105534,
Chromatin dynamics and histone modifications in intestinal microbiota-host crosstalk,	https://doi.org/10.1016/j.molmet.2019.12.005,
Tigecycline reduces tumorigenesis in colorectal cancer via inhibition of cell proliferation and modulation of immune response,	https://doi.org/10.1016/j.biopha.2023.114760,
Non-invasively enhanced intracranial transplantation of mesenchymal stem cells using focused ultrasound mediated by overexpression of cell-adhesion molecules,	https://doi.org/10.1016/j.scr.2020.101726,
Ureteral Stent Microbiota Is Associated with Patient Comorbidities but Not Antibiotic Exposure,	https://doi.org/10.1016/j.xcrm.2020.100094,
Advancing Intestinal Organoid Technology Toward RegenerativeÃ‚Â Medicine,	https://doi.org/10.1016/j.jcmgh.2017.10.006,
Sanhuang Shu'ai decoction alleviates DSS-induced ulcerative colitis via regulation of gut microbiota, inflammatory mediators and cytokines,	https://doi.org/10.1016/j.biopha.2020.109934,
The Biology of T Regulatory Type 1 Cells and Their Therapeutic Application in Immune-Mediated Diseases,	https://doi.org/10.1016/j.immuni.2018.12.001,
Clinical characteristics and comorbidities in psoriatic arthritis: Experience from a single rheumatology centre in Malaysia,	https://doi.org/10.1016/j.ejr.2023.08.002,

Microfluidic Organ-on-a-Chip Models of Human Intestine,	https://doi.org/10.1016/j.jcmgh.2017.12.010,
Microbiota-derived short-chain fatty acids and modulation of host-derived peptides formation: Focused on host defense peptides,	https://doi.org/10.1016/j.biopha.2023.114586,
The clinical relevance of the adhesion G protein-coupled receptor F5 for human diseases and cancers,	https://doi.org/10.1016/j.bbadis.2023.166683,
Cellular Red-Ox system in health and disease: The latest update,	https://doi.org/10.1016/j.biopha.2023.114606,
Artificial Intelligence Enabled Histological Prediction of Remission or Activity and Clinical Outcomes in Ulcerative Colitis,	https://doi.org/10.1053/j.gastro.2023.02.031,
Strategy and application of manipulating DCs chemotaxis in disease treatment and vaccine design,	https://doi.org/10.1016/j.biopha.2023.114457,
The link between rheumatic disorders and inborn errors of immunity,	https://doi.org/10.1016/j.ebiom.2023.104501,
Posttranslational modifications in psoriatic arthritis: A systematic literature review,	https://doi.org/10.1016/j.autrev.2023.103393,
Boosting mTOR-dependent autophagy via upstream TLR4-MyD88-MAPK signalling and downstream NF-ÃŽÂºB pathway quenches intestinal inflammation and oxidative stress injury,	https://doi.org/10.1016/j.ebiom.2018.08.035,
Rectal polypoid Inflammatory Myofibroblastic Tumor in 39-year-old liver transplant recipient with de-novo ulcerative colitis: A case report and literature review,	https://doi.org/10.1016/j.ehpc.2018.02.003,
The Untapped Opportunity and Challenge of Immunometabolism: A New Paradigm for Drug Discovery,	https://doi.org/10.1016/j.cmet.2019.11.014,
Antibiotic intervention exacerbated oxidative stress and inflammatory responses in SD rats under hypobaric hypoxia exposure,	https://doi.org/10.1016/j.freeradbiomed.2023.10.002,
The epithelial barrier: The gateway to allergic, autoimmune, and metabolic diseases and chronic neuropsychiatric conditions,	https://doi.org/10.1016/j.smim.2023.101846,
DR3 Regulates Intestinal Epithelial Homeostasis and Regeneration After Intestinal Barrier Injury,	https://doi.org/10.1016/j.jcmgh.2023.03.008,
Psoriasis in Taiwan: From epidemiology to new treatments,	https://doi.org/10.1016/j.dsi.2018.06.001,
Lactobacillus plantarum modulate gut microbiota and intestinal immunity in cyclophosphamide-treated mice model,	https://doi.org/10.1016/j.biopha.2023.115812,
DOCK11 deficiency in patients with X-linked actinopathy and autoimmunity,	https://doi.org/10.1182/blood.2022018486,
Modes of therapeutic delivery in synthetic microbiology,	https://doi.org/10.1016/j.tim.2022.09.003,
Regulation of host immune responses by Lactobacillus through aryl hydrocarbon receptors,	https://doi.org/10.1016/j.medmic.2023.100081,
Tissue alarmins and adaptive cytokine induce dynamic and distinct transcriptional responses in tissue-resident intraepithelial cytotoxic T lymphocytes,	https://doi.org/10.1016/j.jaut.2020.102422,
Guiqi Baizhu prescription ameliorates cytarabine-induced intestinal mucositis by targeting JAK2 to inhibit M1 macrophage polarization,	https://doi.org/10.1016/j.biopha.2023.114902,

Machine learning identifies signatures of macrophage reactivity and tolerance that predict disease outcomes,	https://doi.org/10.1016/j.ebiom.2023.104719,
The role of cannabidiol in aging,	https://doi.org/10.1016/j.biopha.2023.115074,
JAK-STAT pathway inhibitors in dermatology,	https://doi.org/10.1016/j.abd.2023.03.001,
IL22BP Mediates the Antitumor Effects of Lymphotoxin Against Colorectal Tumors in Mice and Humans,	https://doi.org/10.1053/j.gastro.2020.06.033,
Drugs targeting adenosine signaling pathways: A current view,	https://doi.org/10.1016/j.biopha.2023.115184,
Hepatitis B virus reactivation in patients treated with immunosuppressive drugs: a practical guide for clinicians,	https://doi.org/10.7861/clinmedicine.18-3-212,
Role of Nox4 in Mitigating Inflammation and Fibrosis in Dextran Sulfate SodiumÃ¢â‚¬â€œInduced Colitis,	https://doi.org/10.1016/j.jcmgh.2023.05.002,
Targeted lactate dehydrogenase genes silencing in probiotic lactic acid bacteria: A possible paradigm shift in colorectal cancer treatment?,	https://doi.org/10.1016/j.biopha.2023.114371,
Hematopoiesis and the bacterial microbiome,	https://doi.org/10.1182/blood-2018-02-832519,
T helper 17 (Th17) cell responses to the gut microbiota in human diseases,	https://doi.org/10.1016/j.biopha.2023.114483,
ASBT(SLC10A2): A promising target for treatment of diseases and drug discovery,	https://doi.org/10.1016/j.biopha.2020.110835,
An Imperative Need for Further Genetic Studies of Alopecia Areata,	https://doi.org/10.1016/j.jisp.2020.04.003,
Host-Gut Microbiota Crosstalk in Intestinal Adaptation,	https://doi.org/10.1016/j.jcmgh.2018.01.024,
Deficiency of the CD155-CD96 immune checkpoint controls IL-9 production in giant cell arteritis,	https://doi.org/10.1016/j.xcrm.2023.101012,
Clinical and Pharmacokinetic Factors Associated With Adalimumab-Induced Mucosal Healing in Patients With CrohnÃ¢â‚¬â„¢sÃ‚Â Disease,	https://doi.org/10.1016/j.cgh.2017.10.036,
InÃ‚Â Vitro Efficacy of Targeted Fermentable Oligosaccharides, Disaccharides, Monosaccharides, and Polyols Enzymatic Digestion in a High-Fidelity Simulated Gastrointestinal Environment,	https://doi.org/10.1016/j.gastha.2022.10.011,
Acute infection with Strongyloides venezuelensis increases intestine production IL-10, reduces Th1/Th2/Th17 induction in colon and attenuates Dextran Sulfate Sodium-induced colitis in BALB/c mice,	https://doi.org/10.1016/j.cyto.2018.08.003,
Enhanced O-linked Glcnacylation in Crohn's disease promotes intestinal inflammation,	https://doi.org/10.1016/j.ebiom.2020.102693,
miR2Pathway: A novel analytical method to discover MicroRNA-mediated dysregulated pathways involved in hepatocellular carcinoma,	https://doi.org/10.1016/j.jbi.2018.03.013,
Addressing education and employment outcomes in the provision of healthcare for young people with physical long-term conditions: A systematic review and mixed methods synthesis,	https://doi.org/10.1016/j.pec.2023.107765,
The Intestinal Microbiota in Colorectal Cancer,	https://doi.org/10.1016/j.ccell.2018.03.004,

Population Pharmacokinetics and ExposureÃ¢â‚¬â€œResponse Modeling of Golimumab in Adults With Moderately to Severely Active Ulcerative Colitis,	https://doi.org/10.1016/j.clinthera.2019.11.010,
Nuclear receptor FXR, bile acids and liver damage: Introducing the progressive familial intrahepatic cholestasis with FXR mutations,	https://doi.org/10.1016/j.bbadis.2017.09.019,
Integrating ayurvedic medicine into cancer research programs part 1: Ayurveda background and applications,	https://doi.org/10.1016/j.jaim.2022.100676,
Necroptosis in the Pathophysiology of Disease,	https://doi.org/10.1016/j.ajpath.2019.10.012,
Learning from the nexus of autoimmunity and cancer,	https://doi.org/10.1016/j.immuni.2023.01.022,
Effectiveness, safety and drug survival of tumor necrosis factor-ÃŽÂ± inhibitors in the treatment of spondyloarthritis: A real-life study in Tunisia,	https://doi.org/10.1016/j.ejr.2017.10.007,
Oenothera rosea LÃ‚Â´HÃƒÂ©r. ex Ait attenuates acute colonic inflammation in TNBS-induced colitis model in rats: in vivo and in silico myeloperoxidase role,	https://doi.org/10.1016/j.biopha.2018.09.081,
Genes associated with venous thromboembolism in colorectal cancer patients,	https://doi.org/10.1111/jth.13926,
Microbiota-derived tryptophan metabolism: Impacts on health, aging, and disease,	https://doi.org/10.1016/j.exger.2023.112319,
Modulation of Host Immunity by Helminths: The Expanding Repertoire of Parasite Effector Molecules,	https://doi.org/10.1016/j.immuni.2018.10.016,
A novel gluten knowledge base of potential biomedical and health-related interactions extracted from the literature: Using machine learning and graph analysis methodologies to reconstruct the bibliome,	https://doi.org/10.1016/j.jbi.2023.104398,
Gastrointestinal peptides and small-bowel hypomotility are possible causes for fasting and postprandial symptoms in active CrohnÃ¢â‚¬â„¢s disease,	https://doi.org/10.1093/ajcn/nqz240,
Intestinal Epithelial Wnt Signaling Mediates Acetylcholine-Triggered Host Defense against Infection,	https://doi.org/10.1016/j.immuni.2018.04.017,
Annual review of selected scientific literature: A report of the Committee on Scientific Investigation of the American Academy of Restorative Dentistry,	https://doi.org/10.1016/j.prosdent.2018.09.010,
Cholangiopathies Ã¢â‚¬â€œ Towards a molecular understanding,	https://doi.org/10.1016/j.ebiom.2018.08.024,
One hundred citation classics in benign anorectal disease: a bibliometric analysis of seven decades (1950Ã¢â‚¬â€œ2019),	https://doi.org/10.1016/j.jcol.2019.10.002,
Intermittent Fasting Confers Protection in CNS Autoimmunity by Altering the Gut Microbiota,	https://doi.org/10.1016/j.cmet.2018.05.006,
Correlates of immune exacerbations in leprosy,	https://doi.org/10.1016/j.smim.2018.06.003,
Innately versatile: ÃŽÂ³ÃŽÂ´17Ã‚Â T cells in inflammatory and autoimmune diseases,	https://doi.org/10.1016/j.jaut.2017.11.006,
An unsupervised machine learning method for discovering patient clusters based on genetic signatures,	https://doi.org/10.1016/j.jbi.2018.07.004,

NHERF1 Between Promises and Hopes: Overview on Cancer and Prospective Openings,	https://doi.org/10.1016/j.tranon.2018.01.006,
Lactobacillus acidophilus inhibits bone loss and increases bone heterogeneity in osteoporotic mice via modulating Treg-Th17 cell balance,	https://doi.org/10.1016/j.bonr.2018.02.001,
Emerging nanotherapeutic strategies targeting gut-X axis against diseases,	https://doi.org/10.1016/j.biopha.2023.115577,
The effect of probiotics on immune responses and their therapeutic application: A new treatment option for multiple sclerosis,	https://doi.org/10.1016/j.biopha.2022.114195,
Synthesis of novel 2, 3, 5-tri-substituted thiazoles with anti-inflammatory and antibacterial effect causing clinical pathogens,	https://doi.org/10.1016/j.jiph.2020.02.002,
Profiling of Human Gut Virome with Oxford Nanopore Technology,	https://doi.org/10.1016/j.medmic.2020.100012,
Low-grade intestinal inflammation two decades after pelvic radiotherapy,	https://doi.org/10.1016/j.ebiom.2023.104691,
Patients with early-onset rectal cancer aged 40 year or less have similar oncologic outcomes to older patients despite presenting in more advanced stage; A retrospective cohort study,	https://doi.org/10.1016/j.ijsu.2020.09.029,
Salvia plebeia R. Br. : an overview about its traditional uses, chemical constituents, pharmacology and modern applications,	https://doi.org/10.1016/j.biopha.2019.109589,
Chinese herbal medicine versus probiotics for irritable bowel syndrome: A systematic review and meta-analysis of randomized controlled trials,	https://doi.org/10.1016/j.eujim.2020.101177,
Adding Chinese herbal medicine to probiotics for irritable bowel syndrome-diarrhea: A systematic review and meta-analysis of randomized controlled trials,	https://doi.org/10.1016/j.jtcms.2020.01.004,
Mitochondrial Oxidative Damage Underlies Regulatory T Cell Defects in Autoimmunity,	https://doi.org/10.1016/j.cmet.2020.07.001,
Autoimmune/inflammatory syndrome induced by adjuvants (ASIA) in 2023,	https://doi.org/10.1016/j.autrev.2023.103287,
Genomic profiling in advanced stage non-small-cell lung cancer patients with platinum-based chemotherapy identifies germline variants with prognostic value in SMYD2,	https://doi.org/10.1016/j.ctarc.2018.02.003,
Does Modification of the Large Intestinal Microbiome Contribute to the Anti-Inflammatory Activity of Fermentable Fiber?,	https://doi.org/10.3945/cdn.117.001180,
Effect of Vicenin-2 on ovariectomy-induced osteoporosis in rats,	https://doi.org/10.1016/j.biopha.2020.110474,
Clinical significance of Galectin-1 and Galectin-4 in rheumatoid arthritis patients and their potential role as diagnostic markers,	https://doi.org/10.1016/j.ejr.2022.11.009,
Proteostasis in T cell aging,	https://doi.org/10.1016/j.smim.2023.101838,
Complex pain in children and young people; part 2: management,	https://doi.org/10.1016/j.bjae.2017.12.001,
Genomic integrity of human induced pluripotent stem cells across nine studies in the NHLBI NextGen program,	https://doi.org/10.1016/j.scr.2020.101803,
Regulation of the Immune Response by the Aryl Hydrocarbon Receptor,	https://doi.org/10.1016/j.immuni.2017.12.012,

Long-term outcome of LRBA deficiency in 76 patients after various treatment modalities as evaluated by the immune deficiency and dysregulation activity (IDDA) score,	https://doi.org/10.1016/j.jaci.2019.12.896,
Knockdown of SIRT3 perturbs protective effects of irisin against bone loss in diabetes and periodontitis,	https://doi.org/10.1016/j.freeradbiomed.2023.02.023,
Isorhamnetin: A review of pharmacological effects,	https://doi.org/10.1016/j.biopha.2020.110301,
**SCIENCEDIRECT - GUT MICROBIOME AND BIOLOGICAL THERAPIES (521 RESULTS)**	
Gut microbiome in pediatric acute leukemia: from predisposition to cure,	https://doi.org/10.1182/bloodadvances.2021005129,
Recent insights into the role of the microbiome in malignant and benign hematologic diseases,	https://doi.org/10.1016/j.critrevonc.2021.103289,
Exploring the microbiome: Uncovering the link with lung cancer and implications for diagnosis and treatment,	https://doi.org/10.1016/j.pccm.2023.08.003,
Circadian rhythms and inflammatory diseases of the liver and gut,	https://doi.org/10.1016/j.livres.2023.08.004,
Gut microbiota-derived metabolites as key actors in type 2 diabetes mellitus,	https://doi.org/10.1016/j.biopha.2022.112839,
Nonalcoholic Fatty Liver Disease and the Gut-Liver Axis: Exploring an Undernutrition Perspective,	https://doi.org/10.1053/j.gastro.2022.01.058,
Intestinal Organoids: A Tool for Modelling DietÃ¢â‚¬â€œMicrobiomeÃ¢â‚¬â€œHost Interactions,	https://doi.org/10.1016/j.tem.2020.02.004,
The role of the microbiome in diabetes mellitus,	https://doi.org/10.1016/j.diabres.2020.108645,
Fecal microbiota transplantation: Emerging applications in autoimmune diseases,	https://doi.org/10.1016/j.jaut.2023.103038,
Prostate diseases and microbiome in the prostate, gut, and urine,	https://doi.org/10.1016/j.prnil.2022.03.004,
Differences in Gut Microbiota in Patients With vs Without Inflammatory Bowel Diseases: A Systematic Review	https://doi.org/10.1053/j.gastro.2019.11.294,
Gut microbiota controlling radiation-induced enteritis and intestinal regeneration,	https://doi.org/10.1016/j.tem.2023.05.006,
Is it possible to intervene early cirrhosis by targeting toll-like receptors to rebalance the intestinal microbiome?,	https://doi.org/10.1016/j.intimp.2022.109627,
Circadian Rhythms, the Gut Microbiome, and Metabolic Disorders,	https://doi.org/10.1016/j.gastha.2021.10.008,
The Gut-Peritoneum Axis in Peritoneal Dialysis andÃ‚Â Peritoneal Fibrosis,	https://doi.org/10.1016/j.xkme.2023.100645,
Modulation of gut microbiota: An emerging consequence in neonatal sepsis,	https://doi.org/10.1016/j.cegh.2023.101245,
Gum-gut axis: The potential role of salivary biomarkers in the diagnosis and monitoring progress of inflammatory bowel diseases,	https://doi.org/10.1016/j.sdentj.2022.12.006,
Fueling Gut Microbes: A Review of the Interaction between Diet, Exercise, and the Gut Microbiota in Athletes,	https://doi.org/10.1093/advances/nmab077,
The role of the gut microbiome in eye diseases,	https://doi.org/10.1016/j.preteyeres.2022.101117,

Implications of the microbiome in the development and treatment of pancreatic cancer: Thinking outside of the box by looking inside the gut,	https://doi.org/10.1016/j.neo.2020.12.008,
Gut microbiota in pre-clinical rheumatoid arthritis: From pathogenesis to preventing progression,	https://doi.org/10.1016/j.jaut.2023.103001,
Skin microbiome of atopic dermatitis,	https://doi.org/10.1016/j.alit.2021.11.001,
Impact of gut-microbiome altering drugs and fecal microbiota transplant on the efficacy and toxicity of immune checkpoint inhibitors: A systematic review,	https://doi.org/10.1016/j.adcanc.2021.100020,
Gut dysbiosis and age-related neurological diseases in females,	https://doi.org/10.1016/j.nbd.2022.105695,
Unraveling the role of medicinal plants and Gut microbiota in colon cancer: Towards microbiota-based strategies for prevention and treatment,	https://doi.org/10.1016/j.hsr.2023.100115,
HIV susceptibility in women: The roles of genital inflammation, sexually transmitted infections and the genital microbiome,	https://doi.org/10.1016/j.jri.2021.103291,
Current understanding of the intratumoral microbiome in various tumors,	https://doi.org/10.1016/j.xcrm.2022.100884,
Role of gut microbiota metabolism and biotransformation on dietary natural products to human health implications with special reference to biochemoinformatics approach,	https://doi.org/10.1016/j.jtcme.2022.03.005,
New technologies for developing phage-based tools to manipulate the human microbiome,	https://doi.org/10.1016/j.tim.2021.04.007,
Platelets bridging the gap between gut dysbiosis and neuroinflammation in stress-linked disorders: A narrative review,	https://doi.org/10.1016/j.jneuroim.2023.578155,
Relevance of gut microbiota to Alzheimer's Disease (AD): Potential effects of probiotic in management of AD,	https://doi.org/10.1016/j.ahr.2023.100128,
Emulating the gutÃ¢â‚¬â€œliver axis: Dissecting the microbiome's effect on drug metabolism using multiorgan-on-chip models,	https://doi.org/10.1016/j.coemr.2021.03.003,
Implications of gut and oral microbiota in neuroinflammatory responses in Alzheimer's disease,	https://doi.org/10.1016/j.lfs.2023.122132,
Finding intestinal fortitude: Integrating the microbiome into a holistic view of depression mechanisms, treatment, and resilience,	https://doi.org/10.1016/j.nbd.2019.104578,
Gut vascular barrier in the pathogenesis and resolution of Crohn's disease: A novel link from origination to therapy,	https://doi.org/10.1016/j.clim.2023.109683,
Correlation between human gut microbiome and diseases,	https://doi.org/10.1016/j.imj.2022.08.004,
A comparative study of the gut microbiota in immune-mediated inflammatory diseases-does a common dysbiosis exist?	https://doi.org/10.1186/s40168-018-0603-4,
The Gut Microbial Bile Acid Modulation and Its Relevance to Digestive Health and Diseases,	https://doi.org/10.1053/j.gastro.2023.02.022,
Circadian rhythms and the gut microbiome synchronize the hostÃ¢â‚¬â„¢s metabolic response to diet,	https://doi.org/10.1016/j.cmet.2021.03.015,

Vitamins, the gut microbiome and gastrointestinal health in humans,	https://doi.org/10.1016/j.nutres.2021.09.001,
Gut microbiome in modulating immune checkpoint inhibitors,	https://doi.org/10.1016/j.ebiom.2022.104163,
Exercise, diet and stress as modulators of gut microbiota: Implications for neurodegenerative diseases,	https://doi.org/10.1016/j.nbd.2019.104621,
The respiratory microbiome in childhood asthma,	https://doi.org/10.1016/j.jaci.2023.10.001,
Microbiome and cancer,	https://doi.org/10.1016/j.ccell.2021.08.006,
Microbiome in Colorectal Cancer: How to Get from Meta-omics to Mechanism?,	https://doi.org/10.1016/j.tim.2020.01.001,
Causal discovery for the microbiome,	https://doi.org/10.1016/S2666-5247(22)00186-0,
Short chain fatty acids and its producing organisms: An overlooked therapy for IBD?,	https://doi.org/10.1016/j.ebiom.2021.103293,
Microbiome-phage interactions in inflammatory bowel disease,	https://doi.org/10.1016/j.cmi.2022.08.027,
Gut mycobiome in metabolic diseases: Mechanisms and clinical implication,	https://doi.org/10.1016/j.bj.2023.100625,
A cross-talk between gut microbiome, salt and hypertension,	https://doi.org/10.1016/j.biopha.2020.111156,
Emerging nanotherapeutic strategies targeting gut-X axis against diseases,	https://doi.org/10.1016/j.biopha.2023.115577,
Associations between the gut microbiome, gut microbiology and heart failure: Current understanding and future directions,	https://doi.org/10.1016/j.ahjo.2022.100150,
The possible role of oral microbiome in autoimmunity,	https://doi.org/10.1016/j.ijwd.2020.07.011,
Evidence for immune system alterations in peripheral biological fluids in Parkinson's disease,	https://doi.org/10.1016/j.nbd.2022.105744,
Gut microbiome effects on neuronal excitability & activity: Implications for epilepsy,	https://doi.org/10.1016/j.nbd.2022.105629,
Emerging targetome and signalome landscape of gut microbial metabolites,	https://doi.org/10.1016/j.cmet.2021.12.011,
Bile acid receptors and signaling crosstalk in the liver, gut and brain,	https://doi.org/10.1016/j.livres.2021.07.002,
Oxytocin and the microbiome,	https://doi.org/10.1016/j.cpnec.2023.100205,
Gut microbiota interactions with antitumor immunity in colorectal cancer: From understanding to application,	https://doi.org/10.1016/j.biopha.2023.115040,
Increased Tryptophan Metabolism Is Associated With Activity of Inflammatory Bowel Diseases	https://doi.org/10.1053/j.gastro.2017.08.028
Phage delivered CRISPR-Cas system to combat multidrug-resistant pathogens in gut microbiome,	https://doi.org/10.1016/j.biopha.2022.113122,
The gut ecosystem and immune tolerance,	https://doi.org/10.1016/j.jaut.2023.103114,
Modulation of gut microbiota by foods and herbs to prevent cardiovascular diseases,	https://doi.org/10.1016/j.jtcme.2021.09.006,
The Brain-Gut-Microbiome Axis,	https://doi.org/10.1016/j.jcmgh.2018.04.003,
Microbiome-based interventions: therapeutic strategies in cancer immunotherapy,	https://doi.org/10.1016/j.iotech.2020.11.001,
Hematopoiesis and the bacterial microbiome,	https://doi.org/10.1182/blood-2018-02-832519,
The changes in cognitive function following bariatric surgery considering the function of gut microbiome,	https://doi.org/10.1016/j.obpill.2022.100020,

T helper 17 (Th17) cell responses to the gut microbiota in human diseases,	https://doi.org/10.1016/j.biopha.2023.114483,
Small molecules in the big picture of gut microbiome-host cross-talk,	https://doi.org/10.1016/j.ebiom.2022.104085,
The gut microbes in inflammatory bowel disease: Future novel target option for pharmacotherapy,	https://doi.org/10.1016/j.biopha.2023.114893,
Current Concepts, Opportunities, and Challenges of Gut Microbiome-Based Personalized Medicine in Nonalcoholic Fatty Liver Disease,	https://doi.org/10.1016/j.cmet.2020.11.010,
Gut-muscle axis and sepsis-induced myopathy: The potential role of gut microbiota,	https://doi.org/10.1016/j.biopha.2023.114837,
Vitamin D and Microbiome: Molecular Interaction in Inflammatory Bowel Disease Pathogenesis,	https://doi.org/10.1016/j.ajpath.2023.02.004,
Current status of intratumour microbiome in cancer and engineered exogenous microbiota as a promising therapeutic strategy,	https://doi.org/10.1016/j.biopha.2021.112443,
Decoding the neurocircuitry of gut feelings: Region-specific microbiome-mediated brain alterations,	https://doi.org/10.1016/j.nbd.2023.106033,
Microbiome and the immune system: From a healthy steady-state to allergy associated disruption,	https://doi.org/10.1016/j.humic.2018.10.001,
The respiratory microbiome after lung transplantation: Reflection or driver of respiratory disease?,	https://doi.org/10.1111/ajt.16568,
Impact of the microbiome on human, animal, and environmental health from a One Health perspective,	https://doi.org/10.1016/j.soh.2023.100037,
Phage therapy: Targeting intestinal bacterial microbiota for the treatment of liver diseases,	https://doi.org/10.1016/j.jhepr.2023.100909,
Gut-liver axis: Pathophysiological concepts and clinical implications,	https://doi.org/10.1016/j.cmet.2022.09.017,
Biogeographical effect on the diversity of vaginal microbiome in preterm birth: A systematic review and meta-analysis,	https://doi.org/10.1016/j.medmic.2023.100080,
Reconsidering ventilator-associated pneumonia from a new dimension of the lung microbiome,	https://doi.org/10.1016/j.ebiom.2020.102995,
Immunological mechanisms of inflammatory diseases caused by gut microbiota dysbiosis: A review,	https://doi.org/10.1016/j.biopha.2023.114985,
Diet-gut microbiota-epigenetics in metabolic diseases: From mechanisms to therapeutics,	https://doi.org/10.1016/j.biopha.2022.113290,
Gut-oriented disease modifying therapy for Parkinson's disease,	https://doi.org/10.1016/j.jfma.2022.09.010,
Possibilities and limitations of using low biomass samples for urologic disease and microbiome research,	https://doi.org/10.1016/j.prnil.2022.10.001,
The Cancer Microbiome: Distinguishing Direct and Indirect Effects Requires a Systemic View,	https://doi.org/10.1016/j.trecan.2020.01.004,
Microbiome in cancer: Role in carcinogenesis and impact in therapeutic strategies,	https://doi.org/10.1016/j.biopha.2022.112898,
The Role of the Gut Microbiota in Dietary Interventions for Depression and Anxiety,	https://doi.org/10.1093/advances/nmaa016,
Gut dysbiosis-related thrombosis in inflammatory bowel disease: Potential disease mechanisms and emerging therapeutic strategies,	https://doi.org/10.1016/j.thromres.2023.11.005,

Imbalance of gut microbiota is involved in the development of chronic obstructive pulmonary disease: A review,	https://doi.org/10.1016/j.biopha.2023.115150,
Gut microbiota in the pathogenesis and therapeutic approaches of diabetes,	https://doi.org/10.1016/j.ebiom.2023.104821,
Traditional Chinese medicine for the treatment of Alzheimer's disease: A focus on the microbiotaÃ¢â,¬â€œgutÃ¢â,¬â€œbrain axis,	https://doi.org/10.1016/j.biopha.2023.115244,
The gut virome: A new microbiome component in health and disease,	https://doi.org/10.1016/j.ebiom.2022.104113,
Microbiota and the gut-brain-axis: Implications for new therapeutic design in the CNS,	https://doi.org/10.1016/j.ebiom.2022.103908,
Sexual dimorphism of cardiometabolic dysfunction: Gut microbiome in the play?,	https://doi.org/10.1016/j.molmet.2018.05.016,
The role of gut microbiome in cancer genesis and cancer prevention,	https://doi.org/10.1016/j.hsr.2021.100010,
The microbiome: An emerging key player in aging and longevity,	https://doi.org/10.1016/j.tma.2020.07.004,
Growth Effects of N-Acylethanolamines on Gut Bacteria Reflect Altered Bacterial Abundances in Inflammatory Bowel Disease	https://doi.org/10.1038/s41564-019-0655-7,
Gut Microbiota and its Metabolites: Bridge of Dietary Nutrients and AlzheimerÃ¢â,¬â„¢s Disease,	https://doi.org/10.1016/j.advnut.2023.04.005,
The intestinal microbiota in colorectal cancer metastasis Ã¢â,¬â€œ Passive observer or key player?,	https://doi.org/10.1016/j.critrevonc.2022.103856,
Harnessing the microbiota for therapeutic purposes,	https://doi.org/10.1111/ajt.15753,
Biomarkers for immune checkpoint therapy targeting programmed death 1 and programmed death ligand 1,	https://doi.org/10.1016/j.biopha.2020.110621,
Targeting bile acid signaling for the treatment of liver diseases: From bench to bed,	https://doi.org/10.1016/j.biopha.2022.113154,
Multiple therapeutic targets in rare cholestatic liver diseases: Time to redefine treatment strategies,	https://doi.org/10.1016/j.aohep.2019.09.009,
Neuropsychiatric sequelae after liver transplantation and their possible mechanism via the microbiotaÃ¢â,¬â€œgutÃ¢â,¬â€œliverÃ¢â,¬â€œbrain axis,	https://doi.org/10.1016/j.biopha.2023.114855,
Bacteria-driven cancer therapy: Exploring advancements and challenges,	https://doi.org/10.1016/j.critrevonc.2023.104141,
Oxidative Stress and Redox-Modulating Therapeutics in Inflammatory Bowel Disease,	https://doi.org/10.1016/j.molmed.2020.06.006,
The potential of bacteriophage therapy in the treatment of paediatric respiratory infections,	https://doi.org/10.1016/j.prrv.2022.02.001,
Pathogenesis and treatment of depression: Role of diet in prevention and therapy,	https://doi.org/10.1016/j.nut.2023.112143,
The mechanism of intestinal microbiota regulating immunity and inflammation in ischemic stroke and the role of natural botanical active ingredients in regulating intestinal microbiota: A review,	https://doi.org/10.1016/j.biopha.2022.114026,
m6A modification in inflammatory bowel disease provides new insights into clinical applications,	https://doi.org/10.1016/j.biopha.2023.114298,
Dental implant surfaces and their interaction with the oral microbiome,	https://doi.org/10.1016/j.dentre.2022.100060,
When Rhythms Meet the Blues: Circadian Interactions with the Microbiota-Gut-Brain Axis,	https://doi.org/10.1016/j.cmet.2020.02.008,
Gut microbiota as an Ã¢â,¬Å“invisible organÃ¢â,¬Â that modulates the function of drugs,	https://doi.org/10.1016/j.biopha.2019.109653,

Oral and intestinal dysbiosis in Parkinson's disease,	https://doi.org/10.1016/j.neurol.2022.12.010,
A critical review of Astragalus polysaccharides: From therapeutic mechanisms to pharmaceutics,	https://doi.org/10.1016/j.biopha.2022.112654,
Pharmacokinetics/pharmacodynamics of phage therapy: a major hurdle to clinical translation,	https://doi.org/10.1016/j.cmi.2023.01.021,
TMAO: how gut microbiota contributes to heart failure,	https://doi.org/10.1016/j.trsl.2020.08.007,
Modulation of gut microbiota by bioactive compounds for prevention and management of type 2 diabetes,	https://doi.org/10.1016/j.biopha.2022.113148,
Possible role of nutrition in the prevention of inflammatory bowel diseaseÃ¢â,¬â€œrelated colorectal cancer: A focus on human studies,	https://doi.org/10.1016/j.nut.2023.111980,
The gut microbiota as a biomarker in epilepsy,	https://doi.org/10.1016/j.nbd.2021.105598,
The Effect of In Utero Exposure to Maternal Inflammatory Bowel Disease and Immunomodulators on Infant Immune System Development and Function,	https://doi.org/10.1016/j.jcmgh.2023.03.005,
Potential Biomarkers, Risk Factors, and Their Associations with IgE-Mediated Food Allergy in Early Life: A Narrative Review,	https://doi.org/10.1093/advances/nmab122,
A systematic review of natural products for skin applications: Targeting inflammation, wound healing, and photo-aging,	https://doi.org/10.1016/j.phymed.2023.154824,
The Role of Lung and Gut Microbiota in the Pathology of Asthma,	https://doi.org/10.1016/j.immuni.2020.01.007,
The Ã¢â,¬Å“CultureÃ¢â,¬Â of Pain Control: A Review of Opioid-Induced Dysbiosis (OID) in Antinociceptive Tolerance,	https://doi.org/10.1016/j.jpain.2019.11.015,
Immune Checkpoint Inhibitor Therapy inÃ,Â Oncology: Current Uses and Future Directions: JACC: CardioOncology State-of-the-Art Review,	https://doi.org/10.1016/j.jaccao.2022.09.004,
Association between the microbiota and womenÃ¢â,¬â„¢s cancers Ã¢â,¬â€œ Cause or consequences?,	https://doi.org/10.1016/j.biopha.2020.110203,
Diet-Induced HostÃ¢â,¬â€œMicrobe Interactions: Personalized Diet Strategies for Improving Inflammatory Bowel Disease,	https://doi.org/10.1093/cdn/nzac110,
Prognostic modelling in IBD,	https://doi.org/10.1016/j.bpg.2023.101877,
Impact of intestinal disorders on central and peripheral nervous system diseases,	https://doi.org/10.1016/j.nbd.2022.105627,
The role of gut microbiota in tumorigenesis and treatment,	https://doi.org/10.1016/j.biopha.2021.111444,
Gut morphology and gene expression in obesity: Short review and perspectives,	https://doi.org/10.1016/j.yclnex.2018.04.003,
Mediterranean diet adherence, gut microbiota, and Alzheimer's or Parkinson's disease risk: A systematic review,	https://doi.org/10.1016/j.jns.2022.120166,
The critical roles of iron during the journey from fetus to adolescent: Developmental aspects of iron homeostasis,	https://doi.org/10.1016/j.blre.2021.100866,

Body fatness associations with cancer: evidence from recent epidemiological studies and future directions,	https://doi.org/10.1016/j.metabol.2022.155326,
Clinical implications of nicotine as an antimicrobial agent and immune modulator,	https://doi.org/10.1016/j.biopha.2020.110404,
Pulmonary Manifestations of Inflammatory Bowel Disease and Treatment Strategies,	https://doi.org/10.1016/j.chpulm.2023.100018,
Gut microbiota and Autism Spectrum Disorder: From pathogenesis to potential therapeutic perspectives,	https://doi.org/10.1016/j.jtcme.2022.03.001,
Immunopathophysiology of human sepsis,	https://doi.org/10.1016/j.ebiom.2022.104363,
Plant polysaccharides utilized by gut microbiota: New players in ameliorating cognitive impairment,	https://doi.org/10.1016/j.jtcme.2022.01.003,
Chromatin dynamics and histone modifications in intestinal microbiota-host crosstalk,	https://doi.org/10.1016/j.molmet.2019.12.005,
Defining Interactions Between the Genome, Epigenome, and the Environment in Inflammatory Bowel Disease: Progress and Prospects,	https://doi.org/10.1053/j.gastro.2023.03.238,
Impaired Intestinal Sodium Transport in Inflammatory Bowel Disease: From the Passenger to the Driver's Seat,	https://doi.org/10.1016/j.jcmgh.2021.03.005,
The impact of microbiota-derived short-chain fatty acids on macrophage activities in disease: Mechanisms and therapeutic potentials,	https://doi.org/10.1016/j.biopha.2023.115276,
The exposome and liver disease - how environmental factors affect liver health,	https://doi.org/10.1016/j.jhep.2023.02.034,
Microfluidic Organ-on-a-Chip Models of Human Intestine,	https://doi.org/10.1016/j.jcmgh.2017.12.010,
Using integrated meta-omics to appreciate the role of the gut microbiota in epilepsy,	https://doi.org/10.1016/j.nbd.2022.105614,
Ginseng polysaccharides: Potential antitumor agents,	https://doi.org/10.1016/j.jgr.2022.07.002,
Beyond faecal microbiota transplantation, the non-negligible role of faecal virome or bacteriophage transplantation,	https://doi.org/10.1016/j.jmii.2023.02.005,
The Potential Cardiometabolic Effects of Long-Chain Ã â€°-3 Polyunsaturated Fatty Acids: Recent Updates and Controversies,	https://doi.org/10.1016/j.advnut.2023.03.014,
Current and emerging therapies for alcohol-associated hepatitis,	https://doi.org/10.1016/j.livres.2023.03.002,
HOTAIR in solid tumors: Emerging mechanisms and clinical strategies,	https://doi.org/10.1016/j.biopha.2022.113594,
The epithelial barrier: The gateway to allergic, autoimmune, and metabolic diseases and chronic neuropsychiatric conditions,	https://doi.org/10.1016/j.smim.2023.101846,
Microbial transformations of bile acids and their receptors in the regulation of metabolic dysfunction-associated steatotic liver disease,	https://doi.org/10.1016/j.livres.2023.09.002,
Therapeutic potential of melatonin in colorectal cancer: Focus on lipid metabolism and gut microbiota,	https://doi.org/10.1016/j.bbadis.2021.166281,
The microbiota in cirrhosis and its role in hepatic decompensation,	https://doi.org/10.1016/j.jhep.2020.11.013,

How to adapt an intestinal microbiota transplantation programme to reduce the risk of invasive multidrug-resistant infection,	https://doi.org/10.1016/j.cmi.2021.11.006,
Advances in mesenchymal stem cell-derived extracellular vesicles therapy for Sjogren's syndrome-related dry eye disease,	https://doi.org/10.1016/j.exer.2023.109716,
Molecular Aspects of Lifestyle and Environmental Effects in Patients WithÃ,Â Diabetes: JACCÃ,Â Focus Seminar,	https://doi.org/10.1016/j.jacc.2021.02.070,
Intervention strategies for microbial therapeutics in cancer immunotherapy,	https://doi.org/10.1016/j.iotech.2020.05.001,
Hidradenitis Suppurativa: Host-Microbe and Immune Pathogenesis Underlie Important Future Directions,	https://doi.org/10.1016/j.xjidi.2021.100001,
Mechanisms of Resistance and Treatment of Relapse after CAR T-cell Therapy for Large B-cell Lymphoma and Multiple Myeloma,	https://doi.org/10.1016/j.jtct.2023.04.007,
Regulation of host immune responses by Lactobacillus through aryl hydrocarbon receptors,	https://doi.org/10.1016/j.medmic.2023.100081,
The effect of probiotics on immune responses and their therapeutic application: A new treatment option for multiple sclerosis,	https://doi.org/10.1016/j.biopha.2022.114195,
Fecal microbiota transplantation in human metabolic diseases: From a murky past to a bright future?,	https://doi.org/10.1016/j.cmet.2021.05.005,
Small intestine vs. colon ecology and physiology: Why it matters in probiotic administration,	https://doi.org/10.1016/j.xcrm.2023.101190,
The role of microbiota in respiratory health and diseases, particularly in tuberculosis,	https://doi.org/10.1016/j.biopha.2021.112108,
Does Modification of the Large Intestinal Microbiome Contribute to the Anti-Inflammatory Activity of Fermentable Fiber?,	https://doi.org/10.3945/cdn.117.001180,
Exploring the potential impact of probiotic use on drug metabolism and efficacy,	https://doi.org/10.1016/j.biopha.2023.114468,
Forgotten fungi: the importance of the skin mycobiome,	https://doi.org/10.1016/j.mib.2022.102235,
Chronic fatigue and depression due to multiple sclerosis: Immune-inflammatory pathways, tryptophan catabolites and the gut-brain axis as possible shared pathways,	https://doi.org/10.1016/j.msard.2020.102533,
Ulcerative colitis: Gut microbiota, immunopathogenesis and application of natural products in animal models,	https://doi.org/10.1016/j.lfs.2020.118129,
Mechanisms for control of skin immune function by the microbiome,	https://doi.org/10.1016/j.coi.2021.09.001,
Diet and the MicrobiotaÃ¢â,¬â€œGutÃ¢â,¬â€œBrain Axis: Sowing the Seeds of Good Mental Health,	https://doi.org/10.1093/advances/nmaa181,
Long term management of ulcerative colitis with Faecal Microbiota Transplantation,	https://doi.org/10.1016/j.medmic.2020.100026,
Portal hypertension in cirrhosis: Pathophysiological mechanisms and therapy,	https://doi.org/10.1016/j.jhepr.2021.100316,
Microbiologic factors affecting Clostridium difficile recurrence,	https://doi.org/10.1016/j.cmi.2017.11.017,

Role of mesenchymal stem cells and short chain fatty acids in allergy: A prophylactic therapy for future,	https://doi.org/10.1016/j.imlet.2023.06.002,
Host-Gut Microbiota Crosstalk in Intestinal Adaptation,	https://doi.org/10.1016/j.jcmgh.2018.01.024,
Intestinal mucus barrier: a missing piece of the puzzle in food allergy,	https://doi.org/10.1016/j.molmed.2021.10.004,
25 Years of translational research in the Copenhagen Prospective Studies on Asthma in Childhood (COPSAC),	https://doi.org/10.1016/j.jaci.2022.11.022,
Shaping the Future of Probiotics and Prebiotics,	https://doi.org/10.1016/j.tim.2021.01.003,
Cytokine Modulators in Plaque PsoriasisÃ‚Â Ã¢â‚¬â€œÃ‚Â A Review of Current and Prospective Biologic Therapeutic Approaches,	https://doi.org/10.1016/j.jdin.2022.08.008,
Indoles as essential mediators in the gut-brain axis. Their role in Alzheimer's disease,	https://doi.org/10.1016/j.nbd.2021.105403,
Microbial mismanagement: how inadequate treatments for vaginal dysbiosis drive the HIV epidemic in women,	https://doi.org/10.1016/j.smim.2021.101482,
Targeting Helicobacter pylori for antibacterial drug discovery with novel therapeutics,	https://doi.org/10.1016/j.mib.2022.102203,
Updated Management of Colorectal Cancer Liver Metastases: Scientific Advances Driving Modern Therapeutic Innovations,	https://doi.org/10.1016/j.jcmgh.2023.08.012,
Predictive Biomarkers for Immunotherapy in Lung Cancer: Perspective From the International Association for the Study of Lung Cancer Pathology Committee,	https://doi.org/10.1016/j.jtho.2022.09.109,
Macrophages in the gut: Masters in multitasking,	https://doi.org/10.1016/j.immuni.2022.08.005,
Carcinogenesis as a Result of Multiple Inflammatory and Oxidative Hits: a Comprehensive Review from Tumor Microenvironment to Gut Microbiota,	https://doi.org/10.1016/j.neo.2018.05.002,
Dysregulation of the gut-brain-skin axis and key overlapping inflammatory and immune mechanisms of psoriasis and depression,	https://doi.org/10.1016/j.biopha.2020.111065,
Where is bone science taking us?,	https://doi.org/10.1016/j.berh.2022.101791,
Endotypes in bronchiectasis: moving towards precision medicine. A narrative review,	https://doi.org/10.1016/j.pulmoe.2023.03.004,
Gut microbiota-derived short-chain fatty acids and hypertension: Mechanism and treatment,	https://doi.org/10.1016/j.biopha.2020.110503,
Role of microbiota short-chain fatty acids in the pathogenesis of autoimmune diseases,	https://doi.org/10.1016/j.biopha.2023.114620,
Psychedelics as a novel approach to treating autoimmune conditions,	https://doi.org/10.1016/j.imlet.2020.10.001,
Reframing anorexia nervosa as a metabo-psychiatric disorder,	https://doi.org/10.1016/j.tem.2021.07.010,
Metabolite interactions between host and microbiota during health and disease: Which feeds the other?,	https://doi.org/10.1016/j.biopha.2023.114295,
The forgotten oral microbial transplantation for improving the outcomes of COVID-19,	https://doi.org/10.1016/j.nmni.2021.100923,
Mechanisms of Action and Therapeutic Application of Glucagon-like Peptide-1,	https://doi.org/10.1016/j.cmet.2018.03.001,

Sex and gender perspectives in colorectal cancer,	https://doi.org/10.1016/j.esmoop.2023.101204,
Antigenic mimicry Ã¢â,¬â€œ The key to autoimmunity in immune privileged organs,	https://doi.org/10.1016/j.jaut.2022.102942,
Postbiotics as potential new therapeutic agents for metabolic disorders management,	https://doi.org/10.1016/j.biopha.2022.113138,
Exosomes in Food: Health Benefits and Clinical Relevance in Diseases,	https://doi.org/10.1093/advances/nmz123,
An update on potential biomarkers for diagnosing diabetic foot ulcer at early stage,	https://doi.org/10.1016/j.biopha.2020.110991,
Alternatives to antibiotics in a One Health context and the role genomics can play in reducing antimicrobial use,	https://doi.org/10.1016/j.cmi.2020.02.028,
Noninvasive, MultiOmic, and Multicompartmental Biomarkers of Reflux Disease: A Systematic Review,	https://doi.org/10.1016/j.gastha.2023.01.014,
A systematic review of potential candidates of herbal medicine in treatment of chronic kidney disease,	https://doi.org/10.1016/j.phyplu.2022.100361,
Proton Pump Inhibitors and Cognitive Health: Review on Unraveling the Dementia Connection and Co-morbid Risks,	https://doi.org/10.2174/011567205028994624022305 0737,
IBD metabonomics predicts phenotype, disease course, and treatment response,	https://doi.org/10.1016/j.ebiom.2021.103551,
Challenges in tackling energy expenditure as obesity therapy: From preclinical models to clinical application,	https://doi.org/10.1016/j.molmet.2021.101237,
Melanoma models for the next generation of therapies,	https://doi.org/10.1016/j.ccell.2021.01.011,
Machine learning for metabolomics research in drug discovery,	https://doi.org/10.1016/j.ibmed.2023.100101,
Update and review of the gerodontology prospective for 2020's: Linking the interactions of oral (hypo)-functions to health vs. systemic diseases,	https://doi.org/10.1016/j.jds.2020.09.007,
Systematic review of donor and recipient predictive biomarkers of response to faecal microbiota transplantation in patients with ulcerative colitis,	https://doi.org/10.1016/j.ebiom.2022.104088,
Microbial Dynamics in Newly Diagnosed and Treatment Naïve IBD Patients in the Mediterranean.	https://doi.org/10.1093/ibd/izad004,
Osteoarthritis year in review 2020: biology,	https://doi.org/10.1016/j.joca.2020.10.006,
Primary and secondary immune checkpoint inhibitors resistance in colorectal cancer: Key mechanisms and ways to overcome resistance,	https://doi.org/10.1016/j.ctarc.2022.100643,
Refining patient selection for breast cancer immunotherapy: beyond PD-L1,	https://doi.org/10.1016/j.esmoop.2021.100257,
Disco interacting protein 2 homolog A (DIP2A): A key component in the regulation of brain disorders,	https://doi.org/10.1016/j.biopha.2023.115771,
Salt Reduction to Prevent Hypertension and Cardiovascular Disease: JACC State-of-the-Art Review,	https://doi.org/10.1016/j.jacc.2019.11.055,
Understanding the cellular interactome of non-alcoholic fatty liver disease,	https://doi.org/10.1016/j.jhepr.2022.100524,
Polycystic ovarian syndrome-current pharmacotherapy and clinical implications,	https://doi.org/10.1016/j.tjog.2021.11.009,

Genomics, microbiomics, proteomics, and metabolomics in bronchopulmonary dysplasia,	https://doi.org/10.1053/j.semperi.2018.09.004,
Long COVID: pathophysiological factors and abnormalities of coagulation,	https://doi.org/10.1016/j.tem.2023.03.002,
Probiotic applications associated with Psyllium fiber as prebiotics geared to a healthy intestinal microbiota: A review,	https://doi.org/10.1016/j.nut.2022.111772,
Evolution of Plasmid-Mediated Antibiotic Resistance in the Clinical Context,	https://doi.org/10.1016/j.tim.2018.06.007,
Carotenoids in Health as Studied by Omics-Related Endpoints,	https://doi.org/10.1016/j.advnut.2023.09.002,
Synthetic microbiology applications powered by light,	https://doi.org/10.1016/j.mib.2022.102158,
Pathological features-based targeted delivery strategies in IBD therapy: A mini review,	https://doi.org/10.1016/j.biopha.2022.113079,
The Role of Race, Sex, and Age in Circadian Disruption and Metabolic Disorders,	https://doi.org/10.1016/j.gastha.2022.02.015,
Genetic predisposition similarities between NASH and ASH: Identification of new therapeutic targets,	https://doi.org/10.1016/j.jhepr.2021.100284,
Topical Microbial Therapeutics against Respiratory Viral Infections,	https://doi.org/10.1016/j.molmed.2021.03.009,
Conquering rheumatic diseases: are parasitic worms the answer?,	https://doi.org/10.1016/j.pt.2023.06.010,
Role of Bifidobacterium in Modulating the Intestinal Epithelial Tight Junction Barrier: Current Knowledge and Perspectives,	https://doi.org/10.1016/j.cdnut.2023.102026,
Nutritional and dietary strategy in the clinical care of inflammatory bowel disease,	https://doi.org/10.1016/j.jfma.2019.09.005,
Post-COVID-19 syndrome management: Utilizing the potential of dietary polysaccharides,	https://doi.org/10.1016/j.biopha.2023.115320,
Gaps and opportunities in sepsis translational research,	https://doi.org/10.1016/j.ebiom.2022.104387,
Microbiota and body weight control: Weight watchers within?,	https://doi.org/10.1016/j.molmet.2021.101427,
Environmental triggers of Parkinson's disease Ã¢â,¬â€œ Implications of the Braak and dual-hit hypotheses,	https://doi.org/10.1016/j.nbd.2021.105601,
Agrochemicals and obesity,	https://doi.org/10.1016/j.mce.2020.110926,
Behavioral approaches to nutrition and eating patterns for managing type 2 diabetes: A review,	https://doi.org/10.1016/j.ajmo.2023.100034,
Dietary and Microbial Determinants in Food Allergy,	https://doi.org/10.1016/j.immuni.2020.07.025,
Biobehavioral Research and Hematopoietic Stem Cell Transplantation: Expert Review from the Biobehavioral Research Special Interest Group of the American Society for Transplantation and Cellular Therapy,	https://doi.org/10.1016/j.jtct.2021.06.007,
Exosome-based regenerative rehabilitation: A novel ice breaker for neurological disorders,	https://doi.org/10.1016/j.biopha.2023.115920,
Negative regulatory NLRs mitigate inflammation via NF-ÃŽÂºB pathway signaling in inflammatory bowel disease,	https://doi.org/10.1016/j.bj.2023.100616,
Chronic GvHD NIH Consensus Project Biology Task Force: evolving path to personalized treatment of chronic GvHD,	https://doi.org/10.1182/bloodadvances.2022007611,

Microbiota-derived short-chain fatty acids functions in the biology of B lymphocytes: From differentiation to antibody formation,	https://doi.org/10.1016/j.biopha.2023.115773,
New biomarkers for checkpoint inhibitor therapy,	https://doi.org/10.1136/esmoopen-2019-000597,
Immune-mediated inflammatory diseases: Common and different pathogenic and clinical features,	https://doi.org/10.1016/j.autrev.2023.103410,
Breastfeeding and Medication Use in Kidney Disease,	https://doi.org/10.1053/j.ackd.2020.05.007,
Microbiota-derived short-chain fatty acids and modulation of host-derived peptides formation: Focused on host defense peptides,	https://doi.org/10.1016/j.biopha.2023.114586,
Emerging mechanisms of valproic acid-induced neurotoxic events in autism and its implications for pharmacological treatment,	https://doi.org/10.1016/j.biopha.2021.111322,
Unraveling the function of epithelial-mesenchymal transition (EMT) in colorectal cancer: Metastasis, therapy response, and revisiting molecular pathways,	https://doi.org/10.1016/j.biopha.2023.114395,
Addressing antibiotic resistance: computational answers to a biological problem?,	https://doi.org/10.1016/j.mib.2023.102305,
Towards promising antimicrobial alternatives: The future of bacteriophage research and development in Saudi Arabia,	https://doi.org/10.1016/j.jiph.2022.10.022,
Applying Early Intervention Strategies to Autoimmune Skin Diseases. Is the Window of Opportunity Preclinical? A Dermato-Rheumatology Perspective,	https://doi.org/10.1016/j.jid.2021.11.018,
Immune Checkpoint Inhibitors in Thoracic Malignancies: Review of the Existing Evidence by an IASLC Expert Panel and Recommendations,	https://doi.org/10.1016/j.jtho.2020.03.006,
Immunopathogenesis of BehÃƒÂ§et's disease and treatment modalities,	https://doi.org/10.1016/j.semarthrit.2022.151956,
Update of treatment algorithms for Clostridium difficile infection,	https://doi.org/10.1016/j.cmi.2017.12.022,
Evaluation of anti-TNF therapeutic response in patients with inflammatory bowel disease: Current and novel biomarkers,	https://doi.org/10.1016/j.ebiom.2021.103329,
Biological ageing with HIV infection: evaluating the geroscience hypothesis,	https://doi.org/10.1016/S2666-7568(21)00278-6,
Modes of therapeutic delivery in synthetic microbiology,	https://doi.org/10.1016/j.tim.2022.09.003,
The microbiota and radiotherapy for head and neck cancer: What should clinical oncologists know?,	https://doi.org/10.1016/j.ctrv.2022.102442,
Inflammatory Bowel Disease and Atherosclerotic Cardiovascular Disease: JACC Review Topic of the Week,	https://doi.org/10.1016/j.jacc.2020.10.027,
The 2020 BMT CTN Myeloma Intergroup Workshop on Immune Profiling and Minimal Residual Disease Testing in Multiple Myeloma,	https://doi.org/10.1016/j.jtct.2021.05.027,
Role of microbiota-derived short-chain fatty acids in cancer development and prevention,	https://doi.org/10.1016/j.biopha.2021.111619,

Research hotspot and frontier analysis of traditional Chinese medicine in asthma using bibliometric methods from 1991 to 2021,	https://doi.org/10.1016/j.jacig.2022.07.004,
The role and mechanism of action of microbiota-derived short-chain fatty acids in neutrophils: From the activation to becoming potential biomarkers,	https://doi.org/10.1016/j.biopha.2023.115821,
Lactoferrin: Antimicrobial impacts, genomic guardian, therapeutic uses and clinical significance for humans and animals,	https://doi.org/10.1016/j.biopha.2023.114967,
Intestinal microbiota: A bridge between intermittent fasting and tumors,	https://doi.org/10.1016/j.biopha.2023.115484,
Immune-related adverse events in various organs caused by immune checkpoint inhibitors,	https://doi.org/10.1016/j.alit.2022.01.001,
Integration of androgen hormones in endometrial cancer biology,	https://doi.org/10.1016/j.tem.2022.06.001,
Epigenetics, microbiota, and intraocular inflammation: New paradigms of immune regulation in the eye,	https://doi.org/10.1016/j.preteyeres.2018.01.001,
Immune dysregulation,	https://doi.org/10.1016/j.jaci.2022.11.001,
The molecular mechanisms that underpin the biological benefits of full-spectrum cannabis extract in the treatment of neuropathic pain and inflammation,	https://doi.org/10.1016/j.bbadis.2020.165771,
Nutritional Supplements and Complementary Therapies in Polycystic Ovary Syndrome,	https://doi.org/10.1093/advances/nmab141,
Challenges of Studying the Human Virome Ã¢â,¬â€œ Relevant Emerging Technologies,	https://doi.org/10.1016/j.tim.2020.05.021,
Retinoid-Related Orphan Receptor RORÃŽÂ³t in CD4+ T-CellÃ¢â,¬â€œMediated Intestinal Homeostasis and Inflammation,	https://doi.org/10.1016/j.ajpath.2020.07.010,
Role of microbiota-derived short-chain fatty acids in nervous system disorders,	https://doi.org/10.1016/j.biopha.2021.111661,
Natural products targeting Nrf2/ARE signaling pathway in the treatment of inflammatory bowel disease,	https://doi.org/10.1016/j.biopha.2023.114950,
Nutrition, dietary recommendations, and supplements for patients with congenital heart disease,	https://doi.org/10.1016/j.ijcchd.2023.100449,
Sex and Gender in Glomerular Disease,	https://doi.org/10.1016/j.semnephrol.2022.04.008,
The Intestinal Microbiota in Colorectal Cancer,	https://doi.org/10.1016/j.ccell.2018.03.004,
The pathogenesis of rheumatoid arthritis,	https://doi.org/10.1016/j.immuni.2022.11.009,
Inflammasome Signaling in AtrialÃ,Â Fibrillation: JACC State-of-the-Art Review,	https://doi.org/10.1016/j.jacc.2022.03.379,
Diet- and microbiota-related metabolite, 5-aminovaleric acid betaine (5-AVAB), in health and disease,	https://doi.org/10.1016/j.tem.2022.04.004,
Current advances on the therapeutic potential of pinocembrin: An updated review,	https://doi.org/10.1016/j.biopha.2022.114032,
Constipation in DM are associated with both poor glycemic control and diabetic complications: Current status and future directions,	https://doi.org/10.1016/j.biopha.2023.115202,
Anti-TIGIT therapies for solid tumors: a systematic review,	https://doi.org/10.1016/j.esmoop.2023.101184,

Integrating ayurvedic medicine into cancer research programs part 1: Ayurveda background and applications,	https://doi.org/10.1016/j.jaim.2022.100676,
Painful interactions: Microbial compounds and visceral pain,	https://doi.org/10.1016/j.bbadis.2019.165534,
HIV, Combination Antiretroviral Therapy, and Vascular Diseases in Men and Women,	https://doi.org/10.1016/j.jacbts.2021.10.017,
Novel potential treatable traits in asthma: Where is the research taking us?,	https://doi.org/10.1016/j.jacig.2022.04.001,
Dichotomous colorectal cancer behaviour,	https://doi.org/10.1016/j.critrevonc.2023.104067,
The bidirectional risk of inflammatory bowel disease and anxiety or depression: A systematic review and meta-analysis,	https://doi.org/10.1016/j.genhosppsych.2023.05.002,
Menstruation: science and society,	https://doi.org/10.1016/j.ajog.2020.06.004,
Inositols and metabolic disorders: From farm to bedside,	https://doi.org/10.1016/j.jtcme.2020.03.005,
Environmental influences on clonal hematopoiesis,	https://doi.org/10.1016/j.exphem.2019.12.005,
Current and emerging therapies for first-line treatment of metastatic clear cell renal cell carcinoma,	https://doi.org/10.1016/j.ctrv.2018.07.009,
Systematic review on fecal calprotectin in cystic fibrosis,	https://doi.org/10.1016/j.jped.2022.01.006,
Metabolomics as a promising tool for improving understanding of multiple sclerosis: A review of recent advances,	https://doi.org/10.1016/j.bj.2022.01.004,
Hypotension as a marker or mediator of perioperative organ injury: a narrative review,	https://doi.org/10.1016/j.bja.2022.01.012,
COVID-19 infection and metabolic comorbidities: Mitigating role of nutritional sufficiency and drug Ã¢â‚¬â€œ nutraceutical combinations of vitamin D,	https://doi.org/10.1016/j.hnm.2022.200179,
Multi-omics profiling approach in food allergy,	https://doi.org/10.1016/j.waojou.2023.100777,
Focus on immune checkpoint PD-1/PD-L1 pathway: New advances of polyphenol phytochemicals in tumor immunotherapy,	https://doi.org/10.1016/j.biopha.2022.113618,
Immune determinants of the pre-metastatic niche,	https://doi.org/10.1016/j.ccell.2023.02.018,
Dirty mice join the immunologist's toolkit,	https://doi.org/10.1016/j.micinf.2021.104817,
Circular RNAs in depression: Biogenesis, function, expression, and therapeutic potential,	https://doi.org/10.1016/j.biopha.2021.111244,
Anti-TSLP antibodies: Targeting a master regulator of type 2 immune responses,	https://doi.org/10.1016/j.alit.2020.01.001,
Interleukin-1 (IL-1) and the inflammasome in cancer,	https://doi.org/10.1016/j.cyto.2022.155850,
Thrombosis: Current knowledge based on metabolomics by nuclear magnetic resonance (NMR) spectroscopy and mass spectrometry (MS),	https://doi.org/10.1016/j.tru.2020.100011,
Mechanism and potential predictive biomarkers of immune checkpoint inhibitors in NSCLC,	https://doi.org/10.1016/j.biopha.2020.109996,
Diversity-Generating Machines: Genetics of Bacterial Sugar-Coating,	https://doi.org/10.1016/j.tim.2018.06.006,
Data-Driven Modeling of Pregnancy-Related Complications,	https://doi.org/10.1016/j.molmed.2021.01.007,

Opinions on the current pandemic of COVID-19: Use functional food to boost our immune functions,	https://doi.org/10.1016/j.jiph.2020.08.014,
CTLA-4: a moving target in immunotherapy,	https://doi.org/10.1182/blood-2017-06-741033,
Dietary influence on central nervous system myelin production, injury, and regeneration,	https://doi.org/10.1016/j.bbadis.2020.165779,
Etiopathogenetic Mechanisms in Diverticular Disease of the Colon,	https://doi.org/10.1016/j.jcmgh.2019.07.007,
Role of Interleukin-22 in ulcerative colitis,	https://doi.org/10.1016/j.biopha.2023.114273,
The need for Africa to develop capacity for vaccinology as a means of curbing antimicrobial resistance,	https://doi.org/10.1016/j.jvacx.2023.100320,
Chronic inflammation and the hallmarks of aging,	https://doi.org/10.1016/j.molmet.2023.101755,
Biofilm modifiers: The disparity in paradigm of oral biofilm ecosystem,	https://doi.org/10.1016/j.biopha.2023.114966,
Leveraging Human Genetics to Identify Potential New Treatments for Fatty Liver Disease,	https://doi.org/10.1016/j.cmet.2019.12.002,
Plant compounds for obesity treatment through neuroendocrine regulation of hunger: A systematic review,	https://doi.org/10.1016/j.phymed.2023.154735,
Single-cell multiomics in neuroinflammation,	https://doi.org/10.1016/j.coi.2022.102180,
Regulation, risk and safety of Faecal Microbiota Transplant,	https://doi.org/10.1016/j.infpip.2020.100069,
Roles and mechanisms of quercetin on cardiac arrhythmia: A review,	https://doi.org/10.1016/j.biopha.2022.113447,
Medicinal uses, pharmacological activities, phytochemistry, and the molecular mechanisms of Punica granatum L. (pomegranate) plant extracts: A review,	https://doi.org/10.1016/j.biopha.2022.113256,
Inflammation in HeartÃ‚Â Failure: JACC State-of-the-Art Review,	https://doi.org/10.1016/j.jacc.2020.01.014,
How to: prophylactic interventions for prevention of Clostridioides difficile infection,	https://doi.org/10.1016/j.cmi.2021.06.037,
The immunology of sepsis,	https://doi.org/10.1016/j.immuni.2021.10.012,
The potential mechanism of postoperative cognitive dysfunction in older people,	https://doi.org/10.1016/j.exger.2019.110791,
Sand Fly Studies Predict Transmission Potential of Drug-resistant Leishmania,	https://doi.org/10.1016/j.pt.2020.06.006,
Pathogenesis of Fistulating CrohnÃ¢â‚¬â„¢s Disease: A Review,	https://doi.org/10.1016/j.jcmgh.2022.09.011,
Vaccination in liver diseases and liver transplantation: Recommendations, implications and opportunities in the post-COVID era,	https://doi.org/10.1016/j.jhepr.2023.100776,
Saliva diagnostics: Salivaomics, saliva exosomics, and saliva liquid biopsy,	https://doi.org/10.1016/j.adaj.2023.05.006,
The Ã¢â‚¬Å“Treatise on the spleen and stomachÃ¢â‚¬Â (PÃƒÂ WÃƒÂ¨i LÃƒÂ¹n) as the first record of multiple sclerosis in the medical literature Ã¢â‚¬â€œ A hypothesis based on the analysis of clinical presentation and herbal medicine,	https://doi.org/10.1016/j.jtcme.2020.02.009,
Herbal medications and natural products for patients with covid-19 and diabetes mellitus: Potentials and challenges,	https://doi.org/10.1016/j.phyplu.2022.100280,

A glimpse at the metabolic research in China,	https://doi.org/10.1016/j.cmet.2021.09.014,
Recent insights into the role of defensins in diabetic wound healing,	https://doi.org/10.1016/j.biopha.2022.113694,
Recent developments and advances in atopic dermatitis and food allergy,	https://doi.org/10.1016/j.alit.2019.08.013,
Histone/protein deacetylase inhibitor therapy for enhancement of Foxp3+ T-regulatory cell function posttransplantation,	https://doi.org/10.1111/ajt.14749,
Microbiota-derived short chain fatty acids: Their role and mechanisms in viral infections,	https://doi.org/10.1016/j.biopha.2023.114414,
Chronic Kidney disease and stroke: A Bi-directional risk cascade and therapeutic update,	https://doi.org/10.1016/j.dscb.2021.100017,
Role of SARS-COV-2 and ACE2 in the pathophysiology of peripheral vascular diseases,	https://doi.org/10.1016/j.biopha.2023.115321,
Nutrition state of science and dementia prevention: recommendations of the Nutrition for Dementia Prevention Working Group,	https://doi.org/10.1016/S2666-7568(22)00120-9,
The search for disease-modifying agents in decompensated cirrhosis: From drug repurposing to drug discovery,	https://doi.org/10.1016/j.jhep.2021.01.024,
The etiology of rheumatoid arthritis,	https://doi.org/10.1016/j.jaut.2019.102400,
Systemic antibiotics adjuvants to scaling and root planing in type 2 diabetic and periodontitis individuals: Systematic review with network meta-analysis,	https://doi.org/10.1016/j.jdsr.2023.06.001,
Methodology, efficacy and safety of fecal microbiota transplantation in treating inflammatory bowel disease,	https://doi.org/10.1016/j.medmic.2020.100028,
What Is the Value of Counting Mast Cells in Gastrointestinal Mucosal Biopsies?,	https://doi.org/10.1016/j.modpat.2022.100005,
Nature versus nurture in the spectrum of rheumatic diseases: Classification of spondyloarthritis as autoimmune or autoinflammatory,	https://doi.org/10.1016/j.autrev.2018.04.002,
Artificial intelligence, nutrition, and ethical issues: A mini-review,	https://doi.org/10.1016/j.nutos.2023.07.001,
Old age as a risk factor for liver diseases: Modern therapeutic approaches,	https://doi.org/10.1016/j.exger.2023.112334,
Bacterial vaginosis: A state of microbial dysbiosis,	https://doi.org/10.1016/j.medmic.2023.100082,
Proceeding report of the Fourth Symposium on Hidradenitis Suppurativa Advances 2019,	https://doi.org/10.1016/j.jaad.2020.05.114,
Flavonoids against the SARS-CoV-2 induced inflammatory storm,	https://doi.org/10.1016/j.biopha.2021.111430,
Nucleic acid biomarkers to assess graft injury after liver transplantation,	https://doi.org/10.1016/j.jhepr.2022.100439,
TFOS Lifestyle Report Executive Summary: A Lifestyle Epidemic - Ocular Surface Disease,	https://doi.org/10.1016/j.jtos.2023.08.009,
Pathophysiological and clinical point of view on Kawasaki disease and MIS-C,	https://doi.org/10.1016/j.pedneo.2023.05.002,
The role of cannabidiol in aging,	https://doi.org/10.1016/j.biopha.2023.115074,
Integrating ayurvedic medicine into cancer research programs part 2: Ayurvedic herbs and research opportunities,	https://doi.org/10.1016/j.jaim.2022.100677,

Antibiotic resistance in microbes: History, mechanisms, therapeutic strategies and future prospects,	https://doi.org/10.1016/j.jiph.2021.10.020,
Global colorectal cancer burden in 2020 and projections to 2040,	https://doi.org/10.1016/j.tranon.2021.101174,
The cancer-immunity cycle: Indication, genotype, and immunotype,	https://doi.org/10.1016/j.immuni.2023.09.011,
Targeted lactate dehydrogenase genes silencing in probiotic lactic acid bacteria: A possible paradigm shift in colorectal cancer treatment?,	https://doi.org/10.1016/j.biopha.2023.114371,
Inflammation, Aging, and CardiovascularÃ‚Â Disease: JACC Review Topic of the Week,	https://doi.org/10.1016/j.jacc.2021.12.017,
Comparison of immune checkpoint inhibitor-induced arthritis and reactive arthritis to inform therapeutic strategy,	https://doi.org/10.1016/j.biopha.2022.112687,
New insights into MAIT cells in autoimmune diseases,	https://doi.org/10.1016/j.biopha.2023.114250,
Dissecting skin microbiota and microenvironment for the development of therapeutic strategies,	https://doi.org/10.1016/j.mib.2023.102311,
The potential of rapalogs to enhance resilience against SARS-CoV-2 infection and reduce the severity of COVID-19,	https://doi.org/10.1016/S2666-7568(20)30068-4,
Biobanking in dentistry: A review,	https://doi.org/10.1016/j.jdsr.2021.12.002,
Reversing memory/cognitive impairment with medicinal plants targeting inflammation and its crosstalk with other pathologies,	https://doi.org/10.1016/j.dscb.2023.100094,
Sex Differences Across the Life Course: A Focus On Unique Nutritional and Health Considerations among Women,	https://doi.org/10.1093/jn/nxac059,
Structural racism and its pathways to asthma and atopic dermatitis,	https://doi.org/10.1016/j.jaci.2021.09.020,
Type II diabetes mellitus: a review on recent drug based therapeutics,	https://doi.org/10.1016/j.biopha.2020.110708,
A critical review on the relationship of herbal medicine, Akkermansia muciniphila, and human health,	https://doi.org/10.1016/j.biopha.2020.110352,
Preclinical and clinical studies of immunotherapy for the treatment of cholangiocarcinoma,	https://doi.org/10.1016/j.jhepr.2023.100723,
Combined alcoholic and non-alcoholic steatohepatitis,	https://doi.org/10.1016/j.jhepr.2020.100101,
cAMP-PKA cascade: An outdated topic for depression?,	https://doi.org/10.1016/j.biopha.2022.113030,
The Combiome Hypothesis: Selecting Optimal Treatment for Cancer Patients,	https://doi.org/10.1016/j.cllc.2021.08.011,
Clinical challenges of short bowel syndrome and the path forward for organoid-based regenerative medicine,	https://doi.org/10.1016/j.reth.2023.06.001,
From gene identifications to therapeutic targets for asthma: Focus on great potentials of TSLP, ORMDL3, and GSDMB,	https://doi.org/10.1016/j.pccm.2023.08.001,
AGA-PancreasFest Joint Symposium on Exocrine Pancreatic Insufficiency,	https://doi.org/10.1016/j.gastha.2022.11.008,

Moderating Ã¢â,¬Å“the great debateÃ¢â,¬Â : The carbohydrate-insulin vs. the energy balance models of obesity,	https://doi.org/10.1016/j.cmet.2023.03.020,
An overview on role of nutrition on COVID-19 immunity: Accumulative review from available studies,	https://doi.org/10.1016/j.nutos.2022.11.001,
Emerging roles for HMGA2 in colorectal cancer,	https://doi.org/10.1016/j.tranon.2020.100894,
Immunomodulatory roles of microbiota-derived short-chain fatty acids in bacterial infections,	https://doi.org/10.1016/j.biopha.2021.111817,
Kynurenine pathway in Parkinson's diseaseÃ¢â,¬â€ An update,	https://doi.org/10.1016/j.ensci.2020.100270,
Meta-hallmarks of aging and cancer,	https://doi.org/10.1016/j.cmet.2022.11.001,
Usage, biological activity, and safety of selected botanical dietary supplements consumed in the United States,	https://doi.org/10.1016/j.jtcme.2018.01.006,
Supplementation of Bovine Colostrum in Inflammatory Bowel Disease: Benefits and Contraindications,	https://doi.org/10.1093/advances/nmaa120,
Engineering therapeutic phages for enhanced antibacterial efficacy,	https://doi.org/10.1016/j.coviro.2021.12.003,
Therapeutic effect of berberine on metabolic diseases: Both pharmacological data and clinical evidence,	https://doi.org/10.1016/j.biopha.2020.110984,
COVID-19 and inflammatory bowel disease: A pathophysiological assessment,	https://doi.org/10.1016/j.biopha.2021.111233,
Novel epigenetic therapeutic strategies and targets in cancer,	https://doi.org/10.1016/j.bbadis.2022.166552,
Investigating Ketone Bodies as Immunometabolic Countermeasures against Respiratory Viral Infections,	https://doi.org/10.1016/j.medj.2020.06.008,
Heterogeneous population of macrophages in the development of non-alcoholic fatty liver disease,	https://doi.org/10.1016/j.livres.2022.06.001,
Fracture healing research: Recent insights,	https://doi.org/10.1016/j.bonr.2023.101686,
Diet for the prevention and management of sarcopenia,	https://doi.org/10.1016/j.metabol.2023.155637,
Anti-inflammatory phytochemicals for the treatment of diabetes and its complications: Lessons learned and future promise,	https://doi.org/10.1016/j.biopha.2020.110975,
Cardiovascular and haematological pathology in myalgic encephalomyelitis/chronic fatigue syndrome (ME/CFS): A role for viruses,	https://doi.org/10.1016/j.blre.2023.101075,
Neuroimaging perspective in targeted treatment for type 2 diabetes melitus and sleep disorders,	https://doi.org/10.1016/j.imed.2022.05.003,
Concepts and Applications of Information Theory to Immuno-Oncology,	https://doi.org/10.1016/j.trecan.2020.12.013,
Luteolin as a potential hepatoprotective drug: Molecular mechanisms and treatment strategies,	https://doi.org/10.1016/j.biopha.2023.115464,
Organoids and organ chips in ophthalmology,	https://doi.org/10.1016/j.jtos.2020.11.004,
Therapeutic approaches in the treatment of gout,	https://doi.org/10.1016/j.semarthrit.2020.04.010,
Evolving concepts in NAD+ metabolism,	https://doi.org/10.1016/j.cmet.2021.04.003,

Future medicine: from molecular pathways to the collective intelligence of the body,	https://doi.org/10.1016/j.molmed.2023.06.007,
Aging-associated immune system changes in multiple myeloma: The dark side of the moon.,	https://doi.org/10.1016/j.ctarc.2021.100494,
The spontaneous remission of cancer: Current insights and therapeutic significance,	https://doi.org/10.1016/j.tranon.2021.101166,
Nutritional Genomics and Direct-to-Consumer Genetic Testing: An Overview,	https://doi.org/10.1093/advances/nmy001,
T follicular helper cells in cancer,	https://doi.org/10.1016/j.trecan.2022.12.007,
Immune-mediated enteropathies: From bench to bedside,	https://doi.org/10.1016/j.jaut.2021.102609,
Protein losing enteropathy after the Fontan operation,	https://doi.org/10.1016/j.ijcchd.2022.100338,
Insulin resistance and insulin sensitizing agents,	https://doi.org/10.1016/j.metabol.2021.154892,
Prevention of cardiovascular disease in young adults: Focus on gender differences. A collaborative review from the EAS Young Fellows,	https://doi.org/10.1016/j.atherosclerosis.2023.117272 ,
The World Goes Bats: Living Longer and Tolerating Viruses,	https://doi.org/10.1016/j.cmet.2020.06.013,
Emerging application of metabolomics on Chinese herbal medicine for depressive disorder,	https://doi.org/10.1016/j.biopha.2021.111866,
Is There a Causal Link Between Periodontitis and Cardiovascular Disease? A Concise Review of Recent Findings,	https://doi.org/10.1016/j.identj.2021.07.006,
Evaluating the beneficial effects of dietary restrictions: A framework for precision nutrigeroscience,	https://doi.org/10.1016/j.cmet.2021.08.018,
Antibiotic use in food animals worldwide, with a focus on Africa: Pluses and minuses,	https://doi.org/10.1016/j.jgar.2019.07.031,
Exercise, redox system and neurodegenerative diseases,	https://doi.org/10.1016/j.bbadis.2020.165778,
WhatÃ¢â‚¬â„¢s new in trauma 2020,	https://doi.org/10.1016/j.cjtee.2021.02.001,
A Road Map of the Axial Spondyloarthritis Continuum,	https://doi.org/10.1016/j.mayocp.2021.08.007,
The role of fatty acids in Crohn's disease pathophysiology Ã¢â‚¬â€œ An overview,	https://doi.org/10.1016/j.mce.2021.111448,
Advances in immunotherapy for MMR proficient colorectal cancer,	https://doi.org/10.1016/j.ctrv.2022.102480,
Mouse Models of Psoriasis: A Comprehensive Review,	https://doi.org/10.1016/j.jid.2021.06.019,
Convergence of signalling pathways in innate immune responses and genetic forms of ParkinsonÃ¢â‚¬â„¢s disease,	https://doi.org/10.1016/j.nbd.2022.105721,
Apolipoprotein E in lipid metabolism and neurodegenerative disease,	https://doi.org/10.1016/j.tem.2023.05.002,
Neuroinflammation: The central enabler of postoperative cognitive dysfunction,	https://doi.org/10.1016/j.biopha.2023.115582,
Is Obesity or Adiposity-Based Chronic Disease Curable: The Set Point Theory, the Environment, and Second-Generation Medications,	https://doi.org/10.1016/j.eprac.2021.11.082,
An overview about apitherapy and its clinical applications,	https://doi.org/10.1016/j.phyplu.2022.100239,
Framingham Heart Study: JACC Focus Seminar, 1/8,	https://doi.org/10.1016/j.jacc.2021.01.059,
Dairy Foods and Dairy Fats: New Perspectives on Pathways Implicated in Cardiometabolic Health,	https://doi.org/10.1093/advances/nmz105,
Insulin action at a molecular level Ã¢â‚¬â€œ 100 years of progress,	https://doi.org/10.1016/j.molmet.2021.101304,

Disease control tools to secure animal and public health in a densely populated world,	https://doi.org/10.1016/S2542-5196(22)00147-4,
The importance of nutrition in pregnancy and lactation: lifelong consequences,	https://doi.org/10.1016/j.ajog.2021.12.035,
Nutrition and Nonalcoholic Fatty Liver Disease: Current Perspectives,	https://doi.org/10.1016/j.gtc.2019.09.003,
Advanced preclinical models for evaluation of drug-induced liver injury Ã¢â‚¬â€œ consensus statement by the European Drug-Induced Liver Injury Network [PRO-EURO-DILI-NET],	https://doi.org/10.1016/j.jhep.2021.06.021,
Etiopathogenesis of ovarian cancer. An inflamm-aging entity?,	https://doi.org/10.1016/j.gore.2022.101018,
Inflammatory auto-immune diseases of the intestine and their management by natural bioactive compounds,	https://doi.org/10.1016/j.biopha.2022.113158,
Metabolic Effects of Dietary Nitrate in Health and Disease,	https://doi.org/10.1016/j.cmet.2018.06.007,
The outstanding antitumor capacity of CD4+ T helper lymphocytes,	https://doi.org/10.1016/j.bbcan.2020.188439,
The role of hepatokines in NAFLD,	https://doi.org/10.1016/j.cmet.2023.01.006,
Metabolites as extracellular vesicle cargo in health, cancer, pleural effusion, and cardiovascular diseases: An emerging field of study to diagnostic and therapeutic purposes,	https://doi.org/10.1016/j.biopha.2022.114046,
Therapeutic cancer vaccines revamping: technology advancements and pitfalls,	https://doi.org/10.1016/j.annonc.2021.08.2153,
Migration and Function of Memory CD8+ T Cells in Skin,	https://doi.org/10.1016/j.jid.2019.09.014,
Physical activity and immunity in obese older adults: A systematic bibliographic analysis,	https://doi.org/10.1016/j.smhs.2023.07.001,
The role of MicroRNA networks in tissue-specific direct and indirect effects of metformin and its application,	https://doi.org/10.1016/j.biopha.2022.113130,
A qualitative synthesis of gastro-oesophageal reflux in bronchiectasis: Current understanding and future risk,	https://doi.org/10.1016/j.rmed.2018.06.031,
Maternal vaccination as an additional approach to improve the protection of the nursling: Anti-infective properties of breast milk,	https://doi.org/10.1016/j.clinsp.2022.100093,
The Science and Translation of Lactate Shuttle Theory,	https://doi.org/10.1016/j.cmet.2018.03.008,
The role of host molecules in communication with the resident and pathogenic microbiota: A review,	https://doi.org/10.1016/j.medmic.2020.100005,
Research Opportunities in Autonomic Neural Mechanisms of CardiopulmonaryÃ‚Â Regulation: A Report From the National Heart, Lung, and Blood Institute andÃ‚Â the National Institutes of Health Office of the DirectorÃ‚Â Workshop,	https://doi.org/10.1016/j.jacbts.2021.11.003,
New drug targets for hypertension: A literature review,	https://doi.org/10.1016/j.bbadis.2020.166037,
Associations between diet quality and anxiety and depressive disorders: A systematic review,	https://doi.org/10.1016/j.jadr.2023.100629,
Translational research into frailty from bench to bedside: Salivary biomarkers for inflammaging,	https://doi.org/10.1016/j.exger.2022.112040,

Food made us human: Recent genetic variability and its relevance to the current distribution of macronutrients,	https://doi.org/10.1016/j.nut.2022.111702,
Enhancement of live vaccines by co-delivery of immune modulating proteins,	https://doi.org/10.1016/j.vaccine.2022.08.059,
The find of COVID-19 vaccine: Challenges and opportunities,	https://doi.org/10.1016/j.jiph.2020.12.025,
Machine learning in clinical decision making,	https://doi.org/10.1016/j.medj.2021.04.006,
Metabolic switch in cancer Ã¢â‚¬â€œ Survival of the fittest,	https://doi.org/10.1016/j.ejca.2022.11.025,
Natural therapeutics and nutraceuticalsÃ‚Â for lung diseases: Traditional significance, phytochemistry, and pharmacology,	https://doi.org/10.1016/j.biopha.2022.113041,
Immunotherapy in colorectal cancer: is the long-awaited revolution finally happening?,	https://doi.org/10.1016/j.ctarc.2021.100442,
Unlocking the Complex Flavors of Dysgeusia after Hematopoietic Cell Transplantation,	https://doi.org/10.1016/j.bbmt.2017.10.022,
Mechanisms of Subcutaneous and Sublingual Aeroallergen Immunotherapy: What Is New?,	https://doi.org/10.1016/j.iac.2019.09.009,
Combined mechanistic modeling and machine-learning approaches in systems biology Ã¢â‚¬â€œ A systematic literature review,	https://doi.org/10.1016/j.cmpb.2023.107681,
Immunotherapy-related biomarkers: Confirmations and uncertainties,	https://doi.org/10.1016/j.critrevonc.2023.104135,
Molecular and metabolic heterogeneity of astrocytes and microglia,	https://doi.org/10.1016/j.cmet.2023.03.006,
Personalized medicine: motivation, challenges, and progress,	https://doi.org/10.1016/j.fertnstert.2018.05.006,
Lipid and energy metabolism in Wilson disease,	https://doi.org/10.1016/j.livres.2020.02.002,
Worldwide emergence of fluconazole-resistant Candida parapsilosis: current framework and future research roadmap,	https://doi.org/10.1016/S2666-5247(23)00067-8,
Mapping of Dietary Interventions Beneficial in the Prevention of Secondary Health Conditions in Spinal Cord Injured Population: A Systematic Review,	https://doi.org/10.1007/s12603-023-1937-6,
Interleukin 1ÃŽÂ±: a comprehensive review on the role of IL-1ÃŽÂ± in the pathogenesis and treatment of autoimmune and inflammatory diseases,	https://doi.org/10.1016/j.autrev.2021.102763,
Role of NAD+ in regulating cellular and metabolic signaling pathways,	https://doi.org/10.1016/j.molmet.2021.101195,
Molecular insights into the pathogenic impact of vitamin D deficiency in neurological disorders,	https://doi.org/10.1016/j.biopha.2023.114718,
Lifespan Perspective on CongenitalÃ‚Â Heart Disease Research: JACC State-of-the-Art Review,	https://doi.org/10.1016/j.jacc.2021.03.012,
From congestive hepatopathy to hepatocellular carcinoma, how can we improve patient management?,	https://doi.org/10.1016/j.jhepr.2021.100249,
Colorectal Cancer Disparity in African Americans: Risk Factors and Carcinogenic Mechanisms,	https://doi.org/10.1016/j.ajpath.2017.07.023,
Skin barrier defects in atopic dermatitis: From old idea to new opportunity,	https://doi.org/10.1016/j.alit.2021.11.006,
Neuro-immune crosstalk in depressive symptoms of multiple sclerosis,	https://doi.org/10.1016/j.nbd.2023.106005,
What to tell your patient with clonal hematopoiesis and why: insights from 2 specialized clinics,	https://doi.org/10.1182/blood.2019004291,

Nephrolithiasis: Insights into Biomimics, Pathogenesis, and Pharmacology,	https://doi.org/10.1016/j.ccmp.2022.100077,
Emerging functions and clinical prospects of connexins and pannexins in melanoma,	https://doi.org/10.1016/j.bbcan.2020.188380,
Cytoprotective remedies for ameliorating nephrotoxicity induced by renal oxidative stress,	https://doi.org/10.1016/j.lfs.2023.121466,
Drosophila as a model to explore secondary injury cascades after traumatic brain injury,	https://doi.org/10.1016/j.biopha.2021.112079,
An integrative medicine review of primary prevention of allergy in pediatrics,	https://doi.org/10.1016/j.ctim.2021.102695,
Systemic autoinflammatory diseases,	https://doi.org/10.1016/j.jaut.2020.102421,
Immune checkpoint blockade in HIV,	https://doi.org/10.1016/j.ebiom.2022.103840,
Learning from the nexus of autoimmunity and cancer,	https://doi.org/10.1016/j.immuni.2023.01.022,
The melatonergic pathway and its interactions in modulating respiratory system disorders,	https://doi.org/10.1016/j.biopha.2021.111397,
Pathogen-associated gene discovery workflows for novel antivirulence therapeutic development,	https://doi.org/10.1016/j.ebiom.2022.104429,
16S full-length gene sequencing analysis of intestinal flora in breast cancer patients in Hainan Province,	https://doi.org/10.1016/j.mcp.2023.101927,
Alcohol consumption and metabolic syndrome: Clinical and epidemiological impact on liver disease,	https://doi.org/10.1016/j.jhep.2022.08.030,
Mechanisms of pathogenesis and environmental moderators in preclinical models of compulsive-like behaviours,	https://doi.org/10.1016/j.nbd.2023.106223,
Loss of liver function in chronic liver disease: AnÃ,Â identity crisis,	https://doi.org/10.1016/j.jhep.2022.09.001,
Non-alcoholic fatty liver disease in women Ã¢â,¬â€œ Current knowledge and emerging concepts,	https://doi.org/10.1016/j.jhepr.2023.100835,
A comprehensive review of the advances in neuromyelitis optica spectrum disorder,	https://doi.org/10.1016/j.autrev.2023.103465,
Translational opportunities in the prenatal immune environment: Promises and limitations of the maternal immune activation model,	https://doi.org/10.1016/j.nbd.2020.104864,
Metabolic-associated fatty liver disease: From simple steatosis toward liver cirrhosis and potential complications. Proceedings of the Third Translational Hepatology Meeting, organized by the Spanish Association for the Study of the Liver (AEEH),	https://doi.org/10.1016/j.gastrohep.2022.02.005,
How to isolate, identify and determine antimicrobial susceptibility of anaerobic bacteria in routine laboratories,	https://doi.org/10.1016/j.cmi.2018.02.008,
Pleotropic Effects of Polyphenols in Cardiovascular System,	https://doi.org/10.1016/j.biopha.2020.110714,
Chemotherapeutic drugs: Cell death- and resistance-related signaling pathways. Are they really as smart as the tumor cells?,	https://doi.org/10.1016/j.tranon.2021.101056,
Cholangiocytes in the pathogenesis of primary sclerosing cholangitis and development of cholangiocarcinoma,	https://doi.org/10.1016/j.bbadis.2017.08.020,
Rhythms in barriers and fluids: Circadian clock regulation in the aging neurovascular unit,	https://doi.org/10.1016/j.nbd.2023.106120,

Refining rodent models of spinal cord injury,	https://doi.org/10.1016/j.expneurol.2020.113273,
Incidence of Hepatocellular Carcinoma in Patients With Nonalcoholic Fatty Liver Disease: A Systematic Review, Meta-analysis, and Meta-regression,	https://doi.org/10.1016/j.cgh.2021.05.002,
Development and research progress of anti-drug resistant fungal drugs,	https://doi.org/10.1016/j.jiph.2022.08.004,
Nonalcoholic steatohepatitis and mechanisms by which it is ameliorated by activation of the CNC-bZIP transcription factor Nrf2,	https://doi.org/10.1016/j.freeradbiomed.2022.06.226,
When a calorie is not just a calorie: Diet quality and timing as mediators of metabolism and healthy aging,	https://doi.org/10.1016/j.cmet.2023.06.008,
Issues complicating precision dosing for factor VIII prophylaxis,	https://doi.org/10.1016/j.transci.2018.07.007,
Limitations of non-invasive tests for assessment of liver fibrosis,	https://doi.org/10.1016/j.jhepr.2020.100067,
Metformin: Is it a drug for all reasons and diseases?,	https://doi.org/10.1016/j.metabol.2022.155223,
Beneficial attributes and adverse effects of major plant-based foods anti-nutrients on health: A review,	https://doi.org/10.1016/j.hnm.2022.200147,
Biosynthetic versatility of marine-derived fungi on the delivery of novel antibacterial agents against priority pathogens,	https://doi.org/10.1016/j.biopha.2021.111756,
Link between microbiota and hypertension: Focus on LPS/TLR4 pathway in endothelial dysfunction and vascular inflammation, and therapeutic implication of probiotics,	https://doi.org/10.1016/j.biopha.2021.111334,
Associations between Phytoestrogens, Glucose Homeostasis, and Risk of Diabetes in Women: A Systematic Review and Meta-Analysis,	https://doi.org/10.1093/advances/nmy048,

PRISMA BREAKDOWN

	**results**
PUBMED	343
SCIENCEDIRECT	1337
**TOTAL**	**1680**

DUPLICATES	1221	red colour
**TOTAL MINUS DUPLICATES**	459	
REMOVED FOR REASON 1	82	blue colour
**TOTAL MINUS REASON 1**	**375**	
REMOVED FOR REASON 2	224	orange colour
**TOTAL MINUS REASON 2**	151	
REMOVED FOR REASON 3	82	grey colour
**TOTAL MINUS REASON 3**	67	
REMOVED FOR REASON 4	20	pink colour
REMOVED FOR REASON 5	18	lime colour
REMOVED FOR REASON 6	14	yellow colour
**TOTAL MINUS REASONS 4,5,6**	**17**	purple colour

Reason 1 – The study does not utilize the appropriate design for the review

Reason 2 – The study does not meet the minimum quality threshold based on the assessment criteria

Reason 3 – The study lacks necessary data for extraction and analysis

Reason 4 – The study's follow-up period is too short to assess the long-term outcomes

Reason 5 – The study is a review, commentary, or editorial rather than original research

Reason 6 – Full text is not available
